# Screening HLA-A-restricted T cell epitopes of SARS-CoV-2 and the induction of CD8^+^ T cell responses in HLA-A transgenic mice

**DOI:** 10.1038/s41423-021-00784-8

**Published:** 2021-11-02

**Authors:** Xiaoxiao Jin, Yan Ding, Shihui Sun, Xinyi Wang, Zining Zhou, Xiaotao Liu, Miaomiao Li, Xian Chen, Anran Shen, Yandan Wu, Bicheng Liu, Jianqiong Zhang, Jian Li, Yi Yang, Haibo Qiu, Chuanlai Shen, Yuxian He, Guangyu Zhao

**Affiliations:** 1 Department of Microbiology and Immunology, Medical School of Southeast University, Nanjing, 210009 Jiangsu China; 2 State Key Laboratory of Pathogen and Biosecurity, Beijing Institute of Microbiology and Epidemiology, Beijing, 100071 China; 3 Blood Component Preparation Section, Jiangsu Province Blood Center, Nanjing, 210042 Jiangsu China; 4 Institute of Nephrology, Zhongda Hospital, Medical School of Southeast University, Nanjing, 210009 Jiangsu China; 5 Life Science & Technology School of Southeast University, Nanjing, 210096 Jiangsu China; 6 Jiangsu Province Key Laboratory of Critical Care Medicine, Department of Critical Care Medicine, Zhongda Hospital, Medical School of Southeast University, Nanjing, 210009 Jiangsu China; 7 Institute of Pathogen Biology, Chinese Academy of Medical Sciences and Peking Union Medical College, Beijing, 100730 China

**Keywords:** SARS-CoV-2, T cell epitope, HLA-A, Vaccination, Peptide vaccines, Antimicrobial responses

## Abstract

Since severe acute respiratory syndrome coronavirus-2 (SARS-CoV-2)-specific T cells have been found to play essential roles in host immune protection and pathology in patients with coronavirus disease 2019 (COVID-19), this study focused on the functional validation of T cell epitopes and the development of vaccines that induce specific T cell responses. A total of 120 CD8^+^ T cell epitopes from the E, M, N, S, and RdRp proteins were functionally validated. Among these, 110, 15, 6, 14, and 12 epitopes were highly homologous with SARS-CoV, OC43, NL63, HKU1, and 229E, respectively; in addition, four epitopes from the S protein displayed one amino acid that was distinct from the current SARS-CoV-2 variants. Then, 31 epitopes restricted by the HLA-A2 molecule were used to generate peptide cocktail vaccines in combination with Poly(I:C), R848 or poly (lactic-co-glycolic acid) nanoparticles, and these vaccines elicited robust and specific CD8^+^ T cell responses in HLA-A2/DR1 transgenic mice as well as wild-type mice. In contrast to previous research, this study established a modified DC-peptide-PBL cell coculture system using healthy donor PBMCs to validate the in silico predicted epitopes, provided an epitope library restricted by nine of the most prevalent HLA-A allotypes covering broad Asian populations, and identified the HLA-A restrictions of these validated epitopes using competitive peptide binding experiments with HMy2.CIR cell lines expressing the indicated HLA-A allotype, which initially confirmed the in vivo feasibility of 9- or 10-mer peptide cocktail vaccines against SARS-CoV-2. These data will facilitate the design and development of vaccines that induce antiviral CD8^+^ T cell responses in COVID-19 patients.

## Introduction

Coronavirus disease 2019 (COVID-19), caused by highly contagious severe acute respiratory syndrome coronavirus-2 (SARS-CoV-2), has been spreading worldwide at an unprecedentedly fast pace [1]. As of August 27, 2021, there were more than 214 million confirmed cases and over 4.47 million deaths. Although the current progress in the research and popularization of vaccines has led to better control of the spread of SARS-CoV-2, many countries are experiencing second or third waves of viral disease outbreaks as the virus mutates; thus, the development of effective vaccines is urgently needed to prevent the continued spread of SARS-CoV-2 and its variants. Currently, most vaccines focus on the induction of neutralizing antibodies against the spike (S) protein [2, 3], which can block the virus from entering and infecting human cells, helping the immune system clear the virus and prevent future infections and severe cases [4].

T cells play a critical role in the host defense against many viral infections, particularly CD8^+^ T cells, since CD8^+^ cytotoxic T lymphocytes (CTLs) are vital for the elimination of circulating viruses and virus-infected cells [5]. Human leukocyte antigen (HLA) class I molecules (classically HLA-A, HLA-B, and HLA-C) expressed by virus-infected cells present viral epitope peptides to specific CD8^+^ T cells, thus initiating the activation, proliferation, and differentiation of CTLs. However, HLA alleles are highly polymorphic in the general population, and each HLA allotype presents distinctive antigenic peptides, thus leading to different individuals having distinct strengths for protective or pathogenic immune responses against the same pathogen [6–9]. Although host immunity to SARS-CoV-2 is not fully understood, increasing evidence indicates the important influence of T cells on the outcome after COVID-19 infection [10] and possible long-term protection [11–15]. As a result, T cell immunity is critical in the pathogenesis and immune protection mechanism of COVID-19, thus providing a potential way to develop vaccines and treatments that are effective in the long term [16–23]. Recently, SARS-CoV-2 protein T cell epitopes restricted by H-2 molecules were used to generate Venezuelan equine encephalitis replicon particles expressing a single CD8^+^ or CD4^+^ T cell epitope, which induced robust T cell responses that mediated more rapid viral clearance than neutralizing antibodies and decreased the extent of lung pathological changes in Ad5-hACE2-transduced and SARS-CoV-2-infected mice [24], indicating the potential of T cell epitope vaccines. In addition, an HLA-DR restricted peptide cocktail vaccine from Tubingen University, Germany was enrolled in a phase I clinical trial (NCT04546841). More recently, an engineered vaccine possessing multiple B, CD4^+^, and CD8^+^ T cell epitopes of SARS-CoV-2 was designed and was suggested to produce an effective immune response after vaccine administration, as evaluated by in silico immune simulation studies [25]. Whether a 9- or 10-mer peptide cocktail restricted by HLA class I molecules can elicit SARS-CoV-2-specific CD8^+^ T cell responses in vivo remains unknown.

The identification of T cell epitopes can contribute greatly to the development of T cell epitope vaccines and the precise evaluation of host cellular immunity. To date, numerous T cell epitopes of SARS-CoV-2 proteins have been reported [13, 14, 26–33]. The current study aims to generate a CD8^+^ T cell epitope library covering additional SARS-CoV-2 proteins and the majority of Asian populations. The SARS-CoV-2 proteome consists of 29 proteins, including 4 structural proteins (the envelope protein, E; membrane protein, M; nucleocapsid protein, N; and spike glycoprotein, S) and 25 nonstructural proteins. The former function during the progression of viral assembly and infection of the host, while the latter principally participate in the process of viral replication. Thus, both classes of proteins should have potential utility to develop a vaccine or drug [34]. In Asian populations, particularly Chinese populations, 13 HLA-A allotypes and 32 HLA-B allotypes possess a gene frequency greater than 1% for each allele [35] (http://www.allelefrequencies.net). Here, we first focused on HLA-A allotypes rather than HLA-B allotypes because the former have a much higher gene frequency than the latter. Taken together, this study was dedicated to mapping CD8^+^ T cell epitopes in all of the structural proteins and RNA-dependent RNA polymerases (RdRp, consisting of nsp7, nsp8, and nsp12) and restricted by nine of the most prevalent HLA-A allotypes in Asian populations, which cover over 87% of the Chinese population and approximately 79% of Asian populations. A total of 409 epitopes were predicted in silico and selected as candidate epitopes. Then, the immunogenicity and HLA-A restrictions of 120 epitopes were validated in vitro with DC-peptide-PBL coculture experiments using healthy donor peripheral blood mononuclear cells (PBMCs) and competitive peptide binding experiments. Furthermore, 31 epitopes restricted by the HLA-A0201 molecule were used to generate 9- or 10-mer peptide cocktail vaccines in combination with Poly(I:C), R848, or poly (lactic-co-glycolic acid) nanoparticles (PLGA-NPs) and induced robust SARS-CoV-2-specific CD8^+^ T cell responses in HLA-A2/DR1 transgenic mice and wild-type mice.

## Results

### A total of 409 CD8^+^ T cell epitopes restricted by HLA-A allotypes were predicted in silico from SARS-CoV-2 proteins and selected as candidate epitopes

The potential 9- or 10-mer epitopes from five SARS-CoV-2 proteins (E, M, N, S, and RdRP) restricted by nine of the most prevalent HLA-A allotypes (A0201, A1101, A2402, A0206, A0207, A3303, A3001, A0203, or A1102) were predicted in silico by using five epitope prediction tools and algorithms (IEDB-ANN, IEDB-SMM, SYFPEITHI, EPIJEN, NetMHC, and ConvMHC). For each HLA-A allotype and each protein, the top 1–20 peptides with high affinity were chosen as best putative epitopes according to the following criteria: the binding affinity exceeds the antigenic criteria from at least two algorithms; a ranking in the top 20 as predicted by at least two algorithms; the length of the protein, as long proteins such as S and RdRP should contain more epitopes; and the gene frequency of the HLA-A allotype, as high-frequency allotypes may present more epitopes. In total, 409 peptides were finally selected as candidate epitopes with 45, 63, 71, 130, and 100 epitopes from the E (75 amino acids), M (222 amino acids), N (419 amino acids), S (1273 amino acids), and RdRp (932 amino acids) proteins, respectively. Out of the 409 predicted epitopes, 139 epitopes were common epitopes restricted by several HLA-A allotypes as predicted in silico, so only 270 epitopes needed to be synthesized as peptides for further investigation (Supplementary Table S1).

### The immunogenicity of 120 candidate epitopes was validated by in vitro DC-peptide-PBL costimulation using PBMCs from healthy donors

To validate the immunogenicity of candidate SARS-CoV-2 epitopes, PBMCs from unexposed healthy blood donors were collected, and HLA-A alleles were identified. Mature DCs (mDCs) were successfully induced from adherent PBMCs and coincubated with candidate epitope peptides and autologous PBLs for 14 days followed by IFN-γ intracellular staining (ICS). In partial DC-peptide-PBL cocultures, autologous PBLs were prelabeled with CFSE, followed by the detection of CD8^+^ T cell proliferation after 14 days of coculture. Under the strict gating settings equal to the no peptide control group, when the frequency of IFN-γ^+^ T cells in the CD3^+^/CD8^+^ T cell population was two-fold greater than that of the negative control or the proliferation percentage of CD8^+^ T cells in the CD3^+^/CD8^+^ T cell population increased by 20% or more over the negative control, the candidate epitope peptide in the coculture was defined as antigenic epitope.

**Fig. 1:**
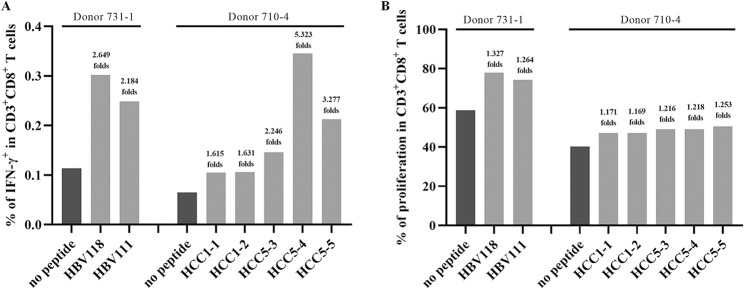
Reference epitope peptides were tested in the DC-peptide-PBL coculture system. The HLA-A0201-restricted HCC1-1, HCC1-2, HCC5-3, HCC5-4, and HCC5-5 peptides and HLA-A2402-restricted HBV111 and HBV118 peptides were cocultured with DCs and PBLs from healthy donor PBMCs with matching HLA-A allotypes for 14 days. **A** The frequency of IFN-γ^+^ T cells in the CD3^+^/CD8^+^ T cell population for each reference epitope peptide and the no peptide negative control. **B** The percent proliferating CD8^+^ T cells in the CD3^+^/CD8^+^ T cell population for each reference epitope peptide and in the no peptide negative control

To evaluate the sensitivity and reliability of this coculture system for T cell epitope screening, seven reference epitope peptides were first tested using this procedure. These reference T cell epitopes derived from hepatocellular carcinoma (HCC)-associated tumor antigens (HCC1-1, HCC1-2, HCC5-3, HCC5-4, and HCC5-5, restricted by HLA-A0201) or from hepatitis B virus antigens (HBV111 and HBV118, restricted by HLA-A2402) have previously been functionally validated in-house to be real-world epitopes because they can effectively stimulate the PBMCs of HCC or chronic hepatitis B patients to produce IFN-γ in an ex vivo 20-h ELISPOT assay and in vitro after costimulation (manuscript submitted). Here, the five reference peptides with strong immunogenicity (spot forming units [SFUs]/2 × 10^5^ PBMCs >15) were defined as antigenic peptides by both IFN-γ ICS and CD8^+^ T cell proliferation assays, while two reference peptides with weak immunogenicity (HCC1-1 and HCC1-2, SFUs/2 × 10^5^ PBMCs <10) were not defined as antigenic peptides (Fig. 1). The flow plots for each reference peptide are shown in Supplementary Fig. S1.

In total, PBMCs from 156 healthy donors were collected to test 270 candidate epitope peptides. In the DC-peptide-PBL coculture system, 62 donor PBMCs responded to the indicated peptides, and 120 peptides were finally defined as antigenic epitope peptides. Of these, 2, 9, 26, and 83 epitope peptides induced positive CD8^+^ T cell responses in the PBMCs of 4 donors, 3 donors, 2 donors, and 1 donor, respectively. For each candidate peptide defined as a negative peptide, at least three donor PBMCs with matching HLA-A allotypes were tested and did not display a significant CD8^+^ T cell response. Table 1 shows the detailed data for each of the 120 validated epitope peptides (VEPs) and each responding donor. The number of validated epitopes derived from the E, M, N, S, and RdRp proteins was 18, 27, 12, 36, and 27, respectively, with a relatively biased distribution. Importantly, 110, 15, 6, 14, and 12 epitopes were highly homologous (deviation of 0–2 amino acids) to SARS-CoV, OC43, NL63, HKU1, and 229E, respectively (Supplementary Table S2). The epitopes in common with common cold HCoVs were mainly located in the RdRp protein (39/47). In addition, four epitopes (D50, D53, D78, or D82) displayed one amino acid that was distinct from the current SARS-CoV-2 variants, such as B.1.1.7, B.1.351, P1, B.1.617, the Denmark variant, B.1.617.2 (Delta), and C.37 (Lambda) (Supplementary Table S2).

**Table 1:** A total of 120 CD8^+^ T cell epitopes of SARS-CoV-2 were validated by in vitro DC-peptide-PBL costimulation experiments

Epitope	In silico predicted HLA-A restriction	Healthy donor 1			Healthy donor 2			Healthy donor 3		
		A allele	Method	Enhancement (fold)	A allele	Method	Enhancement (fold)	A allele	Method	Enhancement (fold)
A1	0201, 0207, 0206, 2402	0201/6843	CFSE	1.244	3101/0207	IFN-γ	2.300	1101/0201	IFN-γ	2.207
A3	0201, 0203, 1101, 1102	0201/2402	IFN-γ	4.142						
A4	0201, 0207, 0203	0201/3303	IFN-γ	2.162	6801/3303	IFN-γ	5.366	2402/0207	IFN-γ	2.373
A5	0201, 0203	0201/3303	IFN-γ	6.892	0201/3001	IFN-γ	7.502	6801/3303	IFN-γ	8.288
A6	0207, 0206	0206/0207	IFN-γ	2.985	1101/0206	IFN-γ	3.630			
A7	0207	0206/0207	IFN-γ	3.538						
A9	0206	0206/1101	IFN-γ	2.632	0206/0203	IFN-γ	2.317	1101/0206	IFN-γ	7.660
A10	0203	0203/3101	IFN-γ	2.091						
A12	1101, 1102, 3303	1101/3001	CFSE	1.221	0201/1101	IFN-γ	2.585	3303/1101	IFN-γ	3.630
A16	1101, 3303	0201/1101	CFSE	1.408						
A18	1102, 3303, 3001	1101/3101	IFN-γ	2.191						
A19	1102	1101/3101	IFN-γ	2.029	1101/2402	CFSE	1.223			
A20	2402	2402/2601	IFN-γ	6.439	2402/2402	IFN-γ	2.461			
A21	2402	2402/2601	IFN-γ	12.544	2402/2402	IFN-γ	2.532			
A22	2402	2402/2601	IFN-γ	4.789						
A23	2402	2402/2601	IFN-γ	4.035	2402/2402	IFN-γ	2.355			
A25	3001	1101/3001	CFSE	2.292						
A26	3001	1101/3001	CFSE	1.395	1101/3001	IFN-γ	3.910			
B1	0201, 0206, 0203	0201/3303	IFN-γ	2.108	0201/3001	IFN-γ	3.229	6801/3303	IFN-γ	4.980
B2	0201, 0206, 0203, 0207	0201/0101	IFN-γ	2.281						
B3	0201, 0207, 0206	0201/3001	IFN-γ	5.073						
B4	0201, 0206	0201/3001	IFN-γ	3.166						
B6	0201, 0207, 0203	0203/0207	IFN-γ	3.421	0201/1101	CFSE	1.357	2402/0207	IFN-γ	2.648
B10	0207	0207/0206	IFN-γ	9.887	3101/0207	IFN-γ	2.200	2402/0207	IFN-γ	2.238
B11	0206	0206/1101	IFN-γ	2.545	0206/3201	CFSE	1.258	1101/0206	IFN-γ	4.070
B12	0206	1101/0206	CFSE	1.225	1101/0206	IFN-γ	2.040			
B15	0203	0203/3101	IFN-γ	5.165						
B16	0203	0203/3001	CFSE	1.247						
B17	0203	0203/3101	IFN-γ	2.227						
B18	1101, 1102	1101/3001	CFSE	1.790						
B20	1101, 1102, 3303	0201/1101	CFSE	1.334						
B21	1101, 1102	0201/1101	CFSE	1.554						
B23	1101, 1102, 3303	3303/1101	IFN-γ	5.315						
B26	2402	2402/2402	IFN-γ	3.145	2402/2601	IFN-γ	3.772			
B28	2402	2402/3303	IFN-γ	3.145						
B29	2402	2402/3303	IFN-γ	6.000	2402/2402	IFN-γ	4.418	6801/3303	IFN-γ	2.425
B30	2402	2402/3303	IFN-γ	4.627						
B31	2402	2402/2402	IFN-γ	2.290	2402/2402	IFN-γ	2.390			
B34	3303	3303/3001	IFN-γ	2.021						
B35	3303	3303/1101	IFN-γ	2.482						
B36	3001	1101/3001	CFSE	1.549	1101/3001	IFN-γ	4.740			
B37	3001	1101/3001	CFSE	1.349	3001/2402	IFN-γ	2.105	1101/3001	IFN-γ	2.910
B38	3001	2402/3001	IFN-γ	2.868						
B40	3001	1101/3001	IFN-γ	2.400						
B41	3001	1101/3001	IFN-γ	5.250						
C1	0201, 0206, 0203	0201/1101	CFSE	1.300						
C3	0201, 0206, 0203	0201/2402	IFN-γ	3.709						
C10	0206	0206/1101	CFSE	1.259						
C12	0206	0206/3201	CFSE	1.208						
C16	0206	0206/1101	IFN-γ	2.877						
C17	0203	0203/3101	IFN-γ	2.574						
C27	1101	1101/1101	CFSE	1.401						
C35	2402	2402/2601	IFN-γ	2.440						
C45	3303	1101/3303	IFN-γ	2.811						
C46	3303	1101/3303	IFN-γ	2.000						
C47	3303	1101/0101	IFN-γ	3.238						
C49	1102	0201/1101	IFN-γ	17.633						
D2	0201, 0206, 0203	0201/1101	IFN-γ	2.223						
D5	0207, 0206, 1102	0206/1102	IFN-γ	5.792						
D6	0201, 0207	0201/2402	CFSE	2.055	0201/0101	IFN-γ	2.069			
D12	0201, 0207, 0206	0201/0201	CFSE	1.234						
D13	0201, 0203	0201/2402	IFN-γ	11.013						
D17	0207, 0203	0206/0207	IFN-γ	2.123						
D26	0206	0201/0207	CFSE	1.299						
D30	0203	0203/0206	CFSE	1.346	0203/0207	IFN-γ	2.453			
D31	0203	0203/0206	CFSE	1.335						
D32	0203	0203/0206	CFSE	1.333						
D33	0203, 0206	0203/0206	CFSE	1.280						
D34	1101/1102	1101/0101	IFN-γ	2.172						
D38	1101, 1102	1101/0101	IFN-γ	2.059						
D40	1101, 1102, 3001	1101/3303	IFN-γ	2.000						
D41	1101	1101/0101	IFN-γ	3.680						
D42	1101, 0206, 0201	1101/0101	IFN-γ	2.284	1101/1101	CFSE	1.476			
D46	1102	0201/1101	IFN-γ	5.586						
D47	1102	0201/1101	IFN-γ	2.799						
D48	1102, 3303	0201/1101	IFN-γ	2.669	3303/1101	IFN-γ	2.178			
D50	1102	0201/1101	IFN-γ	3.420						
D52	2402	2402/3001	IFN-γ	3.417						
D53	2402, 3303	2402/3001	IFN-γ	5.608	3303/1101	IFN-γ	3.755			
D55	2402	2402/0207	IFN-γ	2.102						
D56	2402	2402/2601	IFN-γ	3.669						
D62	2402	2402/3001	IFN-γ	2.824						
D64	2402	0201/2402	IFN-γ	2.449						
D65	3001	0101/3001	CFSE	1.208						
D71	3001	0301/3001	IFN-γ	2.329						
D72	3001, 1102, 0201, 0207	3303/1101	IFN-γ	2.577						
D76	3303	1101/0101	IFN-γ	4.467						
D77	3303, 1102	3303/1101	IFN-γ	2.095	3303/0203	IFN-γ	2.029			
D78	3303, 0203, 0206	3303/1101	IFN-γ	2.014						
D79	3303, 1101, 0207, 2402	3303/1101	IFN-γ	2.758						
D80	3303, 1102, 0201, 0207	3303/1101	IFN-γ	2.482						
D81	3303, 1102, 0203	3303/1101	IFN-γ	2.496						
D82	3303, 1102	3303/1101	IFN-γ	2.017						
R4	0201	0201/1101	IFN-γ	2.078	0201/3201	CFSE	1.473			
R5	0201, 0203	0201/3201	CFSE	2.078	0201/0101	IFN-γ	2.175			
R6	0201, 0207, 0206, 0203	0201/3201	CFSE	1.493						
R8	0201	0201/0101	IFN-γ	2.132						
R9	0201, 0206, 0203	0201/0101	IFN-γ	2.070						
R10	0201	0201/2402	IFN-γ	2.043						
R11	0201	0201/3001	IFN-γ	3.036	0201/1101	IFN-γ	2.044			
R12	0201	0201/3001	IFN-γ	2.470	0201/0101	IFN-γ	2.886			
R13	0201	0201/3001	IFN-γ	2.470	0201/1101	IFN-γ	2.269			
R14	0201, 0203	0201/0101	IFN-γ	2.333						
R15	0201	0201/2402	IFN-γ	3.184						
R17	0207	0207/0206	IFN-γ	2.030						
R23	0206	2402/0207	IFN-γ	2.094						
R24	0203	0203/0206	CFSE	1.608						
R30	1101	1101/3001	CFSE	2.102	0201/1101	CFSE	1.387			
R32	1101/1102	0201/1101	CFSE	1.293						
R34	1101, 3001	0201/1101	CFSE	1.200	1101/3101	IFN-γ	2.221			
R35	1101, 1102	1101/3101	CFSE	1.249						
R38	1101, 3303	0201/1101	CFSE	1.502						
R39	1101, 1102, 3001	0201/1101	CFSE	1.543						
R40	1101, 3001	0201/1101	CFSE	1.397	0201/1101	CFSE	1.195			
R41	1101, 3303	0201/1101	CFSE	1.533						
R42	1101	0201/1101	CFSE	1.210						
R43	1101, 3303	1101/3001	CFSE	1.419	0201/1101	CFSE	1.345			
R44	1101, 1102	0201/1101	CFSE	1.376	0201/1101	CFSE	1.248			
R47	2402	2402/3303	IFN-γ	4.398						
R48	2402	2402/3303	IFN-γ	4.096						

CFSE: after DC-peptide-CFSE-prelabeled PBL cocultures, the percent of proliferating CD8^+^ T cells in the CD3^+^/CD8^+^ population was analyzed according to the reduction in CFSE staining intensity. IFN-γ: after DC-peptide-PBL cocultures, the frequency of IFN-γ^+^/CD8^+^ T cells in the CD3^+^/CD8^+^ population was analyzed by IFN-γ ICS. Enhancement (fold): the fold change in the frequency of IFN-γ^+^/CD8^+^ T cells or percent of proliferating CD8^+^ T cells in the DC-peptide-PBL coculture wells compared with that in the DC-PBL coculture wells without peptide

**Fig. 2:**
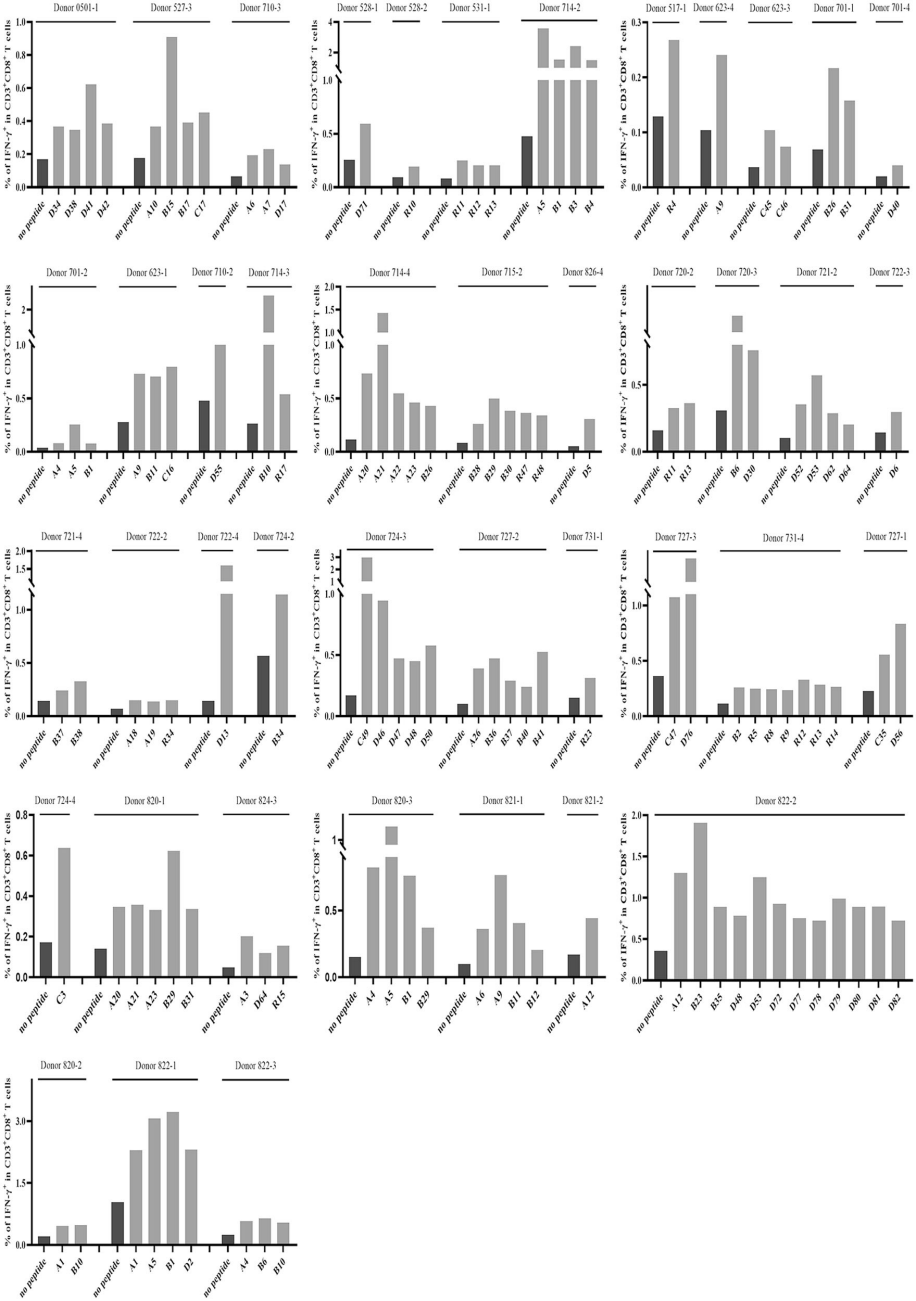
Candidate epitope peptides increased the frequency of IFN-γ^+^/CD8^+^ T cells in DC-peptide-PBL cocultures. DCs were induced for 7 days from healthy donor PBMCs and then coincubated with candidate epitope peptides and autologous PBLs for 14 days. Cells were harvested and stimulated with the corresponding candidate peptides for another 16 h followed by IFN-γ ICS. The frequencies of IFN-γ^+^ T cells in the CD3^+^/CD8^+^ T cell population for each positive epitope peptide and each responding donor are presented as histograms

**Fig. 3:**
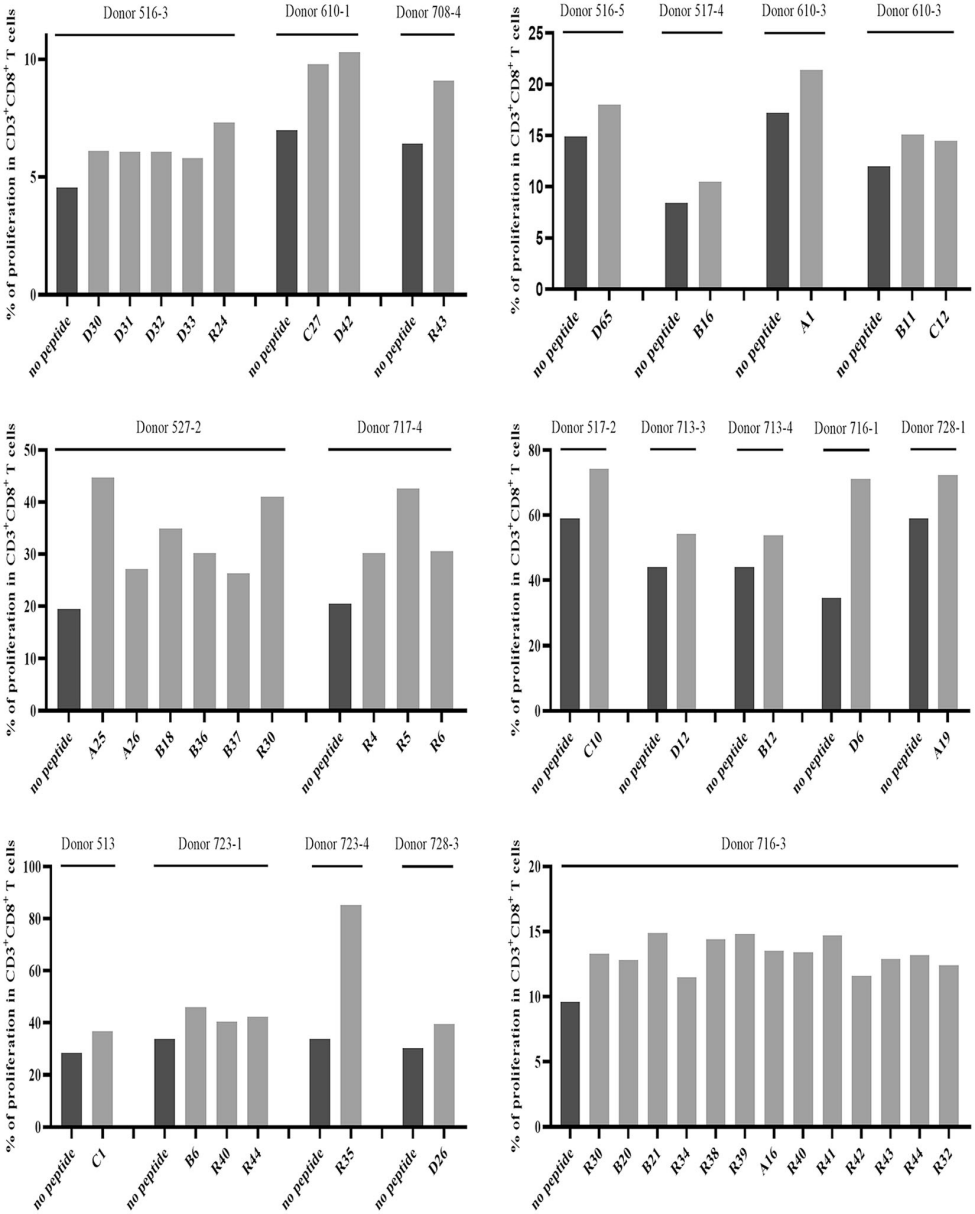
Candidate epitope peptides increased the percent of proliferating CD8^+^ T cells in DC-peptide-PBL cocultures. DCs were induced for 7 days from healthy donor PBMCs and then coincubated with candidate epitope peptides and autologous CFSE-prelabeled PBLs for 14 days. Cells were then harvested followed by flow cytometry analysis. The percent of proliferating CD8^+^ T cells in the CD3^+^/CD8^+^ T cell population for each positive epitope peptide and each responding donor was calculated according to the reduction in CFSE staining intensity and is presented as histograms

Supplementary Fig. S2 shows the phenotypes of mDCs highly expressing CD1a, CD80, CD83, CD86, HLA class I, and HLA-DR molecules, as verified by flow cytometry. Figures 2 and 3 present the frequency of IFN-γ^+^ cells and proliferation percentage of CD8^+^ cells in the CD3^+^/CD8^+^ T cell population for each VEP and each responding donor, respectively. All of the flow plots for the 120 VEPs are displayed in Supplementary Figs. S3 and S4.

### The binding affinities of the validated epitope peptides with predicted HLA-A allotypes were analyzed with a competitive peptide binding assay

HMy2.CIR is a human B lymphocyte cell line that does not express HLA-A or HLA-B molecules and only expresses a trace of HLA-Cw4; thus, HMy2.CIR cells are suitable for host transfection with the HLA class I gene to monitor the affinities of the peptides with the indicated MHC class I molecules [36, 37]. To assess the affinities of the VEPs with predicted HLA-A allotypes, the transfected HMy2.CIR cell lines expressing the indicated HLA-A allotype (A2402, A0201, A0203, A0206, A1101, A3303, A0101, A3001, A0207, or A3101) were generated first, then sorted by flow cytometry and identified by gene sequencing. The purity of these transfected cell lines was 80–94% after sorting (Supplementary Fig. S5).

Then, the unlabeled VEPs of SARS-CoV-2 competed with fluorescently labeled reference peptides for binding to the HLA-A allotype on the surface of the indicated transfected cell lines for 24 h followed by flow cytometry analysis. As shown in Supplementary Fig. S6, most VEPs resulted in a leftward shift of the fluorescent peak derived from the reference peptide, implying the efficient binding of the VEP with the associated HLA-A molecule. Table 2 shows the binding affinity of each VEP with the associated HLA-A allotypes. As shown in Table 3, 54 high-affinity, 39 intermediate affinity, and 37 low-affinity epitopes were identified, but 63 epitopes showed no affinity for the partially predicted HLA-A allotypes relative to the reference peptides. More interestingly, most epitopes (15/18) derived from the E protein (A1–A26) displayed low or no affinity with the predicted HLA-A allotypes in the competitive peptide binding experiments (Table 3), which was discordant with the results from the theoretical predictions, DC-peptide-PBL cocultures, and later, vaccination.

**Table 2:** Binding affinities of 120 VEPs from SARS-CoV-2 with HLA-A allotypes as detected by competitive peptide binding assays using transfected HMy2.CIR cell lines

HLA-A	Epitope	Affinity	5 μM inhibition (%)	15 μM inhibition (%)	Epitope	Affinity	5 μM inhibition (%)	15 μM inhibition (%)
A0201	R12	High	91.70	91.10	R6	No	13.40	23.70
	R8	High	85.50	89.90	D5	Low	10.00	37.80
	B1	High	80	88.20	A5	No	9.40	6.20
	R10	High	75.80	92.80	R13	No	8.66	28.90
	R15	High	70.80	93.80	D80	No	6.38	5.25
	B3	High	66.10	74.40	D42	No	5.93	6.72
	R9	High	64.80	89.50	D72	No	5.93	6.38
	R5	High	58.70	89.10	A4	No	4.80	4.00
	R11	Inter	47.80	86.40	R14	No	4.50	13.30
	B2	Inter	42.10	83.70	A1	No	3.30	2.90
	R4	Inter	40.40	78.30	A3	No	1.90	1.40
	B6	Inter	33.00	65.90	D13	No	0.70	1.20
	D2	Inter	24.50	55.00	C1	No	0	10.30
	D12	Low	20.10	32.60	C3	No	0	7.10
	B4	No	16.70	20.70	D6	No	0	2.10
A1101	D38	High	80.70	87.70	D48	Low	34.38	48.05
	D41	High	80.38	89.72	D50	Low	32.38	43.38
	R43	High	79.50	89.20	B23	Low	32.30	41.00
	R32	High	73.90	89.40	R41	Low	31.90	46.20
	R30	High	72.60	84.90	B18	Low	21.55	34.38
	D34	High	67.70	84.70	D82	Low	20.72	38.22
	B20	High	61.88	55.88	A16	No	18.38	19.22
	D72	High	58.05	76.05	D77	No	15.72	29.38
	D40	High	56.70	82.60	D42	Low	14.70	47.80
	D47	High	53.38	67.72	A12	No	14.38	15.00
	R42	High	50.20	75.20	R38	Low	12.10	47.10
	R44	Inter	47.60	81.4	B21	No	10.55	29.38
	C49	Inter	47.38	74.22	D79	No	10.22	25.22
	D81	Inter	0.4238	52.22	R34	No	10.00	25.90
	D46	Inter	34.38	52.22	D80	No	4.22	5.55
	R40	Inter	30.70	67.10	A19	No	0.20	2.60
	C27	Inter	28.00	54.40	A18	No	0	4.00
	R39	Inter	23.70	53.60				
	R35	Inter	22.50	52.30				
A3303	D80	High	86.40	97.40	B23	Low	21.26	34.07
	D76	High	75.40	94.20	B21	Low	20.36	46.43
	R34	High	72.50	91.00	R38	Low	18.79	42.61
	D77	High	64.20	81.80	D53	Low	14.94	35.87
	R43	High	60.81	65.75	D48	Low	13.39	43.28
	D82	High	49.40	75.90	C46	Low	10.90	42.80
	B34	Inter	41.26	54.29	A18	No	8.90	11.15
	D79	Inter	33.10	62.70	B35	Low	8.10	41.40
	D81	Inter	16.00	74.00	B20	No	7.10	14.74
	C47	Inter	9.90	60.20	A12	No	6.65	0.00
	R41	Inter	8.00	51.15	D78	No	3.30	20.30
	A16	Low	42.61	46.43	C45	No	0.00	29.10
A0203	D30	High	82.80	91.80	B16	Low	38.10	38.00
	R24	High	61.30	78.80	D17	Low	21.83	43.31
	B17	High	57.00	70.50	C3	No	11.44	28.39
	B15	High	50	54.00	B6	Low	6.43	46.41
	D5	High	70.87	83.76	R6	No	5.84	8.10
	R5	High	60.37	81.49	A4	No	4.64	8.94
	R9	High	59.30	81.61	A5	No	5.24	11.09
	D2	High	53.45	55.36	D81	No	4.64	6.79
	B2	High	51.90	68.84	D78	No	3.93	3.21
	D33	Inter	42.30	81.20	D31	No	0	14.80
	B1	Inter	37.82	57.74	A10	No	0	0
	R14	Inter	37.10	65.74	D32	No	0	0
	D13	Inter	25.29	55.48				
	C17	Inter	21.70	51.30				
	C1	Inter	20.39	54.54				
A0206	D26	High	52.30	68.10	C10	Low	17.08	31.68
	R23	Inter	39.30	50.10	D12	No	17.08	29.07
	B3	Inter	42.63	57.11	A1	No	16.30	16.95
	D2	Inter	41.72	63.49	C1	No	15.91	21.77
	B1	Inter	41.20	60.37	A6	No	15.51	13.30
	B2	Inter	37.29	54.89	R6	No	14.73	18.64
	D33	Inter	35.72	68.19	C16	No	12.65	13.69
	R9	Inter	30.12	73.53	C3	No	12.65	13.30
	C12	Low	19.43	36.25	D42	No	9.26	12.78
	B4	No	18.77	14.34	D78	No	8.47	8.34
	B11	Low	18.30	43.40	A9	No	0	10.20
	B12	Low	17.00	32.60				
A2402	B30	High	88.65	91.49	B28	No	18.00	28.20
	R47	High	88.50	88.70	A22	No	10.70	29.30
	B26	High	83.60	82.60	D52	No	6.25	24.78
	B31	High	81.10	86.30	A1	No	5.60	7.20
	D55	High	73.07	78.50	A20	No	5.30	21.30
	B29	High	70.10	74.90	C35	No	2.80	10.10
	D53	High	59.85	62.21	A23	No	1.20	1.20
	R48	Inter	40.10	51.00	A19	No	0.00	0.00
	D62	Inter	37.53	75.19	A21	No	0.00	0.00
	D64	Inter	35.05	69.41	D79	No	0.00	0.00
	D56	Low	30.21	45.80				
A0207	A7	High	68.52	69.58	R6	Low	35.22	48.64
	B2	High	63.58	64.64	D72	Low	32.87	41.69
	R17	High	57.11	62.75	D80	Low	20.64	47.93
	B6	High	56.40	60.87	D79	No	18.75	29.58
	A6	High	54.99	62.05	D17	No	16.40	21.11
	B3	High	54.99	63.34	D5	Low	11.46	44.64
	A1	Inter	45.22	50.16	B10	No	2.40	0.00
	D6	Low	46.40	48.05	D12	No	0.00	0.00
	A4	Low	36.40	40.40				
A3001	D65	High	75.62	79.92	D72	Inter	44.70	62.45
	D71	High	72.12	65.13	R34	Inter	31.53	54.11
	B40	High	63.52	72.12	R40	Low	38.25	29.92
	B41	High	60.30	60.30	A26	Low	37.18	18.90
	B36	High	60.03	73.20	D40	Low	36.91	42.55
	B37	High	54.11	60.03	A25	Low	20.51	33.15
	B38	Inter	49.54	61.91	A18	Low	15.94	35.83
	R39	Inter	46.05	55.46				

Binding affinity of the epitope peptide with indicated HLA-A allotype was assessed by IC50 value, which is the concentration of unlabeled competitor peptide required to inhibit the binding of fluorescently labeled reference peptide by 50%. IC50 < 5 μM (5 μM inhibition >50%) means high binding affinity; 5 μM < IC50 < 15 μM (5 μM inhibition < 50% but 15 μM inhibition > 50%) means intermediate binding affinity; IC50 > 15 μM means low or no binding affinity (5 μM inhibition 20–50% or 15 μM inhibition 30–50% means low binding affinity and 5 μM inhibition < 20% or 15 μM inhibition < 30% means no binding affinity)

**Table 3:** HLA-A restrictions of the 120 VEPs from SARS-CoV-2

Epitope	In silico predicted HLA-A restriction	HLA-A restriction identified by competitive peptide binding assay				No test
		High affinity	Intermediate affinity	Low affinity	No affinity	
A1	A0201, A0207, A0206 A2402		A0207		A0206 > A2402 > A0201	
A3	A0201				A0201	
A4	A0201, A0207, A0203			A0207	A0201 > A0203	
A5	A0201, A0203				A0201 > A0203	
A6	A0207, A0206	A0207			A0206	
A7	A0207	A0207				
**A9**	A0206				A0206	
**A10**	A0203				A0203	
**A12**	A1101, A1102, A3303				A1101 > A3303	A1102
A16	A1101, A3303			A3303	A1101	
A18	A1102, A3303, A3001			A3001	A3303 > A1101	A1102
A19	A1102				A1101	A1102
**A20**	A2402				A2402	
**A21**	A2402				A2402	
**A22**	A2402				A2402	
**A23**	A2402				A2402	
A25	A3001			A3001		
A26	A3001			A3001		
B1	A0201, A0206, A0203	A0201	A0206 > A0203			
B2	A0201, A0206, A0203, A0207	A0207 > A0203	A0201 > A0206			
B3	A0201, A0207, A0206	A0201 > A0207	A0206			
**B4**	A0201, A0206				A0206 > A0201	
B6	A0201, A0207, A0203	A0207	A0201	A0203		
**B10**	A0207				A0207	
B11	A0206			A0206		
B12	A0206			A0206		
B15	A0203	A0203				
B16	A0203			A0203		
B17	A0203	A0203				
B18	A1101, A1102			A1101		A1102
B20	A1101, A1102, A3303	A1101			A3303	A1102
**B21**	A1101, A1102				A1101	A1102
B23	A1101, A1102, A3303			A1101 > A3303		A1102
B26	A2402	A2402				
**B28**	A2402				A2402	
B29	A2402	A2402				
B30	A2402	A2402				
B31	A2402	A2402				
B34	A3303		A3303			
B35	A3303			A3303		
B36	A3001	A3001				
B37	A3001	A3001				
B38	A3001		A3001			
B40	A3001	A3001				
B41	A3001	A3001				
C1	A0201, A0206, A0203		A0203		A0206 > A0201	
**C3**	A0201, A0206, A0203				A0206 > A0203 > A0201	
C10	A0206			A0206		
C12	A0206			A0206		
C16	A0206				A0206	
C17	A0203		A0203			
C27	A1101		A1101			
**C35**	A2402				A2402	
**C45**	A3303				A3303	
C46	A3303			A3303		
C47	A3303		A3303			
C49	A1102		A1101			A1102
D2	A0201, A0206, A0203	A0203	A0206 > A0201			
D5	A0201, A0207, A0203	A0203		A0207 > A0201		
D6	A0201, A0207			A0207	A0201	
D12	A0201, A0207, A0206		A0206	A0201	A0207	
D13	A0201, A0203		A0203		A0201	
D17	A0207, A0203			A0203	A0207	
D26	A0206	A0206				
D30	A0203	A0203				
**D31**	A0203				A0203	
**D32**	A0203				A0203	
D33	A0203, A0206		A0203 > A0206			
D34	A1101, A1102	A1101				A1102
D38	A1101, A1102	A1101				A1102
D40	A1101, A1102, A3001	A1101		A3001		A1102
D41	A1101	A1101				
D42	A1101, A0206, A0201			A1101	A0206 > A0201	
D46	A1102		A1101			A1102
D47	A1102	A1101				A1102
D48	A1102, A3303			A1101 > A3303		A1102
D50	A1102			A1101		A1102
D52	A2402				A2402	
D53	A2402, A3303	A2402		A3303		
D55	A2402	A2402				
D56	A2402			A2402		
D62	A2402		A2402			
D64	A2402		A2402			
D65	A3001	A3001				
D71	A3001	A3001				
D72	A3001, A1102, A0201, A0207	A1101	A3001	A0207	A0201	
D76	A3303	A3303				
D77	A3303, A1102	A3303			A1101	A1102
**D78**	A3303, A0203, A0206				A0206 > A0203 > A3303	
D79	A3303, A1101, A0207, A2402		A3303		A0207 > A1101 > A2402	
D80	A3303, A1102, A0201, A0207	A3303		A0207	A0201 > A1101	A1102
D81	A3303, A1102, A0203		A1101 > A3303		A0203	A1102
D82	A3303, A1102	A3303		A1101		A1102
R4	A0201		A0201			
R5	A0201, A0203	A0203 > A0201				
R6	A0201, A0207, A0206, A0203			A0207 >	A0206 > A0201 > A0203	
R8	A0201	A0201				
R9	A0201, A0206, A0203	A0201 > A0203	A0206			
R10	A0201	A0201				
R11	A0201		A0201			
R12	A0201	A0201				
R13	A0201				A0201	
R14	A0201, A0203		A0203		A0201	
R15	A0201	A0201				
R17	A0207	A0207				
R23	A0206		A0206			
R24	A0203	A0203				
R30	A1101	A1101				
R32	A1101, A1102	A1101				A1102
R34	A1101, A3001, A3303	A3303	A3001		A1101	
R35	A1101, A1102		A1101			A1102
R38	A1101, A3303			A3303 > A1101		
R39	A1101, A1102, A3001		A1101			A1102
R40	A1101, A3001		A1101	A3001		
R41	A1101, A3303		A3303	A1101		
R42	A1101	A1101				
R43	A1101, A3303	A1101 > A3303				
R44	A1101, A1102		A1101			A1102
R47	A2402	A2402				
R48	A2402		A2402			

The 17 epitopes highlighted in bold displayed no affinity for the predicted HLA-A allotypes. “>” indicates the affinity from high to low

### The validated epitope peptides were restricted by the HLA-A0201 molecule

As validated by the DC-peptide-PBL cocultures, 31 candidate epitope peptides restricted by the HLA-A0201 allotype were defined as antigenic peptides. Their binding affinities with predicted HLA-A allotypes were further analyzed using competitive peptide binding experiments with the transfected HMy2.CIR cell lines. Eight high-affinity, 5 intermediate affinity, 2 low-affinity, and 15 no-affinity epitopes were defined with the HLA-A0201 molecule, and 15 epitope peptides could cross-bind with HLA-A0203, HLA-A0206, or HLA-A0207 with high or intermediate affinity (Table 3 and Supplementary Table S3). Moreover, a T2 cell binding and HLA-A2 stability assay was used to further define the affinities of the 31 epitopes for the HLA-A0201 molecule, since this assay has experimental principles and affinity algorithms that are different from the previous assay. T2 cells were incubated with each VEP or without peptide and β2-microglobulin for 16 h. Then, peptide-induced upregulation of HLA-A0201 expression on T2 cells was measured by PE-labeled anti-HLA-A2.1 antibody staining and flow cytometry (Supplementary Fig. S7). Eighteen high-affinity, seven intermediate affinity, four low-affinity, and two no-affinity epitopes were defined for the HLA-A0201 molecule (Supplementary Table S4). These results were more consistent with those from the in silico predictions. As summarized in Supplementary Table S3, the data from the competitive peptide binding experiments were clearly inconsistent with those from other approaches, possibly because the binding affinities determined by the competitive peptide binding assay were calculated relative to the binding affinity of reference peptides. The correlation coefficient between the competitive peptide binding and T2 cell binding assays was analyzed, giving values of *r* = 0.3807 at 5 μM inhibition or 0.3766 at 15 μM inhibition, *p* = 0.05 (Supplementary Fig. S8A). No significant correlations between predicted affinity (ANN, nM) and competitive peptide binding or T2 cell binding were found (Supplementary Fig. S8B, C).

### Peptide cocktail vaccines induced robust specific CD8^+^ T cell responses in HLA-A2/DR1 transgenic mice

To determine whether these VEPs validated by the in vitro DC-peptide–PBL cocultures can stimulate T cell responses in vivo, 31 VEPs restricted by the HLA-A0201 molecule were grouped into four peptide pools (Supplementary Table S5) and used to generate peptide cocktail vaccines into the following three formulations: peptide-encapsulated and peptide-surface coupled PLGA-NPs/peptides (Vaccine A), R848/peptides (Vaccine B), and poly I:C/peptides (Vaccine C) (Supplementary Table S6). Finally, HLA-A0201^+/+^/DR1^+/+^ transgenic and H-2-β2m^–/–^/I-Aβ^–/–^ C57BL/6 mice were immunized with the three vaccines. After three rounds of in vivo vaccination, the splenocytes of the primed mice were tested for VEP-specific T cell responses by IFN-γ ELSPOT, IFN-γ ICS, and IFN-γ ELISA.

**Fig. 4:**
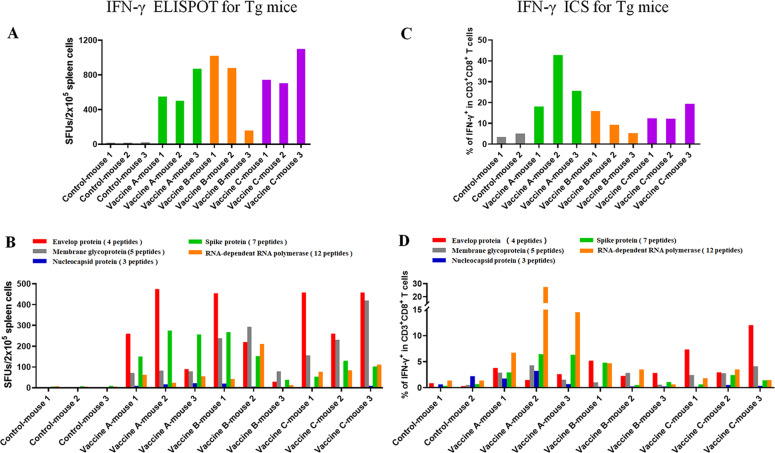
T cell epitope peptide cocktail vaccines elicited robust CD8^+^ T cell responses in transgenic mice. Thirty-one VEPs restricted by the HLA-A0201 molecule were used to generate three formulations of peptide cocktail vaccines, followed by three rounds of immunizations to HLA-A2/DR1 transgenic C57BL/6 mice. Then, splenocytes were collected 7 days after the last booster and stimulated ex vivo overnight with distinct peptide pools according to single protein followed by IFN-γ ELISPOT and IFN-γ ICS. **A** Total IFN-γ SFUs responding to all peptide pools in each mouse. **B** Deconvolution of the total SFUs in each mouse from (**A**) into the single SARS-CoV-2 proteins. **C** Total frequency of IFN-γ^+^ T cells reacting to all peptide pools in the CD3^+^CD8^+^ T cell population in each mouse. **D** Deconvolution of the total frequency in each mouse from (**C**) into the single SARS-CoV-2 proteins. Control groups: N.S and PLGA-NPs; Vaccine A group: PLGA-NPs/peptides vaccines; Vaccine B group: R848/peptides vaccines; Vaccine C group: poly I: C/peptides vaccines

**Fig. 5:**
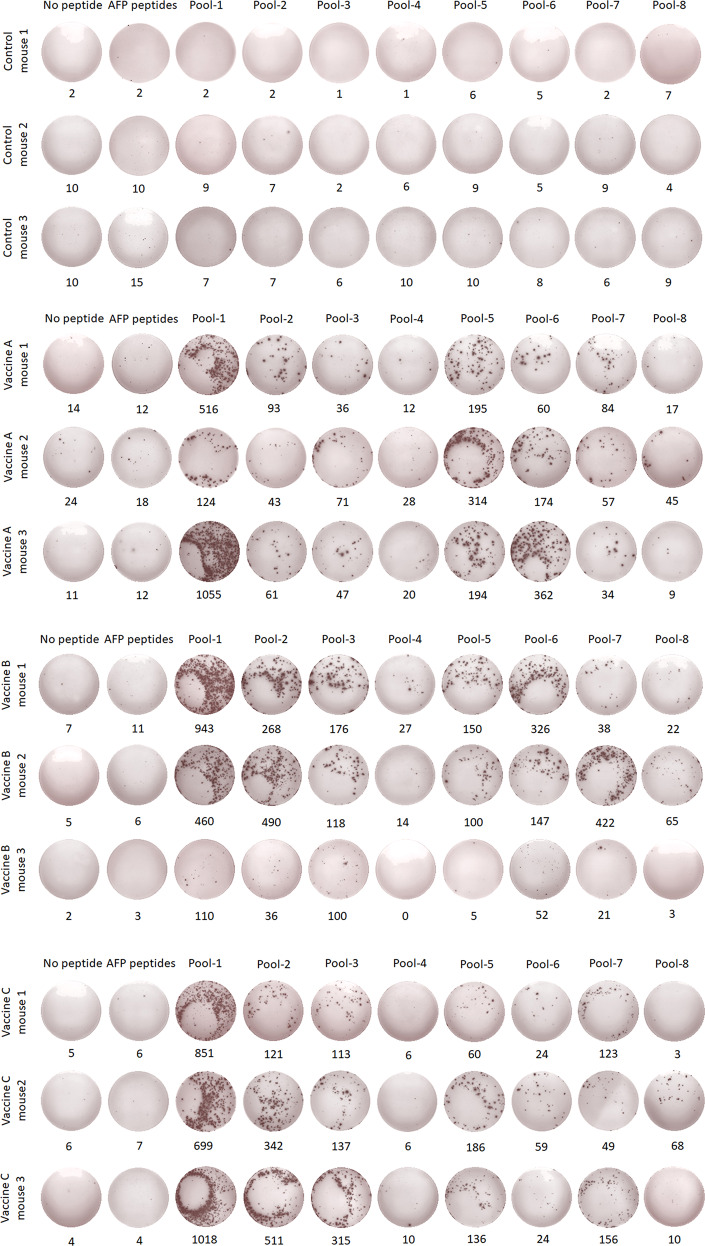
IFN-γ ELISPOT spot plots against the individual peptide pools in transgenic mice. Splenocytes from each primed transgenic mouse were harvested 7 days after the last booster and stimulated ex vivo with eight different peptide pools covering the 31 VEPs, AFP peptides (AFP_158–166_, AFP_424–432_) as irrelevant controls or without peptide as a negative control, followed by IFN-γ ELISPOT. Control group: N.S and PLGA-NPs; Vaccine A group: PLGA-NPs/peptides vaccines; Vaccine B group: R848/peptides vaccines; Vaccine C group: poly I: C/peptides vaccines

The 31 VEPs were grouped into eight pools (Supplementary Table S5) according to their derived proteins and their acidic and alkaline features. Then, splenocytes from each mouse were coincubated with each peptide pool or PBS for 20 h in a 96-well PVDF membrane plate, followed by IFN-γ ELSPOT assay. The total number of SFUs in 2 × 10^5^ splenocytes from each mouse was 400–500 times greater in the three vaccine groups than in the control group (Fig. 4A, B). Figure 5 presents the results of the ELISPOT assay from all mice. Two irrelevant CD8^+^ T cell epitope peptides (AFP_158–166_ and AFP_424–432_) were used as antigen-irrelevant control groups and gave negative results similar to those of the no peptide group.

**Fig. 6:**
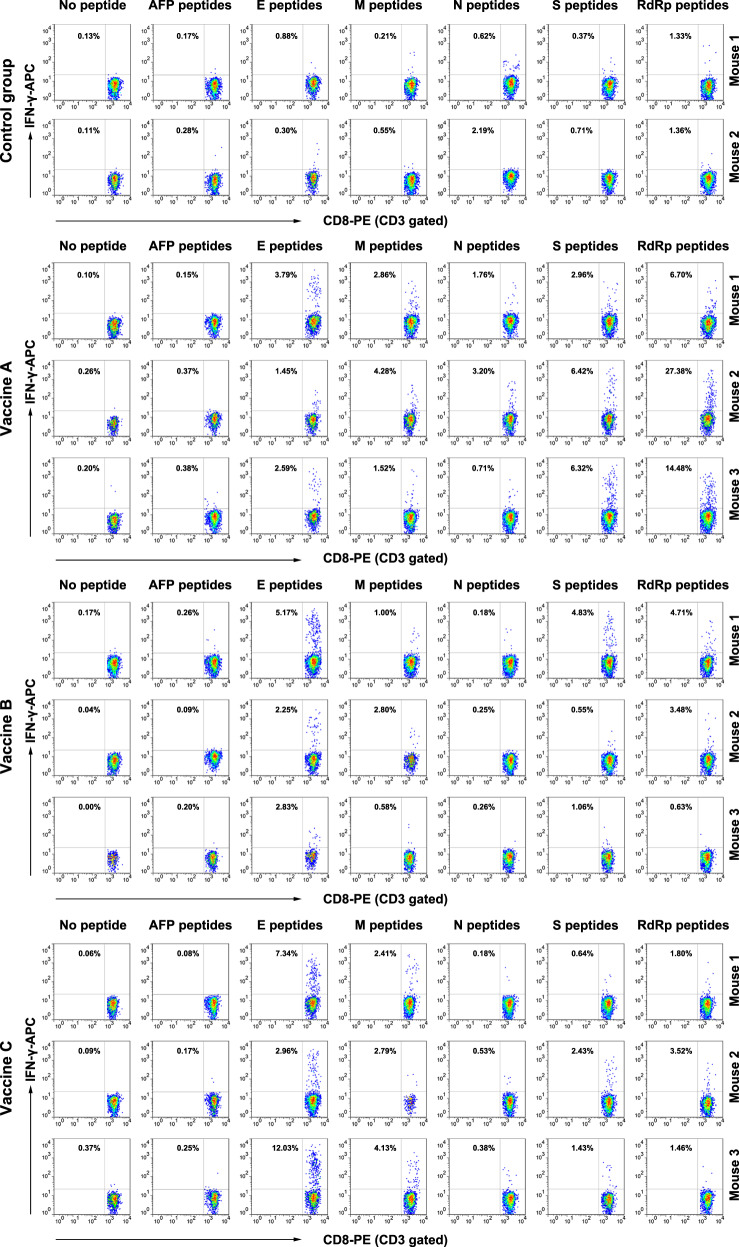
Flow plots of the IFN-γ ICS response to the individual peptide pools in transgenic mice. Splenocytes from each primed mouse were harvested 7 days after the last booster and stimulated ex vivo with five different peptide pools according to a single protein, AFP peptides (AFP_158–166_, AFP_424–432_) as irrelevant controls, or without peptide as negative control, followed by IFN-γ ICS. The data in the left upper quadrant indicate the frequencies of IFN-γ^+^ T cells in the CD3^+^/CD8^+^ cell populations. Control group: N.S and PLGA-NPs; Vaccine A group: PLGA-NPs/peptides vaccines; Vaccine B group: R848/peptides vaccines; Vaccine C group: poly I: C/peptides vaccines

To further confirm whether specific CD8^+^ T cell responses were elicited, IFN-γ ICS was performed. The 31 VEPs were grouped into five pools (Supplementary Table S5) according to their derived proteins. Then, splenocytes from each mouse were coincubated with each peptide pool or PBS for 16 h in a 48-well plate, followed by another 6 h of coincubation with a BFA/monensin mixture. The resulting ICS showed that the frequencies of IFN-γ^+^ cells in the CD3^+^CD8^+^ T cell populations from each vaccine group were approximately 20–30 times higher than that in the control mice (Fig. 4C, D). Figure 6 presents the ICS flow plots for all mice. In addition, two irrelevant CD8^+^ T cell epitope peptides (AFP_158–166_ and AFP_424–432_) were used and gave results similar to those of the no peptide group.

**Fig. 7:**
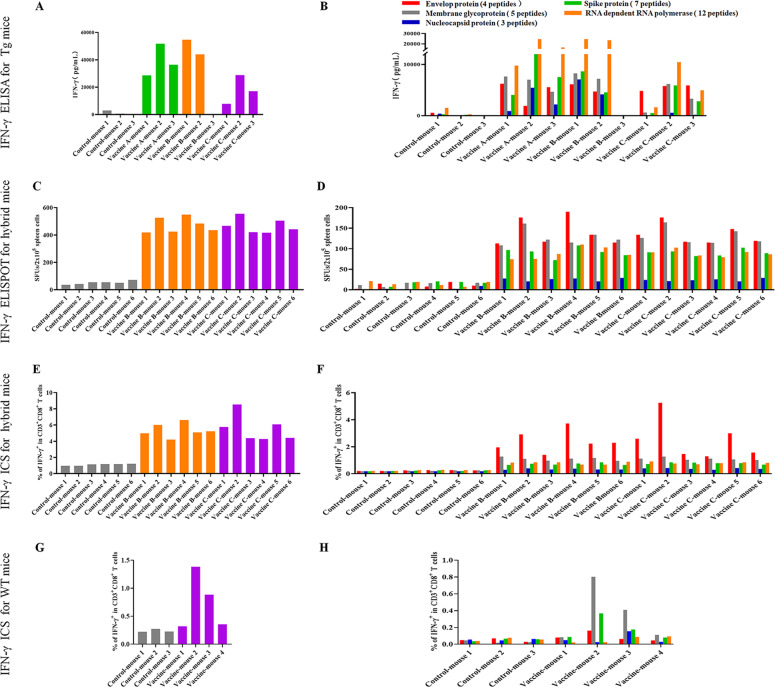
T cell epitope peptide cocktail vaccines elicited robust CD8^+^ T cell responses in transgenic mice, hybrid mice, and WT mice. Splenocytes from each primed mouse were harvested 7 days after the last booster and stimulated ex vivo with different peptide pools according to a single protein or without peptide. **A** Total IFN-γ levels in the supernatants responding to all peptide pools in each Tg mouse as detected by IFN-γ ELISA after 3 days of coculture. **B** Deconvolution of the total IFN-γ level in each Tg mouse from (**A**) into the individual SARS-CoV-2 proteins. **C** Total IFN-γ SFUs responding to all peptide pools in each hybrid mouse after 20 h of coculture. **D** Deconvolution of the total SFUs in each hybrid mouse from (**C**) into the individual SARS-CoV-2 proteins. **E** Total frequency of IFN-γ^+^ T cells reacting to all peptide pools in the CD3^+^CD8^+^ T cell population in each hybrid mouse after 22 h of coculture. **F** Deconvolution of the total frequency in each hybrid mouse from (**E**) into the individual SARS-CoV-2 proteins. **G** Total frequency of IFN-γ^+^ T cells reacting to all peptide pools in the CD3^+^CD8^+^ T cell population in each WT mouse after 22 h of coculture. **H** Deconvolution of the total frequency in each mouse from (**G**) into the individual SARS-CoV-2 proteins. Tg mice: HLA-A0201^+/+^/DR1^+/+^ transgenic and H-2-β2m^–/–^/I-Aβ^–/–^ C57BL/6 mice; hybrid mice: hybrid of HLA-A0201^+/+^/DR1^+/+^/H-2-β2m^–/–^/I-Aβ^–/–^ C57BL/6 mice and WT C57BL/6 mice; WT mice: wild-type C57BL/6 mice; Control group: normal saline plus PLGA-NPs in Tg mice or normal saline in other mice; Vaccine A group: PLGA-NPs/peptides vaccines; Vaccine B group: R848/peptides vaccines; Vaccine C group: poly I: C/peptides vaccines

Furthermore, ELISA was carried out to quantify IFN-γ in the supernatant after the splenocytes were incubated for 72 h with each of the five peptide pools or PBS in a 48-well plate. The accumulation of IFN-γ in each vaccine group was approximately 15–30 times higher than that in the control group (Fig. 7A, B), which is consistent with the ELISPOT and ICS results.

Notably, mouse-3 in the Vaccine B group (R848/peptide vaccine) showed only weak or no T cell responses, as detected by ELSPOT, ICS, and ELISA. This failure may be due to the weak reactivity of the overall T cell repertoire since the SFU of 2 × 10^5^ splenocytes was much less than that of other primed mice after stimulation with the mitogen PHA, as detected by ELISPOT (70 vs. 832.2 ± 328.9). To further confirm the in vivo results, administration of Vaccine B (R848/peptides) and Vaccine C (poly I:C/peptides) was repeated in the hybrid HLA-A0201^+/+^/DR1^+/+^/H-2-β2m^–/–^/I-Aβ^–/–^ C57BL/6 mice and WT C57BL/6 mice, which induced a similar trend of robust CD8^+^ T cell responses. Each group consisted of six hybrid mice expressing HLA-A0201 and DR1 molecules on splenocytes, as confirmed by flow cytometry. IFN-γ ELISPOT showed that the SFUs in 2 × 10^5^ splenocytes from each mouse were approximately nine times higher in the Vaccine B and C groups than that in the control group (Fig. 7C, D). The frequencies of IFN-γ^+^ cells in the CD3^+^CD8^+^ T cell populations from each Vaccine B and C group reached approximately five times that of the control mice, as detected by IFN-γ ICS (Fig. 7E, F). Supplementary Figs. S9 and S10 present the results of the ELISPOT assay and the flow plots from ICS for all mice, respectively. In addition, two irrelevant CD8^+^ T cell epitope peptides (AFP_158–166_ and AFP_424–432_) were used and gave results similar to those of the no peptide group. In conclusion, the vaccines elicited VEP-specific CD8^+^ T cell responses that were slightly weaker in the hybrid mice than in the homozygous transgenic mice.

### The peptide cocktail vaccine induced CD8^+^ T cell responses in wild-type C57BL/6 mice

To investigate whether the HLA-A2-restricted 9- or 10-mer peptides can also be cross-presented by mouse H-2K/D^b^ molecules, wild-type C57BL/6 mice were immunized with Vaccine C (peptide pool-v1, pool-v2, pool-v3, and pool-v4 mixed with poly I:C). After three rounds of in vivo stimulation according to the timeline for HLA-A2/DR1 transgenic mice, splenocytes from primed C57BL/6 mice were detected by IFN-γ ICS. The frequencies of IFN-γ^+^ cells in the CD3^+^/CD8^+^ population were four to seven times higher in two of four groups of vaccinated mice than in the control group (Fig. 7G, H). Supplementary Fig. S11A presents the flow plots of all mice. In addition, two irrelevant CD8^+^ T cell epitope peptides (AFP_158–166_ and AFP_424–432_) were used and gave results similar to those of the no peptide group.

To further identify the epitopes cross-presented by mouse H-2K/D^b^ molecules, splenocytes from primed Vaccine mouse-2 and Vaccine mouse-3 were coincubated with each of the 31 VEPs followed by IFN-γ ICS. Seven of the 31 VEPs (A5, B1, B2, B6, C2, D6, and D13) increased the frequency of IFN-γ^+^ T cells in the CD3^+^/CD8^+^ population by two-fold compared with that in the no peptide ex vivo stimulation group (Supplementary Fig. S11B).

### T cell epitope-based peptide cocktail vaccines do not lead to visible organ toxicity

To determine whether the peptide-based vaccines cause organ toxicity, the heart, liver, lung, and kidney from each mouse were examined at day 28 after the HLA-A0201^+/+^/DR1^+/+^ transgenic and H-2-β2m^–/–^/I-Aβ^–/–^ C57BL/6 mice were inoculated three times with Vaccine A, Vaccine B, or Vaccine C. The organs were immersed and stained with hematoxylin-eosin. As the scanning copy showed, no visible organ toxicity was found in any of the organs in each group (Supplementary Fig. S12).

## Discussion

SARS-CoV-2 vaccine development is of major importance for COVID-19 control but is mainly biased toward neutralizing antibody protection, which generally less effectively elicits CD8^+^ T cell responses and faces possible risks during the clearing of the virus and preventing infection. Informed by the protective immunity observed after natural infection, vaccine approaches that elicit antiviral SARS-CoV-2-specific CD4^+^ and CD8^+^ T cells in coordination with neutralizing antibodies generate more robust and durable protective immunity [23, 38]. Memory T cell responses can persist for 6–17 years after SARS-CoV infection [13, 39] and, in mice, protect against lethal virus challenge [40]. In contrast, memory B cells live for only a short period of time in the host [39, 40].

This study aimed to screen CD8^+^ T cell epitopes of SARS-CoV-2 and develop T cell epitope vaccines, providing several notable points. First, this study focused on a series of high-frequency HLA-A allotypes, encompassing a total HLA-A allele frequency of approximately 87% of the Chinese population and 79%, 78%, 63%, 59.5%, 49.5%, and 46.5% of the populations in Southeast Asia, Northeast Asia, Indonesia, South America, Europe, and North America respectively (http://www.allelefrequencies.net). Thus, we provided a library of CD8^+^ T cell epitopes that not only covers broad antigenic targets by recognized CD8^+^ T cell clones but also fits the HLA genetic features of the Asian population, therefore facilitating the design and development of SARS-CoV-2 vaccines inducing antiviral CD8^+^ T cell responses.

Second, this study established a DC-peptide-PBL coculture system using unexposed healthy donor PBMCs to validate the CD8^+^ T cell epitopes predicted in silico. The most reliable and valuable method to validate the immunogenicity of T cell epitopes is detecting epitope-specific memory T cell clones in PBMCs or other cell samples from COVID-19 patients or convalescent humans. However, it is currently not very practicable to collect a large cohort of patient blood samples in China. As an alternative approach, we used PBMCs from unexposed healthy donors to establish a 14-day DC-peptide-PBL coculture system because DC-T or peptide-PBMC coculture procedures using unexposed donor PBMCs have previously been used to validate CD4^+^ T cell epitopes of SARS-CoV-2 with an approximately ten-fold enhancement of T cell activation [31]. In our coculture system, the 9- or 10-mer peptides were maintained in culture media at a high concentration (20 μg/mL) for 14 days and may be engulfed by DCs and cross-presented to CD8^+^ T cells by HLA-A molecules or directly bind to the HLA-A molecules on DCs and B cells followed by recognition by naive CD8^+^ T cells. Naive T cells are unlikely to be activated and proliferate at a similarly high level compared to SARS-CoV-2-specific memory T cells after costimulation of COVID-19 patient PBMCs with peptide [41]. Therefore, although many peptides can increase the frequency of IFN-γ^+^/CD8^+^ T cells by three to five times compared with the control group, this DC-peptide-PBL coculture system inevitably cannot find all of the immunogenic epitopes, in particular, weak epitopes, due to the lower sensitivity of this system. In addition, not all antigenic peptides validated in this coculture system will be real-world epitopes after natural infection, and their immunogenicity needs to be further confirmed by detecting specific T cell clones in SARS-CoV-2-infected persons.

Notably, of the 120 CD8^+^ T cell epitopes validated in the DC-peptide-PBL cocultures, 83 epitopes induced positive CD8^+^ T cell responses in the PBMCs from only one donor. However, this does not mean that these epitopes are not prevalent epitopes in SARS-CoV-2-infected patients because they elicited naive T cell clones from unexposed donors rather than stimulating memory T cells from SARS-CoV-2-infected patients. In our opinion, the peptides eliciting a T cell response in PBMCs from only one unexposed donor should be defined as antigenic peptides, so they may not have been tested again using PBMCs from more donors in this study. This is particularly true for epitopes restricted by relatively low-frequency HLA-A allotypes due to the lack of healthy donors carrying these HLA-A alleles.

More interestingly, 44 (36.66%) of the 120 validated CD8^+^ T cell epitope peptides simultaneously elicited naive CD4^+^ T cells with a two- to six-fold increase in IFN-γ^+^/CD8^–^/CD3^+^ T cell frequency or a more than 20% increase in CD8^–^/CD3^+^ T cell proliferation in DC-peptide-PBL cocultures (Supplementary Fig. S13A, B). Inconsistent with these in vitro data, only very weak CD8^-^/CD3^+^ T cell responses were found in the HLA-A2/DR1 transgenic mice vaccinated with the 31 VEP cocktails, as detected by IFN-γ ICS (Supplementary Fig. S13C, D). These data suggest that a high concentration of short epitope peptides (20 μg/mL) may also be presented to CD4^+^ T cells by HLA class II molecules on DCs and B cells in the microculture system but not under in vivo conditions. The underlying mechanism remains to be further elucidated.

In addition, this study initially confirmed the in vivo feasibility of 9- or 10-mer peptide cocktail vaccines of SARS-CoV-2. HLA class II molecule-restricted peptides (15- or 16-mers) can induce CD4^+^ T cell responses in vivo. For SARS-CoV-2, an HLA-DR-restricted peptide cocktail vaccine from Tubingen University, Germany was enrolled in a phase I clinical trial (NCT04546841). However, few 9- or 10-mer CD8^+^ T cell epitope peptides have been directly used in vivo as peptide vaccines. To the best of our knowledge, this study is the first to provide experimental evidence that human MHC class I molecule-restricted short peptide cocktail vaccines can induce robust SARS-CoV-2-specific CD8^+^ T cell responses in vivo.

The use of patient PBMCs could only test whether the candidate peptide can be recognized by memory T cells ex vivo, while using healthy donor PBMCs could only test whether the candidate peptides can elicit naive T cells in vitro. Whether the candidate peptide is also able to activate naive T cells in vivo is a better criterion to select an ideal vaccine candidate peptide. Therefore, to further confirm the in vivo immunogenicity of these validated epitopes, 31 epitope peptides restricted by HLA-A0201 were used as representatives to elicit epitope-specific CD8^+^ T cell responses in HLA-A0201/DR1 transgenic mice. Compared with the HLA-A0201 transgenic mice (HHD mice) generally used when identifying HLA-A0201-restricted epitopes and evaluating peptide vaccines [42–44], the HLA-A0201^+/+^/DR1^+/+^/H-2-β_2_m^–/–^/IAβ^–/–^ C57BL/6 mice used in this study should be more suitable to mimic in-human antigen processing and presentation since the interference caused by the presentation of mouse H-2 molecules was weakened. Thus far, due to the lack of HLA and human ACE2 double-transgenic mice, no virus-infected mouse model has been able to reflect the potential T cell responses during natural human infection.

In summary, 120 CD8^+^ T cell epitopes derived from the E, M, N, S, and RdRp proteins of SARS-CoV-2 and restricted by a series of high-frequency HLA-A allotypes were preliminarily identified and validated. Thirty-one HLA-A2-restricted epitopes were generated as short peptide cocktail vaccines and found to trigger robust CD8^+^ T cell responses in HLA-A2/DR1 transgenic mice.

## Materials and methods

### Ethical approval, PBMC preparation, and HLA-A genotyping

Unexposed healthy donor blood samples were collected from the Blood Component Preparation Section of Jiangsu Province Blood Center in the form of white blood cell filter trays after red blood cell filtering. In this instance, informed consent was waived because the white blood cell filter trays were biological specimens obtained from past clinical diagnoses and treatment, but consent was obtained from Jiangsu Province Blood Center. Human sample collection and use were approved by the Clinical Ethics Committee of Affiliated Zhongda Hospital of Southeast University.

White blood cells were collected from the white blood cell filter tray, and PBMCs were instantly isolated by density-gradient centrifugation using Ficoll-Paque. Fresh PBMCs were either used directly or cryopreserved at –80 °C until further testing. HLA-A alleles were identified using PCR sequencing-based typing. Previously described primers [45] were synthesized by Sangon Biotech Co., Ltd. (Shanghai) and are displayed in Supplementary Table S7. The DNA from exon 1 to exon 3 of HLA-A was amplified by PCR using the primer combination A1/A3 followed by sequencing using the primer combination A2F/A2R for exon 2 and A3F/A3R for exon 3. The sequencing data were aligned with the sequences in the HLA database and analyzed using Lasergene software.

### Mice

Female HLA-A*02:01/DR1 transgenic and H-2-β_2_m^–/–^/IAβ^–/–^ C57BL/6 mice aged 10 weeks were generous gifts from the Academy of Military Medical Sciences. Female C57BL/6 mice (10 weeks of age) were purchased from the Comparative Medicine Center of Yangzhou University (Yangzhou, China). Mice were maintained at the specific pathogen-free Animal Centre of Southeast University (Nanjing, China). Animal welfare and experimental procedures were performed in accordance with the Guide for the Care and Use of Laboratory Animals (Ministry of Science and Technology of China, 2006) and were approved by the Animal Ethics Committee of Southeast University.

### In silico prediction of T cell epitopes and peptide synthesis

T cell epitopes spanning the E, M, N, S, and RdRP proteins of SARS-CoV-2 (Wuhan strain) and presented by different HLA-A allotypes were predicted in silico using five epitope predication tools and seven algorithms (IEDB-ANN, IEDB-SMM, SYFPEITHI, EPIJEN, NetMHC, and ConvMHC). For each HLA-A molecule and for each protein, 1–20 9-mer or 10-mer peptides with the highest scores (highest affinity) as predicted by at least two tools were selected as candidate epitopes for further validation. The peptides were synthesized by China Peptides Co., Ltd. (Suzhou) and each had a purity above 95% as determined by HPLC and mass spectrometry. Lyophilized peptides were reconstituted to generate stock solutions at concentrations of 2 mg/mL in a DMSO-PBS solution for use in cellular functional experiments.

### DC-peptide-PBL cocultures

Fresh PBMCs were suspended in serum-free RPMI 1640 and were allowed to adhere to the culture flasks for 2 h in 5% CO_2_ at 37 °C. Nonadherent cells (PBLs) were then removed and cryopreserved at –80 °C until further use. The resulting adherent cells were cultured in RPMI 1640 with 10% FCS, 1% penicillin/streptomycin, recombinant human GM-CSF (rhGM-CSF, 1000 IU/mL, PeproTech), and recombinant human IL-4 (rhIL-4, 500 IU/mL, PeproTech). On days 3 and 5, half of the medium was replaced with fresh complete medium containing the cytokines at the same final concentration detailed above. On day 5, LPS (1 μg/mL, Sigma) was added to induce mDCs. On day 7, the mDCs were collected and identified by flow cytometry (FACSCalibur, BD Bioscience) with FITC-labeled anti-CD83, anti-CD80, anti-CD86, and anti-HLA-DR and PE-labeled anti-HLA-ABC and anti-CD1a. mDCs were incubated with a single peptide (20 μg/mL, corresponding to the HLA-A allele of the indicated healthy donor) in serum-free RPMI 1640 in a 48-well plate (5 × 10^4^ cells/well) for 4 h in 5% CO_2_ at 37 °C, and then PBLs from the same donor (recovered 1 day prior and prelabeled with or without CFSE) were added to the well (1 × 10^6^ cells/well) for an additional 14 days of coculture. Recombinant human IL-2 (20 IU/mL) was added at day 11. On day 14, the corresponding peptide (20 μg/mL) was added. At day 17, rhIL-2 was added again (10 IU/mL). On day 21, the cells were harvested and subjected to ICS or T cell proliferation assays.

### Intracellular IFN-γ staining of stimulated T cells

Cells from DC-peptide-PBL cocultures were harvested and coincubated with the indicated peptide (20 μg/mL) or without peptide (negative control) for 16 h in serum-free RPMI 1640 medium in a 48-well plate at 37 °C and 5% CO_2_. Next, a BFA/monensin mixture was added to the cells for another 6 h of culture. Cells were then harvested, washed, blocked with human FcR Blocking Reagent (MACS Biotech) for 20 min at 4 °C, and stained with FITC-labeled anti-CD3 and APC-labeled anti-CD8 antibodies for 30 min at 4 °C. After washing, the cells were fixed, permeabilized, and further incubated with PE-conjugated anti-human IFN-γ (clone 4S.B3, BD) for another 30 min at 4 °C followed by flow cytometry analysis. The frequencies of IFN-γ^+^ cells in CD3^+^/CD8^+^ populations were calculated.

### CD8^+^ T cell proliferation assay

In the DC-peptide-PBL coculture, PBLs were prestained with CFSE. Briefly, PBLs were thawed, washed, and labeled with CFSE at a final concentration of 1.5 μM for 20 min at 37 °C. After washing, the CFSE-prelabeled PBLs were seeded into the DC-peptide-PBL coculture wells and incubated for 14 days. On day 22, cells were harvested and blocked with human FcR Blocking Reagent (MACS Biotech) for 20 min and then stained with PE-labeled anti-CD3 and APC-labeled anti-CD8 antibodies for 30 min for further analysis by flow cytometry. The proliferation percentage of CD8^+^ T cells in the CD3^+^/CD8^+^ population was analyzed according to the reduction in CFSE staining intensity.

### Generation of HMy2.CIR cell lines expressing the indicated HLA-A molecule

The HMy2.CIR cell line was purchased (Zhongqiao Xinzhou Biotech, Shanghai) and maintained in complete IMDM with 10% FCS and 1% penicillin/streptomycin. Total mRNA was extracted from the PBMCs of the healthy donor with the indicated HLA-A alleles, and the cDNA of each HLA-A allele was amplified using PCR followed by routine construction of the pcDNATM3.1/myc-His(-)A recombinant plasmid. After electrotransfection, the cell lines stably expressing the indicated HLA-A molecule were screened by G418. Then, the cell lines were stained with PE-anti-HLA-ABC (clone W6/32, eBioscience), FITC-anti-HLA-A24 (clone 17A10, MBL), or PE-anti-HLA-A2.1 (clone BB7.2, BD Bioscience). High-expressing cells were then sorted using a fluorescence-activated cell sorter (FACS, BD FACSAria II SORP), followed by pure culture and gene sequencing analyses.

### Competitive peptide binding assay

A set of plasmid-transfected HMy2.CIR cell lines expressing the indicated HLA-A molecule was generated in-house and sorted by flow cytometry. The cell lines were then used in the competitive peptide binding assay according to the references [37]. Briefly, CIR cell lines expressing the indicated HLA-A molecule were washed with acid buffer (0.131 M citric acid and 0.061 M sodium phosphate Na_2_HPO_4_, pH 3.3, 0.22 μm filtered) for 1 min and then neutralized by IMDM containing 0.5% BSA. Cells were washed and seeded into 96-well U culture plate (1 × 10^5^ cells/100 μL/well) with β_2_-m (1 μg/mL). Then, 25 μL of the unlabeled peptide to be tested (5 or 15 μM) and 25 μL of the fluorescently labeled reference peptide (300 nM) was added to the well for 24 h of coincubation at 4 °C. The reference peptides used in this research were FLPSDK(FITC)FPSV (for HLA-A0201, A0203, and A0206) [46], YVNVNK(FITC)GLK (for HLA-A1101 and A3303) [47], EYLVSK(FITC)GVW (for A2402) [48], YLEPAK(FITC)AKY (for A0101) [46], and ASRELK(FITC)VSY (for A3001) (identified in-house). The plate was centrifuged at 600 rpm for 5 min at room temperature (RT). Cells were washed twice with 100 μL of cold 0.5% BSA-PBS. Finally, the cells were resuspended in 150 μL of PBS, transferred to a flow tube, and further analyzed by flow cytometry. Sample % is the percent of FITC^+^ cells in the experimental well, the background % is the percent of FITC^+^ cells in the negative control well, and the max % is the percent of FITC^+^ cells in the positive control well. Competitive binding (%) = [1 – (sample % – background %) / (max % – background %)] × 100%. The IC50 is the concentration of unlabeled competitor peptide required to inhibit the binding of the FITC-labeled reference peptide by 50%, which was calculated from the competitive binding inhibition (%) of the sample at 5 and 15 μM. The binding affinity of each unlabeled peptide with the indicated HLA-A molecule was assessed according to the IC50 value. IC50 < 5 μM (5 μM inhibition >50%) means high binding affinity, 5 μM < IC50 < 15 μM (5 μM inhibition <50% but 15 μM inhibition > 50%) means intermediate binding affinity, IC50 > 15 μM means low or no binding affinity (5 μM inhibition 20–50% or 15 μM inhibition 30–50% means low binding affinity; 5 μM inhibition < 20% or 15 μM inhibition < 30% means no binding affinity).

### T2 cell binding and HLA-A2 molecule stability assay

The HLA-A0201-expressing and TAP-1-deficient human T cell line (T2 cells, Fudancell Biotech, China) was used in this experiment. To assess the affinities of HLA-A2-restricted epitope peptides for the HLA-A0201 molecule, peptide-induced upregulation of HLA-A0201 molecules on T2 cells was measured. Briefly, T2 cells were incubated with a single peptide from one of the 31 epitopes (50 μg/mL), the CMVpp65_495–503_ peptide (NLVPMVATV, 50 μg/mL, as a positive control), the OVA_257–264_ peptide (SIINFKEL, 50 μg/mL, as a negative control), or no peptide and 3 μg/mL β2-m for 16 h at 37 °C and 5% CO_2_. Then, the T2 cells were stained with PE-labeled anti-HLA-A2.1 antibody for 30 min at 4 °C followed by flow cytometry analysis. The fluorescence index (FI) was calculated as follows: FI = (mean PE fluorescence with the given peptide – mean PE fluorescence without peptide) / (mean PE without peptide). FI > 1.0 was the criterion for peptides with high affinity, while peptides with 0.5 <  FI ≤ 1.0 were regarded as intermediate affinity epitopes. Peptides with 0.3 ≤ FI ≤ 0.5 were low-affinity epitopes, while those with FI < 0.3 showed no binding.

### Preparation of peptide pools for vaccine immunization

Thirty-one VEPs restricted by HLA-A0201 molecules were reconstituted in the ideal solution before use at a final concentration of 5 mg/mL for vaccination and 2 mg/mL for T cell response detection. For vaccine immunization, the 31 VEPs (9- or 10-mer) were grouped into four pools (pools v1–v4); for the IFN-γ ELISPOT assay, the VEPs were grouped into eight pools according to their derived protein and acidity and alkalinity; for IFN-γ ICS and ELISA, the 31 VEPs were grouped into five pools according to their derived proteins (Supplementary Table S5).

### Preparation of the PLGA-NPs/peptides vaccine

Peptide-encapsulated PLGA-NPs were freshly prepared using the double-emulsion solvent evaporation method. To generate a PLGA-NPs/peptides vaccine with an amount of peptide equivalent to those of the poly I:C/peptides vaccine and R848/peptides vaccine, the loading efficiency of the PLGA-NPs was calculated before vaccination, and then the PLGA-NPs carrying a single peptide pool were prepared for future injections (one injection/mouse, three mice). Briefly, 60 mg of PLGA with or without a single peptide pool (575 μg/pool, 72–82 μg/peptide) was dissolved in 15 mL of dichloromethane followed by ultrasonic dispersion for 30 s at 40% amplitude to obtain the primary emulsion. Then, the primary emulsion was added to 150 mL of 1% polyvinyl alcohol and sonicated for another 90 s to form the secondary emulsion. The resulting emulsion was added dropwise to 300 mL of 0.5% PVA solution with incessant magnetic stirring to allow dichloromethane evaporation. Four hours later, the solution was collected and centrifuged at 6000 rpm for 5 min. The supernatant was harvested and ultracentrifuged twice at 12,000 rpm for 10 min. The resulting PLGA-NPs were dispersed in deionized water and further mixed with an EDC and NHS solution for 1 h to allow surface activation of the –COOH groups. After washing, this solution was added dropwise to 1% PEI with a magnetic stirring for 4 h at RT. Then, the PEI-conjugated NPs were collected and coincubated with a single peptide pool (575 μg/pool, 72–82 μg/peptide) in sterile PBS overnight at 4 °C on a rotator. Finally, the peptide-encapsulated and peptide-surface coupled PLGA-NPs/peptides vaccine was collected and preserved at 4 °C for further use.

### Preparation of poly I:C/peptides and R848/peptides vaccines and mouse immunization

On day 0, mice were subcutaneously injected as the primary immunization. After that, booster immunizations were administered on days 7 and 21. One day 28, mice were sacrificed for further study. The amount of each peptide administered during each inoculation was 10 μg/mouse per time point, so the amount of each peptide pool was 70 or 80 μg/mouse/time point. Each mouse was inoculated with four peptide pools per time point. Each peptide pool was administered at one injection site (subcutaneously at the tail root, back of the neck, and around the groin). Twelve female HLA-A2/DR1 transgenic mice were randomly divided into four groups. The immunization groups, vaccine formulas, and vaccination schemes are described in Supplementary Table S6.

### ELISPOT and ICS

The 96-well PVDF membrane microplates (Merck & Millipore) were coated with an anti-IFN-γ capture monoclonal antibody (BD) at 4 °C overnight, washed, and blocked. Spleen cells (2 × 10^5^/100 μL) from primed mice were added to each well together with a single peptide pool (2 μg/well for each peptide), PHA (10 μg/mL as a positive control), an irrelevant epitope peptide (HLA-A2-restricted AFP_158–166_ or A24-restricted AFP_424–432_, 2 μg/well for each peptide as a nonspecific control), or no peptide (negative control). After incubation for 20 h at 37 °C and 5% CO_2_, the plates were washed and incubated with a biotinylated anti-IFN-γ detecting antibody (BD) for 2 h at RT. The plates were washed and then incubated with streptavidin-conjugated HRP (BD) for 1 h at RT. After washing the plates, AEC solution (BD) was used as the color developing agent, and the developed spots were imaged and enumerated with a Mabtech IRISTM ELISPOT & FluoroSpot Reader (Mabtech, Swedish).

In addition, spleen cells from primed mice were incubated with a single peptide pool (20 μg/mL for each peptide), PHA (10 μg/mL), irrelevant epitope peptides (AFP_158–166_ and AFP_424–432_, 20 μg/mL for each peptide), or no peptide for 16 h in serum-free RPMI 1640 medium in a 48-well plate at 37 °C and 5% CO_2_. After that, a BFA/monensin mixture was added to the cells for another 6 h of culture. Cells were then harvested, washed, blocked with anti-mouse CD16/CD32 for 20 min at 4 °C, and stained with FITC-labeled anti-CD3 and PE-labeled anti-CD8 antibodies for 30 min at 4 °C. After washing, the cells were fixed and permeabilized following the protocol and further incubated with APC-anti-mouse IFN-γ (clone XMG1.2, BD) for another 30 min at 4 °C followed by flow cytometry. The frequencies of IFN-γ^+^ cells in the CD3^+^/CD8^+^ populations were calculated.

### ELISA

Spleen cells were incubated with a single peptide pool (16 μg/mL for each peptide) or no peptide (negative control) in a 48-well plate for 3 days at 37 °C and 5% CO_2_. Then, the supernatants were collected for ELISA. A mouse IFN-γ detection ELISA kit (Dakewe, China) was used to quantify the amount of IFN-γ in the supernatants according to the manufacturer’s protocol.

### Hematoxylin-eosin staining

Twenty-eight days after primary immunization, the heart, liver, lung, and kidney from each sacrificed mouse were immersed in 4% paraformaldehyde overnight. After that, the individual lobes of organ biopsy material were placed in processing cassettes, dehydrated with a serial alcohol gradient, and embedded in paraffin wax blocks. Five-micrometer-thick tissue sections were dewaxed in xylene, rehydrated through decreasing concentrations of ethanol, and washed in PBS. Then, routine hematoxylin and eosin staining was carried out.

## Supplementary information

Supplementary Tables and Figures
