# Supplementary material for: Screening HLA-A-restricted T cell epitopes of SARS-CoV-2 and the induction of CD8+ T cell responses in HLA-A transgenic mice
Source: Cell Mol Immunol. 2021 Nov 2;18(12):2588–608. doi: 10.1038/s41423-021-00784-8 (PMC8561351; doi:10.1038/s41423-021-00784-8)
Supplement: Supplementary file 1 — Supplementary Tables and Figures [file 41423_2021_784_MOESM1_ESM.pdf]

Supplementary Tables and Figures for manuscript "Screening of HLA-A restricted T cell epitopes of SARS-CoV-2 and induction of CD8^{+} T cell responses in HLA-A transgenic mice ", authored by Xiaoxiao Jin, et al.

Table S1 270 T cell epitopes of SARS-CoV-2 *in silico* predicted were synthesized for further validation.

Protein	Epitope	Predicted HLA-A allele	Epitope sequence	Start Position	End position
E protein	A1	HLA-A*02:01	FLAFVVFLL	20	28
	A2	HLA-A*02:01	SLVKPSFYV	50	58
	A3	HLA-A*02:01	VLLFLAFVV	17	25
	A4	HLA-A*02:01	FLLVTLAIL	26	34
	A5	HLA-A*02:01	RLCAYCCNIV	38	47
	A6	HLA-A*02:07	SVLLFLAFV	16	24
	A7	HLA-A*02:07	LFLAFVVFLL	19	28
	A8	HLA-A*02:06	FVVFLLVTL	23	31
	A9	HLA-A*02:06	LIVNSVLLFL	12	21
	A10	HLA-A*02:03	FLAFVVFLLV	20	29
	A11	HLA-A*02:03	FVSEETGTL	4	12
	A12	HLA-A*11:01	NIVNVSLVK	45	53
	A13	HLA-A*11:01	VTLAILTALR	29	38
	A14	HLA-A*11:01	SLVKPSFYVY	50	59
	A15	HLA-A*11:01	SFYVYSRVK	55	63
	A16	HLA-A*11:01	TLAILTALR	30	38
	A17	HLA-A*11:02	LVKPSFYVY	51	59
	A18	HLA-A*11:02	RVKNLNSSR	61	69
	A19	HLA-A*11:02	VSLVKPSFY	49	57
	A20	HLA-A*24:02	VFLLVTLAI	25	33
	A21	HLA-A*24:02	VLLFLAFVVF	17	26
	A22	HLA-A*24:02	TLIVNSVLLF	11	20
	A23	HLA-A*24:02	VNSVLLFLAF	14	23
	A24	HLA-A*33:03	KPSFYVYSR	53	61
	A25	HLA-A*30:01	SSRVPDLLV	67	75
	A26	HLA-A*30:01	IVNSVLLFL	13	21
M protein	B1	HLA-A*02:01	GLMWLSYFI	89	97
	B2	HLA-A*02:01	KLLEQWNLV	15	23
	B3	HLA-A*02:01	FVLAAVYRI	65	73
	B4	HLA-A*02:01	FLFLTWICLL	26	35
	B5	HLA-A*02:01	LIFLWLLWPV	51	60

	B6	HLA-A*02:01	TLACFVLAAV	61	70
	B7	HLA-A*02:01	FLWLLWPVTL	53	62
	B8	HLA-A*02:07	SMWSFNPET	108	116
	B9	HLA-A*02:07	LLWPVTLAC	56	64
	B10	HLA-A*02:07	FLYIIKLIFL	45	54
	B11	HLA-A*02:06	IAMACLVGL	82	90
	B12	HLA-A*02:06	VTLACFVLA	60	68
	B13	HLA-A*02:06	LVIGFLFLT	22	30
	B14	HLA-A*02:06	YIIKLIFLWL	47	56
	B15	HLA-A*02:03	FIASFRLFA	96	104
	B16	HLA-A*02:03	ILRGHLRIA	144	152
	B17	HLA-A*02:03	AMACLVGLM	83	91
	B18	HLA-A*11:01	ATSRTLSYYK	171	180
	B19	HLA-A*11:01	GTITVEELK	6	14
	B20	HLA-A*11:01	LSYFIASFR	93	101
	B21	HLA-A*11:01	YSRYRIGNYK	196	205
	B22	HLA-A*11:01	AVILRGHLR	142	150
	B23	HLA-A*11:01	LVIGAVILR	138	146
	B24	HLA-A*11:01	FIASFRLFAR	96	105
	B25	HLA-A*11:02	RIAGHHLGR	150	158
	B26	HLA-A*24:02	SYFIASFRLF	94	103
	B27	HLA-A*24:02	MWLSYFIASF	91	100
	B28	HLA-A*24:02	LYIIKLIFLW	46	55
	B29	HLA-A*24:02	QWNLVIGFLF	19	28
	B30	HLA-A*24:02	LWPVTLACF	57	65
	B31	HLA-A*24:02	RFLYIIKLIF	44	53
	B32	HLA-A*33:03	IASFRLFAR	97	105
	B33	HLA-A*33:03	QFAYANRNR	36	44
	B34	HLA-A*33:03	SFRLFARTR	99	107
	B35	HLA-A*33:03	YYKLGASQR	178	186
	B36	HLA-A*30:01	RTRSMWSFN	105	113
	B37	HLA-A*30:01	RNRFLYIIK	42	50
	B38	HLA-A*30:01	TSRTLSYYK	172	180
	B39	HLA-A*30:01	ANRNRFLYI	40	48
	B40	HLA-A*30:01	RYRIGNYKL	198	206
	B41	HLA-A*30:01	HLRIAGHHL	148	156
	B42	HLA-A*30:01	RVAGDSGFA	186	194
N protein	C1	HLA-A*02:01	LLLDRLNQL	222	230
	C2	HLA-A*02:01	GMSRIGMEV	316	324
	C3	HLA-A*02:01	WLTYTGAIKL	330	339
	C4	HLA-A*02:01	YLGTGPEAGL	112	121
	C5	HLA-A*02:01	ALALLLLDRL	218	227
	C6	HLA-A*02:01	IIWVATEGA	130	138

	C7	HLA-A*02:01	ILLNKHIDA	351	359
	C8	HLA-A*02:07	DLDDFSKQL	399	407
	C9	HLA-A*02:07	KLDDKDPNF	338	346
	C10	HLA-A*02:06	QTVTLLPAA	390	398
	C11	HLA-A*02:06	LQLPQGTTL	159	167
	C12	HLA-A*02:06	NTASWFTAL	48	56
	C13	HLA-A*02:06	TTLPKGFYA	165	173
	C14	HLA-A*02:06	RTATKAYNV	262	270
	C15	HLA-A*02:06	QIAQFAPSA	303	311
	C16	HLA-A*02:06	LALLLLDRL	219	227
	C17	HLA-A*02:03	QLQQSMSSA	406	414
	C18	HLA-A*02:03	RMAGNGGDA	209	217
	C19	HLA-A*02:03	SAFFGMSRI	312	320
	C20	HLA-A*11:01	ASAFFGMSR	311	319
	C21	HLA-A*11:01	ATEGALNTPK	134	143
	C22	HLA-A*11:01	KSAAEASKK	249	257
	C23	HLA-A*11:01	FTALTQHGK	53	61
	C24	HLA-A*11:01	QLPQGTTLPK	160	169
	C25	HLA-A*11:01	LLNKHIDAYK	352	361
	C26	HLA-A*11:01	AGLPYGANK	119	127
	C27	HLA-A*11:01	QQQGQTVTK	240	248
	C28	HLA-A*11:01	VTPSGTWLTY	324	333
	C29	HLA-A*11:02	KTFPPTEPK	361	369
	C30	HLA-A*24:02	YYRRATRRI	86	94
	C31	HLA-A*24:02	QFAPSASAFF	306	315
	C32	HLA-A*24:02	KHIDAYKTF	355	363
	C33	HLA-A*24:02	TWLTYTGAI	329	337
	C34	HLA-A*24:02	GYYRRATRRI	85	94
	C35	HLA-A*24:02	LSPRWYFYYL	104	113
	C36	HLA-A*30:01	RSRNSSRNS	189	197
	C37	HLA-A*30:01	GTRNPANNA	147	155
	C38	HLA-A*30:01	SSRGTSPAR	201	209
	C39	HLA-A*30:01	RSKQRRPQG	36	44
	C40	HLA-A*30:01	LIRQGTDYK	291	299
	C41	HLA-A*30:01	SSRNSTPGS	193	201
	C42	HLA-A*30:01	SSRSSSRSR	183	191
	C43	HLA-A*30:01	RQKRTATKA	259	267
	C44	HLA-A*30:01	SSRSRNSSR	187	195
	C45	HLA-A*33:03	NVTQAFGRR	269	277
	C46	HLA-A*33:03	IGYYRRATR	84	92
	C47	HLA-A*33:03	NTPKDHIGTR	140	149
	C48	HLA-A*33:03	QASSRSSSR	181	189
	C49	HLA-A*11:02	YKTFPPTEPK	360	369

S protein	D1	HLA-A*02:01	YLQPRTFLL	269	277
	D2	HLA-A*02:01	FIAGLIAIV	1220	1228
	D3	HLA-A*02:01	ELLHAPATV	516	524
	D4	HLA-A*02:01	SIIAYTMSL	691	699
	D5	HLA-A*02:01	KLNDLCFTNV	386	395
	D6	HLA-A*02:01	RLDKVEAEV	983	991
	D7	HLA-A*02:01	VLNDILSRL	976	984
	D8	HLA-A*02:01	FTISVTTEI	718	726
	D9	HLA-A*02:01	LLFNKVTLA	821	829
	D10	HLA-A*02:01	KIADYNYKL	417	425
	D11	HLA-A*02:01	VVFLHVTYV	1060	1068
	D12	HLA-A*02:01	FVFLVLLPLV	2	11
	D13	HLA-A*02:01	MIAQYTSAL	869	877
	D14	HLA-A*02:01	GLIAIVMVTI	1223	1232
	D15	HLA-A*02:01	SVTTEILPV	721	729
	D16	HLA-A*02:07	KLPDDFTGCV	424	433
	D17	HLA-A*02:07	RLQSLQTYV	1000	1008
	D18	HLA-A*02:07	LLPLVSSQCV	7	16
	D19	HLA-A*02:06	AVDCALDPL	288	296
	D20	HLA-A*02:06	KQLSSNFGA	964	972
	D21	HLA-A*02:06	FQFCNDPFL	133	141
	D22	HLA-A*02:06	MQMAYRFNGI	900	909
	D23	HLA-A*02:06	YQDVNCTEV	612	620
	D24	HLA-A*02:06	KQIYKTPPI	786	794
	D25	HLA-A*02:06	LQIPFAMQM	894	902
	D26	HLA-A*02:06	LQSYGFQPT	492	500
	D27	HLA-A*02:06	NTQEVFAQV	777	785
	D28	HLA-A*02:06	TQLNRALTGI	761	770
	D29	HLA-A*02:03	HLMSFPQSA	1048	1056
	D30	HLA-A*02:03	FLHVTYVPA	1062	1070
	D31	HLA-A*02:03	QLNRALTGI	762	770
	D32	HLA-A*02:03	FKIYSKHTPI	201	210
	D33	HLA-A*02:03	FVSNGTHWFV	1095	1104
	D34	HLA-A*11:01	GVYFASTEK	89	97
	D35	HLA-A*11:01	MTSCCSCLK	1237	1245
	D36	HLA-A*11:01	RLFRKSNLK	454	462
	D37	HLA-A*11:01	SSTASALGK	939	947
	D38	HLA-A*11:01	NSASFSTFK	370	378
	D39	HLA-A*11:01	VTLADAGFIK	826	835
	D40	HLA-A*11:01	CTLKSFTVEK	301	310
	D41	HLA-A*11:01	SLIDLQELGK	1196	1205
	D42	HLA-A*11:01	FIEDLLFNK	817	825
	D43	HLA-A*11:01	EILPVSMTK	725	733

	D44	HLA-A*11:01	AQALNTLVK	956	964
	D45	HLA-A*11:01	QIYKTPPIK	787	795
	D46	HLA-A*11:02	VTYVPAQEK	1065	1073
	D47	HLA-A*11:02	ASANLAATK	1020	1028
	D48	HLA-A*11:02	GTHWFVTQR	1099	1107
	D49	HLA-A*11:02	GVLTESNKK	550	558
	D50	HLA-A*11:02	GVYYHKNNK	142	150
	D51	HLA-A*24:02	QYIKWPWYI	1208	1216
	D52	HLA-A*24:02	VYAWNRKRI	350	358
	D53	HLA-A*24:02	NYNYLYRLF	448	456
	D54	HLA-A*24:02	VYSTGSNVF	635	643
	D55	HLA-A*24:02	PYRVVVLSF	507	515
	D56	HLA-A*24:02	LYNSASFSTF	368	377
	D57	HLA-A*24:02	VYSSANNCTF	159	168
	D58	HLA-A*24:02	CYFPLQSYGF	488	497
	D59	HLA-A*24:02	PFAMQMAYRF	897	906
	D60	HLA-A*24:02	TYVPAQEKNF	1066	1075
	D61	HLA-A*24:02	IYSKHTPINL	203	212
	D62	HLA-A*24:02	IYKTPPIKDF	788	797
	D63	HLA-A*24:02	TFEYVSQPF	167	175
	D64	HLA-A*24:02	CFTNVYADSF	391	400
	D65	HLA-A*30:01	ATRFASVYA	344	352
	D66	HLA-A*30:01	ITRFQTLLA	235	243
	D67	HLA-A*30:01	TTRTQLPPA	19	27
	D68	HLA-A*30:01	KCYGVSPTK	378	386
	D69	HLA-A*30:01	AYRFNGIGV	903	911
	D70	HLA-A*30:01	RKRISNCVA	355	363
	D71	HLA-A*30:01	KNLREFVFK	187	195
	D72	HLA-A*30:01	ASVYAWNRK	348	356
	D73	HLA-A*30:01	RARSVASQSI	683	692
	D74	HLA-A*30:01	GTKRFDNPV	75	83
	D75	HLA-A*30:01	HVSGTNGTK	69	77
	D76	HLA-A*33:03	SVYAWNRKR	349	357
	D77	HLA-A*33:03	VYYPDKVFR	36	44
	D78	HLA-A*33:03	QTNSPRRAR	677	685
	D79	HLA-A*33:03	NVYADSFVIR	394	403
	D80	HLA-A*33:03	YYVGYLQPR	265	273
	D81	HLA-A*33:03	GIYQTSNFR	311	319
	D82	HLA-A*33:03	NGVGYQPYR	501	509
	D83	HLA-A*33:03	STGSNVFQTR	637	646
RdRp protein	R1	HLA-A*02:01	LLMPILTLT	240	248
	R2	HLA-A*02:01	TMADLVYAL	123	131
	R3	HLA-A*02:01	LMIERFVSL	854	862

	R4	HLA-A*02:01	AMRNAGIVGV	195	204
	R5	HLA-A*02:01	SLAIDAYPL	861	869
	R6	HLA-A*02:01	NLLKDCPAV	88	96
	R7	HLA-A*02:01	NLIDSYFVV	64	72
	R8	HLA-A*02:01	FVNEFYAYL	741	749
	R9	HLA-A*02:01	ILHCANFNV	307	315
	R10	HLA-A*02:01	KIFVDGVPFV	332	341
	R11	HLA-A*02:01	VMCGGSLYV	667	675
	R12	HLA-A*02:01	MLDMYSVML	899	907
	R13	HLA-A*02:01	NMLRIMASL	628	636
	R14	HLA-A*02:01	RLANECAQV	654	662
	R15	HLA-A*02:01	QLLFVVEVV	468	476
	R16	HLA-A*02:07	FVDGVPFVV	334	342
	R17	HLA-A*02:07	YLPYPDPSRI	828	837
	R18	HLA-A*02:07	FPPTSFGPLV	321	330
	R19	HLA-A*02:06	RQLLFVVEV	467	475
	R20	HLA-A*02:06	RILGAGCFV	836	844
	R21	HLA-A*02:06	SVAALTNNV	397	405
	R22	HLA-A*02:06	MILSDDAVV	756	764
	R23	HLA-A*02:06	LSFKELLVYA	366	375
	R24	HLA-A*02:03	MLKTVYSDV	601	609
	R25	HLA-A*02:03	MLRIMASLV	629	637
	R26	HLA-A*02:03	SLSHRFYRL	647	655
	R27	HLA-A*02:03	AMYTPHTVL	923	931
	R28	HLA-A*02:03	SIAATRGATV	578	587
	R29	HLA-A*02:03	LLSTDGNKI	707	715
	R30	HLA-A*11:01	ASGNLLLDK	383	391
	R31	HLA-A*11:01	TSFGPLVRK	324	332
	R32	HLA-A*11:01	KSAGFPFNK	500	508
	R33	HLA-A*11:01	KVAGFAKFLK	41	50
	R34	HLA-A*11:01	MTNRQFHQK	566	574
	R35	HLA-A*11:01	AVAKHDFFK	95	103
	R36	HLA-A*11:01	AIDAYPLTK	863	871
	R37	HLA-A*11:01	LVASIKNFK	775	783
	R38	HLA-A*11:01	VVSTGYHFR	341	349
	R39	HLA-A*11:01	TVKPGNFNK	409	417
	R40	HLA-A*11:01	KTNCCRFQEK	50	59
	R41	HLA-A*11:01	AISDYDYYR	449	457
	R42	HLA-A*11:01	CSQHTMLVK	813	821
	R43	HLA-A*11:01	CSLSHRFYR	646	654
	R44	HLA-A*11:01	GTSTDVVYR	25	33
	R45	HLA-A*11:02	ATVVIGTSK	585	593
	R46	HLA-A*11:02	KLFDRYFKY	281	289

	R47	HLA-A*24:02	SYYSLLMPI	236	244
	R48	HLA-A*24:02	SYFVVKRHTF	68	77
	R49	HLA-A*24:02	AYANSVFNI	688	696
	R50	HLA-A*24:02	YFNKKDWYDF	156	165
	R51	HLA-A*24:02	FYGGWHNML	594	602
	R52	HLA-A*24:02	IYNDKVAGF	37	45
	R53	HLA-A*24:02	RYNLPTMCDI	457	466
	R54	HLA-A*24:02	KYVRNLQHRL	718	727
	R55	HLA-A*24:02	NFNKDFYDF	414	422
	R56	HLA-A*24:02	EYADVFHLYL	876	885
	R57	HLA-A*24:02	FYAYLRKHF	745	753
	R58	HLA-A*24:02	TYHPNCVNCL	293	302
	R59	HLA-A*24:02	LYLQYIRKL	883	891
	R60	HLA-A*30:01	ATRGATVVI	581	589
	R61	HLA-A*30:01	RLKLFDRYFK	279	288
	R62	HLA-A*30:01	RVRQALLKT	181	189
	R63	HLA-A*30:01	RQFHQKLLK	569	577
	R64	HLA-A*30:01	HISRQRLTK	113	121
	R65	HLA-A*30:01	KARLYYDSM	511	519
	R66	HLA-A*33:03	DFYDFAVSK	418	426
	R67	HLA-A*33:03	MVPHISRQR	110	118
	R68	HLA-A*33:03	LLKSIAATR	575	583
	R69	HLA-A*33:03	DALFAYTKR	525	533
	R70	HLA-A*33:03	RVCGVSAAR	10	18

Table S2: Homologous analyses of 120 SARS-CoV-2 CD8$^+$ T cell epitopes with SARS-CoV, common-cold HCoVs and SARS-CoV-2 variants.

**Protein**	**Epitope**	**SARS-CoV-2**	**SARS-CoV**	**OC43**	**NL63**	**HKU1**	**229E**
E	A1	FLAFVVFLL	FLAFVVFLL	no	no	no	no
E	A3	VLLFLAFVV	VLLFLAFVV	no	no	no	no
E	A4	FLLVTLAIL	FLLVTLAIL	no	no	no	no
E	A5	RLCAYCCNIV	RLCAYCCNIV	no	no	no	no
E	A6	SVLLFLAFV	SVLLFLAFV	no	no	no	no
E	A7	LFLAFVVFLL	LFLAFVVFLL	no	no	no	no
E	A9	LIVNSVLLFL	LIVNSVLLFL	no	no	no	no
E	A10	FLAFVVFLLV	FLAFVVFLLV	no	no	no	no
E	A12	NIVNVSLVK	NIVNVSLVK	no	no	no	no
E	A16	TLAILTALR	TLAILTALR	no	no	no	no
E	A18	RVKNLNSSR	RVKNLNSSE	no	no	no	no
E	A19	VSLVKPSFY	VSLVKPTVY	no	no	no	no
E	A20	VFLLVTLAI	VFLLVTLAI	no	no	no	no
E	A21	VLLFLAFVVF	VLLFLAFVVF	no	no	no	no
E	A22	TLIVNSVLLF	TLIVNSVLLF	no	no	no	no
E	A23	VNSVLLFLAF	VNSVLLFLAF	no	no	no	no
E	A25	SSRVPDLLV	SSEGVPDLLV	no	no	no	no
E	A26	IVNSVLLFL	IVNSVLLFL	no	no	no	no
M	B1	GLMWLSYFI	GLMWLSYFV	no	no	no	no
M	B2	KLLEQWNLV	QLLEQWNLV	no	no	no	no
M	B3	FVLAAVYRI	FVLAAVYRI	no	no	no	no
M	B4	FLFLTWICLL	FLFLAWIMLL	no	no	no	no
M	B6	TLACFVLAAV	TLACFVLAAV	no	no	no	no
M	B10	FLYIIKLIFL	FLYIIKLVFL	no	no	no	no
M	B11	IAMACLVGL	IAMACIVGL	no	no	no	no
M	B12	VTLACFVLA	VTLACFVLA	no	no	no	no
M	B15	FIASFRLFA	FVASFRLFA	no	no	no	no
M	B16	ILRGHLRIA	IIRGHLRMA	no	no	no	no
M	B17	AMACLVGLM	AMACIVGLM	no	no	no	no
M	B18	ATSRTLSYYK	ATSRTLSYYK	no	no	no	no
M	B20	LSYFIASFR	LSYFVASFR	no	no	no	no
M	B21	YSRYRIGNYK	YNRYRIGNYK	no	no	no	no
M	B23	LVIGAVILR	LVIGAVIIR	no	no	no	no
M	B26	SYFIASFRLF	SYFVASFRLF	no	no	no	no
M	B28	LYIIKLIFLW	LYIIKLVFLW	no	no	no	no
M	B29	QWNLVIGFLF	QWNLVIGFLF	no	no	no	no
M	B30	LWPVTLACF	LWPVTLACF	no	no	no	no
M	B31	RFLYIIKLIF	RFLYIIKLVF	no	no	no	no

M	B34	SFRLFARTR	SFRLFARTR	no	no	no	SFRLFRRAR
M	B35	YYKLGASQR	YYKLGASQR	no	no	no	no
M	B36	RTRSMWSFN	RTRSMWSFN	RTGSFWSFN	no	RTGSWWSFN	no
M	B37	RNRFLYIIK	RNRFLYIIK	no	no	no	no
M	B38	ANRNRFLYI	SNRNRFLYI	no	no	no	no
M	B40	RYRIGNYKL	RYRIGNYKL	no	no	no	no
M	B41	HLRIAGHHL	HLRMAGHSL	no	no	no	no
N	C1	LLLDRLNQL	LLLDRLNQL	no	no	no	no
N	C3	WLTYTGAIKL	WLTYHGAIKL	no	no	no	no
N	C10	QTVTLLPAA	PTVTLLPAA	no	no	no	no
N	C12	NTASWFTAL	NTASWFTAL	no	no	no	no
N	C16	LALLLLDRL	LALLLLDRL	no	no	no	no
N	C17	QLQQSMSSA	QLQNSMSGA	no	no	no	no
N	C27	QQQGQTVTK	QQQGQTVTK	no	no	no	no
N	C35	LSPRWYFYYL	LSPRWYFYYL	LLPRWYFYYL	no	LLPRWYFYYL	no
N	C45	NVTQAFGRR	NVTQAFGRR	no	no	no	NVTQCFGPR
N	C46	IGYYRRATR	IGYYRRATR	no	no	no	no
N	C47	NTPKDHIGTR	NTPKDHIGTR	no	no	no	no
N	C49	YKTFPPTEPK	YKTFPPTEPK	no	no	no	no
S	D2	FIAGLIAIV	FIAGLIAIV	no	no	no	no
S	D5	KLNDLCFTNV	KLNDLCFSNV	no	no	no	no
S	D6	RLDKVEAEV	RLDKVEAEV	no	no	no	no
S	D12	FVFLVLLPLV	no	no	no	no	no
S	D13	MIAQYTSAL	MIAAYTAAL	no	no	no	no
S	D17	RLQSLQTYV	RLQSLQTYV	no	no	no	no
S	D26	LQSYGFQPT	no	no	no	no	no
S	D30	FLHVTYVPA	FLHVTYVPS	no	no	no	no
S	D31	QLNRALTGI	QLNRALSGI	no	no	no	no
S	D32	FKIYSKHTPI	no	no	no	no	no
S	D33	FVSNGTHWFV	no	no	no	no	no
S	D34	GVYFASTEK	GIYFAATEK	no	no	no	no
S	D38	NSASFSTFK	NSTFFSTFK	no	no	no	no
S	D40	CTLKSFTVEK	no	no	no	no	no
S	D41	SLIDLQELGK	SLIDLQELGK	no	no	no	no
S	D42	FIEDLLFNK	FIEDLLFNK	AIEDLLFDK	no	FFEDLLFDK	no
S	D46	VTYVPAQEK	VTYVPSQER	no	no	no	no
S	D47	ASANLAATK	ASANLAATK	no	no	no	no
S	D48	GTHWFVTQR	GTSWFITQR	no	no	no	no
S	D50	GVYYHKNNK	no	no	no	no	no
S	D52	VYAWNRKRI	VYAWERKKI	no	no	no	no
S	D53	NYNYLYRLF	no	no	no	no	no
S	D55	PYRVVVLSF	PYRVVVLSF	no	no	no	no
S	D56	LYNSASFSTF	LYNSTFFSTF	no	no	no	no

S	D62	IYKTPPIKDF	no	no	no	no	no
S	D64	CFTNVYADSF	CFSNVYADSF	no	no	no	no
S	D65	ATRFASVYA	ATKFPSVYA	no	no	no	no
S	D71	KNLREFVFK	KHLREFVFK	no	no	no	no
S	D72	ASVYAWNRK	PSVYAWERK	no	no	no	no
S	D76	SVYAWNRKR	SVYAWERKK	no	no	no	no
S	D77	VYYPDKVFR	VYYPDEIFR	no	no	no	no
S	D78	QTNSPRRAR	no	no	no	no	no
S	D79	NVYADSFVIR	NVYADSFVVK	no	no	no	no
S	D80	YYVGYLQPR	no	no	no	no	no
S	D81	GIYQTSNFR	GIYQTSNFR	no	no	no	no
S	D82	NGVGYQPYR	TGIGYQPYR	no	no	no	no
RdRp	R4	AMRNAGIVGV	AMRDAGIVGV	no	no	no	no
RdRp	R5	SLAIDAYPL	SLAIDAYPL	SLAIDAYPL	SLAIDAYPL	SLAIDAYPL	SLAIDAYPL
RdRp	R6	NLLKDCPAV	NLVKDCPAV	no	no	no	NLLKGCNAV
RdRp	R8	FVNEFYAYL	FVDEFYAYL	no	no	no	no
RdRp	R9	ILHCANFNV	ILHCANFNV	IIHCANFNI	no	IIHCANFNI	ILHCSNFNT
RdRp	R10	KIFVDGVPFV	KIFVDGVPFV	QIFVDGVPFV	no	QIFVDGVPFV	no
RdRp	R11	VMCGGSLYV	VMCGGSLYV	VMCGGCYYV	no	VMCGGCYYV	no
RdRp	R12	MLDMYSVML	MLDMYSVML	no	no	no	no
RdRp	R13	NMLRIMASL	NMLRIMASL	no	no	no	no
RdRp	R14	RLANECAQV	RLANECAQV	RLANECAQV	RLGNELAQV	RLANECAQV	RLSNELAQV
RdRp	R15	QLLFVVEVV	QLLFVVEVV	QLLFVLEVV	no	QLLFVLEVV	no
RdRp	R17	YLPYPDPSRI	YLPYPDPSRI	YLPYPNPSRI	YLPYPDPSRI	YLPYPDPSRI	YLPYPDPSRI
RdRp	R23	LSFKELLVYA	LSFKELLVYA	no	no	no	no
RdRp	R24	MLKTVYSDV	MLKTVYSDV	no	no	no	no
RdRp	R30	ASGNLLLDK	ASGNLLLDK	no	no	no	no
RdRp	R32	KSAGFPFNK	KSAGFPFNK	KSAGYPFNK	KSAGWPLNK	KSAGYPFNK	KSAGWPLNK
RdRp	R34	MTNRQFHQK	MTNRQFHQK	MTGRMFHQK	MTTRQYHQK	MTGRMFHQK	MTTRQFHQK
RdRp	R35	AVAKHDFFK	AVAVHDFFK	no	no	no	AVAKHDFFT
RdRp	R38	VVSTGYHFR	VVSTGYHFR	no	no	no	no
RdRp	R39	TVKPGNFNK	TVKPGNFNK	TVKPGNFNQ	no	TVKPGNFNQ	TVKPGHFNK
RdRp	R40	KTNCCRFQEK	KTNCCRFQEK	no	no	no	no
RdRp	R41	AISDYDYYR	AISDYDYYR	no	no	no	AIKDFDYYR
RdRp	R42	CSQHTMLVK	CSQHTMLVK	CSQHTMLVK	no	CSQHTMLVK	no
RdRp	R43	CSLSHRFYR	CNLSHRFYR	CSQSDRFYR	no	no	no
RdRp	R44	GTSTDVVYR	GTSTDVVYR	no	no	no	no
RdRp	R47	SYYSLLMPI	SYYSLLMPI	no	SYYSYMMPI	no	no
RdRp	R48	SYFVVKRHTF	SYFVVKRHTM	no	no	no	no
Total		120	110	15	6	14	12
**Protein**	**Epitope**	**SARS-CoV-2**	**B.1.1.7**	**B.1.351**	**P.1**	**B.1.617**	**Denmark variant**
S	D50	GVYYHKNNK	GVYHKNNK	no	no	no	no

S	D53	NYNYLYRLF	no	no	no	no	NYNYLFRLF
S	D78	no	no	no	no	QTNSRRRAR	no
S	D82	NGVGYQPYR	YGVGYQPYR	YGVGYQPYR	YGVGYQPYR	no	no
**Protein**	**Epitope**	**SARS-CoV-2**	**B.1.617.2 (Delta)**	**C.37 (Lambda)**			
S	D50	GVYYHKNNK	DVYYHKNNK	no			
S	D53	NYNYLYRLF	NYNYRYRLF	NYNYQYRLF			
S	D78	QTNSPRRAR	QTNSRRRAR	no			

Note: "no" means there is no consensus epitope with 0-2 amino acids deviation in the SARS-CoV, indicated HCoVs or SARS-CoV-2 variants.

Table S3: 31 validated epitope peptides restricted by HLA-A0201 molecule

Epitope	Sequence	Predicted HLA-A restriction	Affinity with HLA-A0201 **IEDB<500nM ANN (nM)**	**DC-peptide-PBL co-cultures**		**Peptide competitive binding assay (affinity)**				**T2 binding assay (affinity)**
				**method**	**enhance (fold)**	**A0201**	**A0203**	**A0206**	**A0207**	**A0201**
A1	FLAFVVFLL	A0201, A0207 A0206, A2402	5.26	IFN-γ	2.300	no	ns	ns	inter	inter
A3	VLLFLAFVV	A0201, A0203 A1101, A1102	21.72	IFN-γ	4.142	no	ns	ns	ns	low
A4	FLLVTLAIL	A0201, A0207 A0203	39.95	IFN-γ	5.366	no	no	ns	low	inter
A5	RLCAYCCNIV	A0201, A0203	145.67	IFN-γ	8.288	no	no	ns	ns	high
B1	GLMWLSYFI	A0201, A0206 A0203	3.87	IFN-γ	4.980	high	inter	inter	ns	high
B2	KLLEQWNLV	A0201, A0206 A0203, A0207	7.57	IFN-γ	2.281	inter	high	inter	high	inter
B3	FVLAAVYRI	A0201, A0207 A0206	16.52	IFN-γ	5.073	high	ns	inter	high	high
B4	FLFLTWICLL	A0201, A0206	10.42	IFN-γ	3.166	no	ns	no	ns	inter
B6	TLACFVLAAV	A0201, A0207 A0203	20.28	IFN-γ	3.421	inter	low	ns	high	high
C1	LLLDRLNQL	A0201, A0206 A0203	14.81	CFSE	1.300	no	inter	no	ns	high
C2	GMSRIGMEV	A0201	reported	ns	ns	ns	ns	ns	ns	no
C3	WLTYTGAIKL	A0201, A0206 A0203	284.75	IFN-γ	3.709	no	no	no	ns	low
D2	FIAGLIAIV	A0201, A0206 A0203	10.29	IFN-γ	2.223	inter	high	inter	ns	high
D5	KLNDLCFTNV	A0201, A0207 A0203	15.27	IFN-γ	5.792	low	high	ns	low	high
D6	RLDKVEAEV	A0201, A0207	38.95	IFN-γ	2.069	no	ns	ns	low	high
D7	VLNDILSRL	A0201	reported	ns	ns	ns	ns	ns	ns	inter
D11	VVFLHVTYV	A0201	reported	ns	ns	ns	ns	ns	ns	high
D12	FVFLVLLPLV	A0201, A0207 A0206	32.64	CFSE	1.234	low	ns	no	no	low
D13	MIAQYTSAL	A0201, A0203	41.52	IFN-γ	11.013	no	inter	ns	ns	no
R3	LMIERFVSL	A0201	reported		ns	ns	ns	ns	ns	high
R4	AMRNAGIVGV	A0201	78.14	IFN-γ	2.078	inter	ns	ns	ns	high
R5	SLAIDAYPL	A0201, A0203	20.82	IFN-γ	2.175	high	high	ns	ns	high
R6	NLLKDCPAV	A0201, A0207 A0206, A0203	18.8	CFSE	1.493	no	no	no	low	high
R8	FVNEFYAYL	A0201	8.24	IFN-γ	2.132	high	ns	ns	ns	high
R9	ILHCANFNV	A0201, A0206 A0203	8.64	IFN-γ	2.070	high	high	inter	ns	high
R10	KIFVDGVPFV	A0201	10.65	IFN-γ	2.043	high	ns	ns	ns	inter
R11	VMCGGSLYV	A0201	14.27	IFN-γ	3.036	inter	ns	ns	ns	high
R12	MLDMYSVML	A0201	15.97	IFN-γ	2.886	high	ns	ns	ns	inter
R13	NMLRIMASL	A0201	41.52	IFN-γ	2.470	no	ns	ns	ns	low
R14	RLANECAQV	A0201, A0203	52.91	IFN-γ	2.333	no	inter	ns	ns	high
R15	QLLFVVEVV	A0201	163.5	IFN-γ	3.184	high	ns	ns	ns	high

Note 1: **IFN-γ:** After DC-peptide-PBLs co-cultures, the frequency of IFN-γ$^+$/CD8$^+$ T cells in CD3$^+$/CD8$^+$ cell population was analyzed by flow cytometry. **CFSE:** After DC-peptide-CFSE-prelabeled PBLs co-cultures, the proliferation percentage of CD8$^+$ T cells in CD3$^+$/CD8$^+$ population was analyzed according to the reduction of CFSE-staining brightness. **Enhance (fold):** The folds of frequency of IFN-γ$^+$/CD8$^+$ T cells or proliferation percentage of CD8$^+$ T cells in the DC-peptide-PBL co-culture wells when compared with

that in the DC-PBL co-culture well without peptide. **ns:** no test. Reported: the epitope has been reported previously.

Note 2: Named epitopes beginning with A, B, C, D and R was derived from E, M, N, S and RdRp protein, respectively.

Table S4: Affinity of 31 validated epitope peptides with HLA-A0201 molecules on T2 cells

Protein	Peptide	High affinity (FI>1)	Intermediate affinity (0.5＜FI ≤1)	Low affinity (0.3≤ FI ≤ 0.5)	No affinity FI < 0.3	FI
E	A1		inter			0.65
	A3			low		0.39
	A4		inter			0.82
	A5	high				1.65
	B1	high				2.28
M	B2		inter			0.94
	B3	high				3.34
	B4		inter			0.51
	B6	high				1.73
N	C1	high				1.26
	C2				no	0.21
	C3			low		0.43
S	D2	high				1.12
	D5	high				2.59
	D6	high				3.65
	D7		inter			0.89
	D11	high				2.11
	D12			low		0.35
	D13				no	0.24
RdRp	R3	high				2.91
	R4	high				1.98
	R5	high				4.00
	R6	high				2.48
	R8	high				2.11
	R9	high				3.65
	R10		inter			0.58
	R11	high				1.95
	R12		inter			0.79
	R13			low		0.41
	R14	high				2.21
	R15	high				3.67
Total	31	18	7	4	2	

Note: The fluorescence index (FI) was calculated as follows: FI = (mean PE fluorescence with the given peptide - mean PE fluorescence without peptide)/ (mean PE without peptide). FI > 0.5 was the criteria of peptides with affinity while peptides with FI > 1 were regarded as high-affinity epitopes; 0.3 ≤ FI ≤ 0.5 means low affinity while FI < 0.3 means no binding.

Table S5: 31 epitope peptides were grouped into several pools for vaccine generation and specific T cell detection.

For vaccine generation	pool-v1	A1+A3+A4+A5+B1+B2+B3+B4
	pool-v2	B6+C1+C2+C3+D2+D5+D6+D7
	pool-v3	D11+D12+D13+R3+R4+R5+R6+R8
	pool-v4	R9+R10+R11+R12+R13+R14+R15
For ELISPOT assay	pool-s1	A1+A3+A4+A5
	pool-s2	B1+B2+ B4+B6
	pool-s3	B3
	pool-s4	C1+C2+C3
	pool-s5	D2+D5+D6+D7+ D12+D13
	pool-s6	D11
	pool-s7	R5+R6+R8+R11+R12+R14+R15
	pool-s8	R3+R4+R9+R10+R13
For ICS and ELISA	pool-c1	A1+A3+A4+A5
	Pool-c2	B1+B2+B3+B4+B6
	pool-c3	C1+C2+C3
	pool-c4	D2+D5+D6+D7+D11+D12+D13
	pool-c5	R3+ R4+R5+R6+R8+R9+R10+R11+R12 + R13+R14+R15

Table S6: Immunization groups, vaccines formula and vaccination scheme

PLGA-NPs /peptides (Vaccine A)	pool-v1: PLGA-NPs/pool-v1(60mg/240μg) mixed with 75μL Poly I:C and 225μLNS, injected into 3 mice, 100μL/mouse, one injection site/mouse<br>pool-v2: PLGA-NPs/pool-v2(60mg/240μg) mixed with 75μL Poly I:C and 225μLNS, injected into 3 mice, 100μL/mouse, one injection site/mouse<br>pool-v3: PLGA-NPs/pool-v3(60mg/240μg) mixed with 75μL Poly I:C and 225μLNS, injected into 3 mice, 100μL/mouse, one injection site/mouse<br>pool-v4: PLGA-NPs/pool-v4(60mg/210μg) mixed with 75μL Poly I:C and 225μLNS, injected into 3 mice, 100μL/mouse, one injection site/mouse
R848 /peptides (Vaccine B)	pool-v1:50μL pool-v1 mixed with 75μL R848 and 175μLNS, injected into 3 mice, 100μL/mouse, one injection site/mouse<br>pool-v2:50μL pool-v2 mixed with 75μL R848 and 175μLNS, injected into 3 mice, 100μL/mouse, one injection site/mouse<br>pool-v3:50μL pool-v3 mixed with 75μL R848 and 175μLNS, injected into 3 mice, 100μL/mouse, one injection site/mouse<br>pool-v4:45μL pool-v4 mixed with 75μL R848 and 180μLNS, injected into 3 mice, 100μL/mouse, one injection site/mouse
PolyI:C /peptides (Vaccine C)	pool-v1: 50μL pool-v1mixed with 75μL Poly I: C and 175μLNS, injected into 3 mice, 100μL/mouse, one injection site/mouse<br>pool-v2: 50μL pool-v2mixed with 75μL Poly I: C and 175μLNS, injected into 3 mice, 100μL/mouse, one injection site/mouse<br>pool-v3:50μL pool-v3mixed with 75μL Poly I: C and 175μLNS, injected into 3 mice, 100μL/mouse, one injection site/mouse<br>pool-v4:45μL pool-v4 mixed with 75μL Poly I: C and 180μLNS, injected into 3 mice, 100μL/mouse, one injection site/mouse
Control group	Control mouse 1: 100μL NS/injection site, 4 injection sites<br>Control mouse 2: 80mg empty PLGA-NPs mixed with 400μL NS, 100μL/injection site, 4 injection sites<br>Control mouse 3: 100μL NS/injection site, 4 injection sites

Table S7 Primers used for HLA-A genotyping

Primers	Sequence (5'-3')	Anneal site	Length
A1	GAAACSGCCTCTGYGGGGAGAAGCAA	HLA-A intronl:21-26	985bp
A3	TGTTGGTCCCAATTGTCTCCCCTC	HLA-A intron3:66-89	
A2F	AGCCGCGCCKGGASGAGGGTC	Exon2 intron2: 99-119	270bp
A2R	GGCCCGTCCGTGGGGGATGAG	Exon2 intron2: 37-57	
A3F	GTTTCATTTTGRTTKAGGCCA	Exon3 intron3: 150-171	276bp
A3R	TGTGGGAGGCCAGCCCGGGAGA	Exon3 intron3: 41-66	

Figure S1:

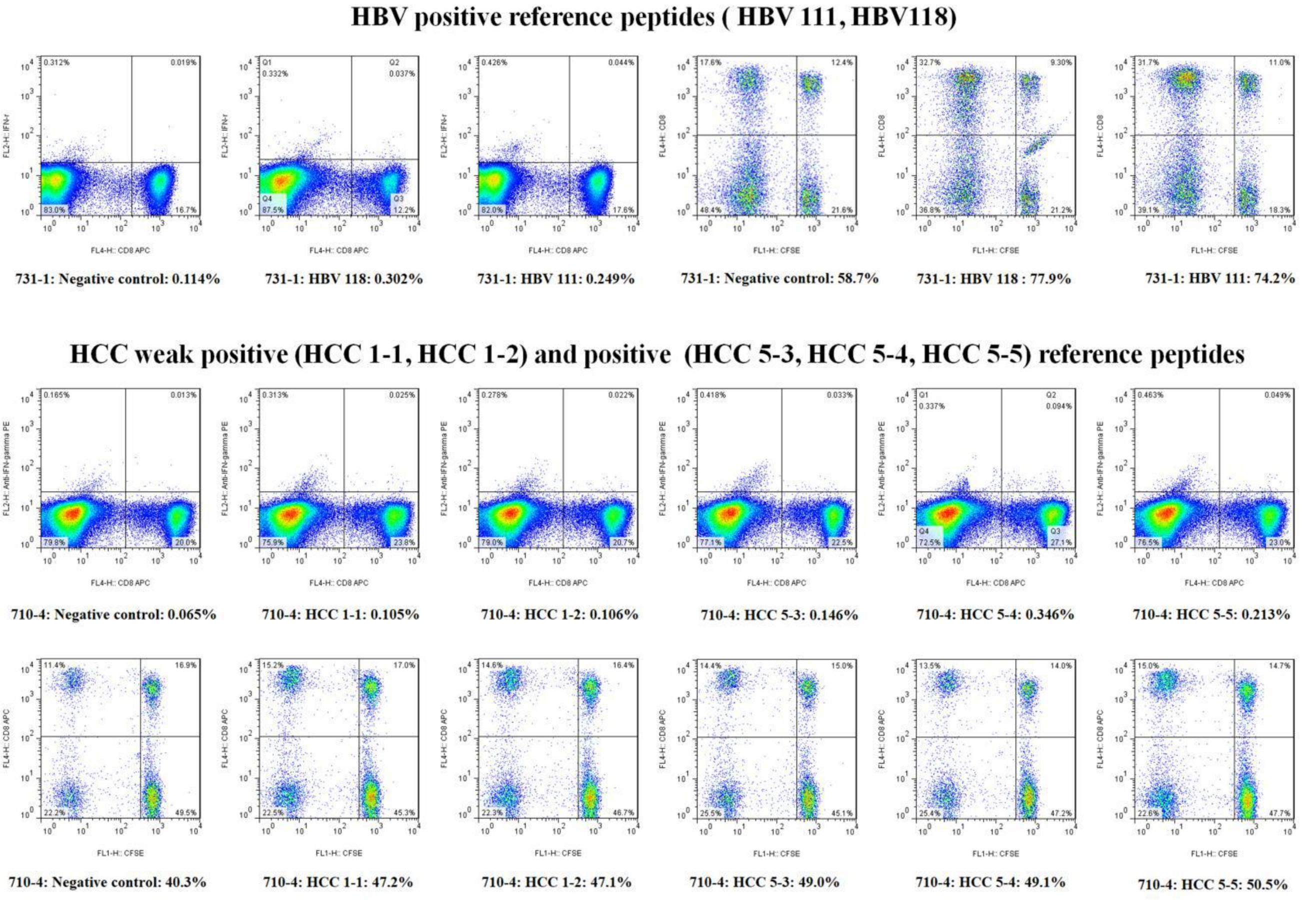

Figure S1: Reference epitope peptides were tested in the DC-peptide-PBL co-culture system (flow plots). The HLA-A0201 restricted HCC1-1, HCC1-2, HCC5-3, HCC5-4, HCC5-5 peptides, and HLA-A2402 restricted HBV111 and HBV118 peptides were co-cultured with DC and PBLs from healthy donor's PBMCs with matching HLA-A allotype for 14 days. The flow plots of IFN-γ^{+} T cells and CFSE staining flow plots of $CD8^{+}$ T cells

in CD3$^+$/CD8$^+$ T cell population for each reference epitope peptide and no peptide negative control were presented. For flow plots of IFN-γ$^+$ T cells, the horizontal coordinates label such as "731-1: HBV118: 0.302%" means that the PBMCs from the donor 731-1 were used to test epitope peptide HBV118 in the DC-peptide-PBL co-cultures, and the frequency of IFN-γ$^+$ cells in CD3$^+$/CD8$^+$ T cell populations was 0.302%; label such as "731-1: Negative control: 0.114%" means that the PBMCs from the donor 731-1 were used in the DC-peptide-PBL co-cultures with no peptide, and the frequency of IFN-γ$^+$ cells in CD3$^+$/CD8$^+$ T cell populations was 0.114%. The rest can be deduced from this manner. For flow plots of CFSE staining, the horizontal coordinates label such as "731-1: HBV118: 77.9%" means that the PBMCs from the donor 731-1 were used to test epitope peptide HBV118 in the DC-peptide-PBL co-cultures, and the proliferation percentage of CD8$^+$ cells in CD3$^+$/CD8$^+$ T cell populations was 77.9%; label such as "731-1: Negative control: 58.7%" means that the PBMCs from the donor 731-1 were used in the DC-peptide-PBL co-cultures with no peptide, and the proliferation percentage of CD8$^+$ cells in CD3$^+$/CD8$^+$ T cell populations was 58.7%. The rest can be deduced from this manner.

Figure S2:

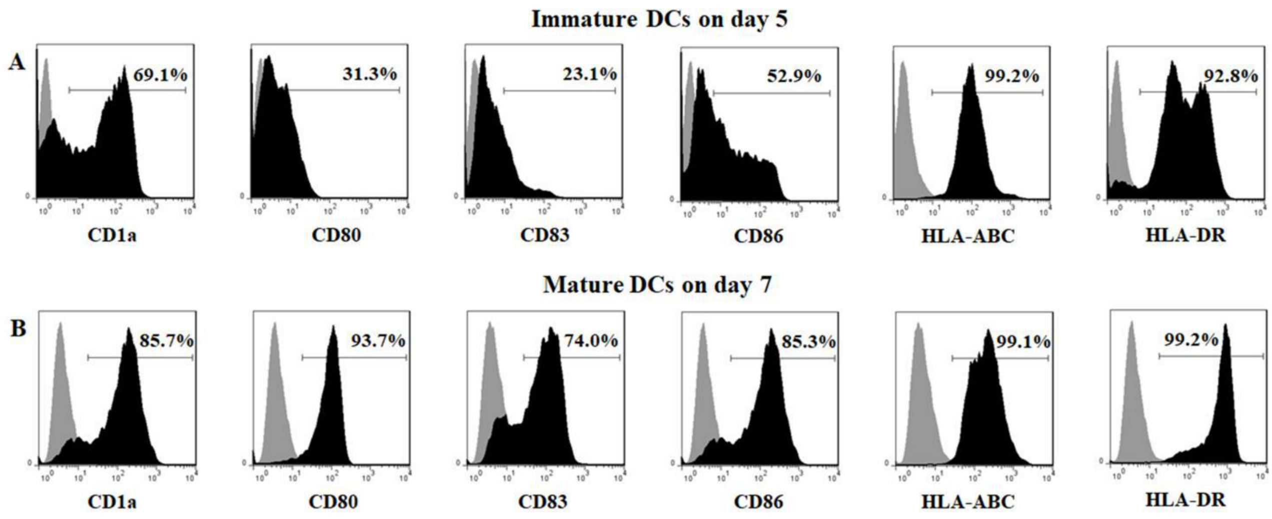

Figure S2: Generation of mature mDC from adherent monocytes. PBMCs from healthy donors were seeded into culture flask and the monocytes adhered for 2 hours as described in the Methods section. After washing out the non-adherent cells in both systems, the cells were cultured for 5 days with 1,000 IU/mL GM-CSF and 500 IU/mL IL-4. Then the immature DCs were matured with 1μg/mL LPS for another 48 hours. Immature DCs and mature DCs were stained for CD1a, CD80, CD83, CD86, HLA-ABC and HLA-DR. The unstained and

stained populations in the histograms are shown in grey and black, respectively. **(A)** Phenotype of immature DCs on day 5. **(B)** Phenotype of mature DCs on day 7.

Figure S3:

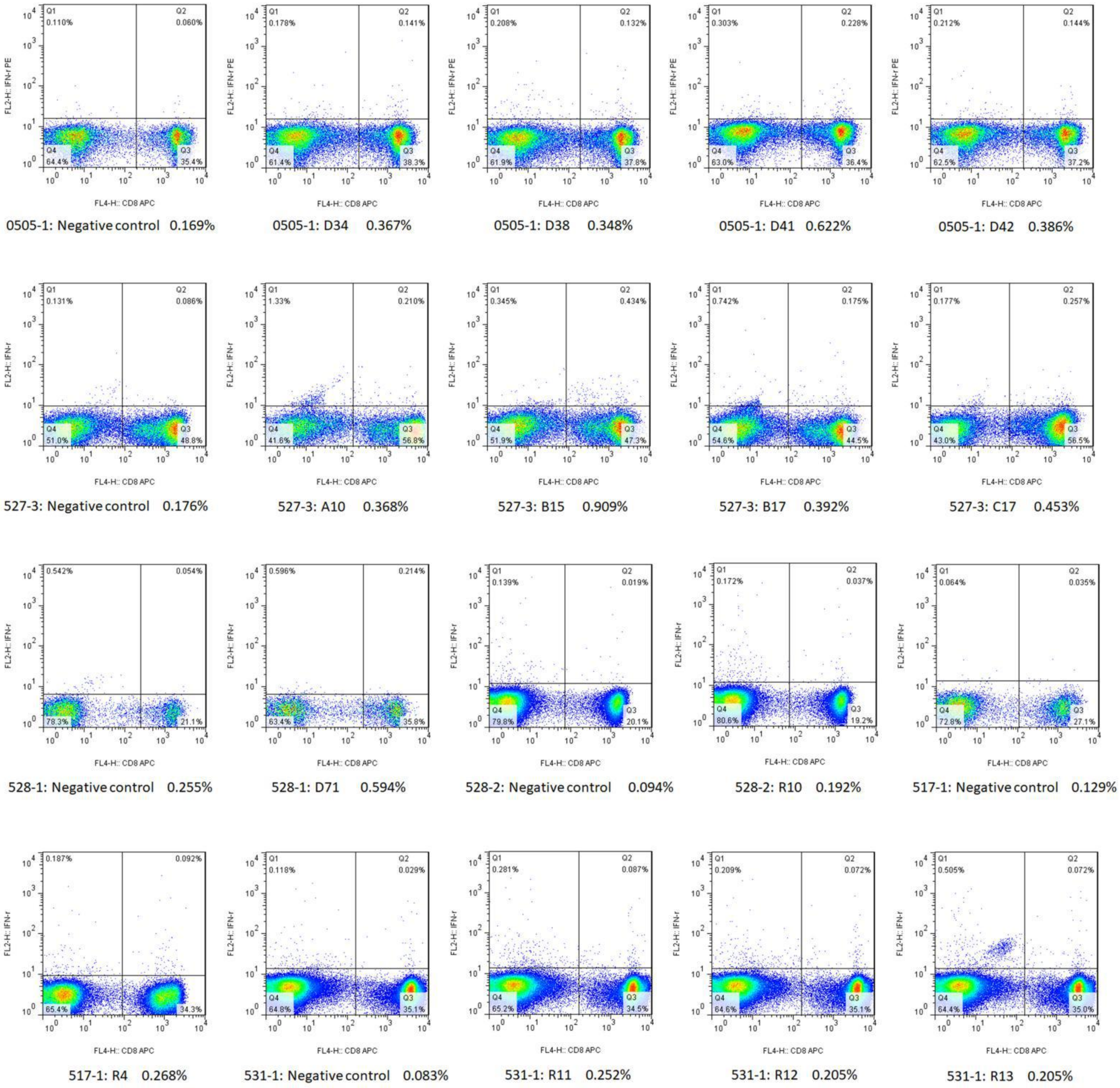

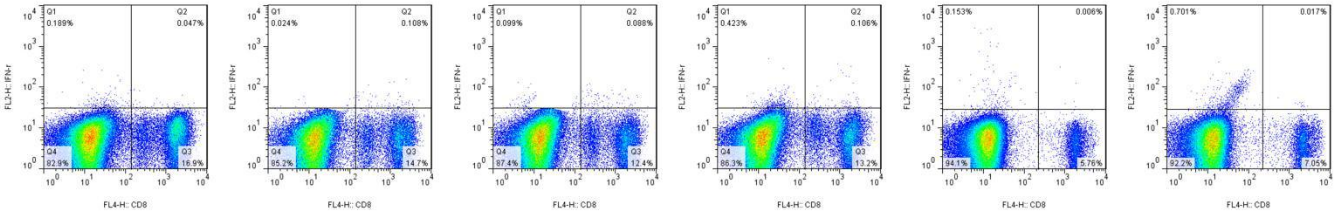

623-1: Negative control 0.277% | 623-1: A9 0.729% | 623-1: B11 0.705% | 623-1: C16 0.797% | 623-4: Negative control 0.104% | 623-4: A9 0.241%

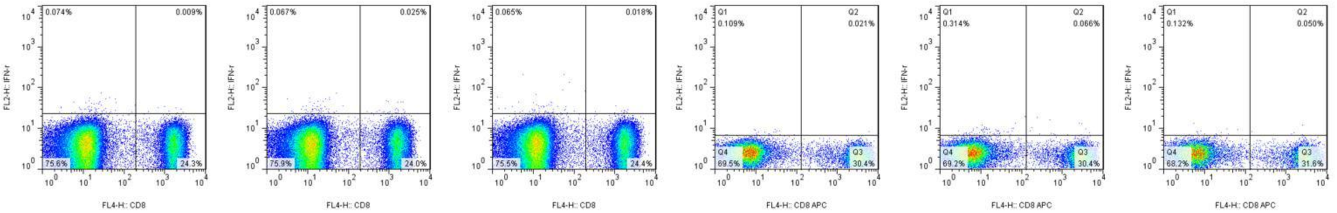

623-3: Negative control 0.037% | 623-3: C45 0.104% | 623-3: C46 0.074% | 701-1: Negative control 0.069% | 701-1: B26 0.217% | 701-1: B31 0.158%

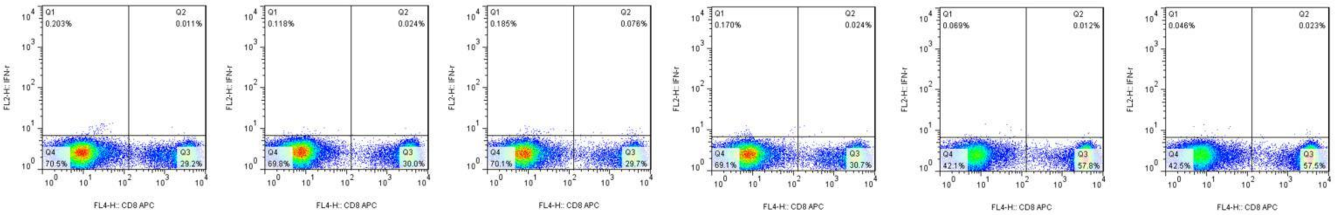

701-2: Negative control 0.037% | 701-2: A4 0.080% | 701-2: A5 0.255% | 701-2: B1 0.078% | 701-4: Negative control 0.020% | 701-4: D40 0.040%

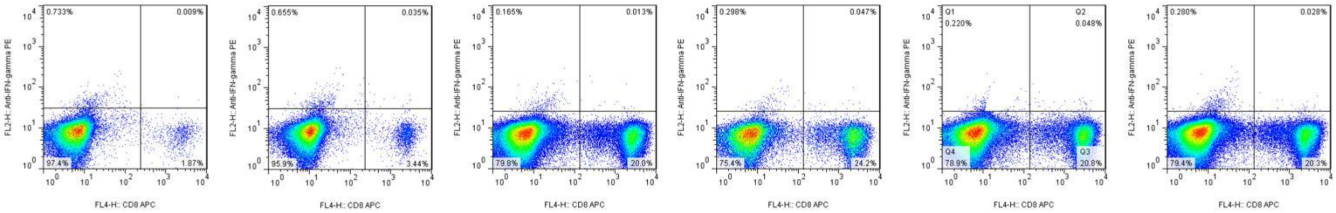

710-2: Negative control 0.479% | 710-2: D55 1.007% | 710-3: Negative control 0.065% | 710-3: A6 0.194% | 710-3: A7 0.230% | 710-3: D17 0.138%

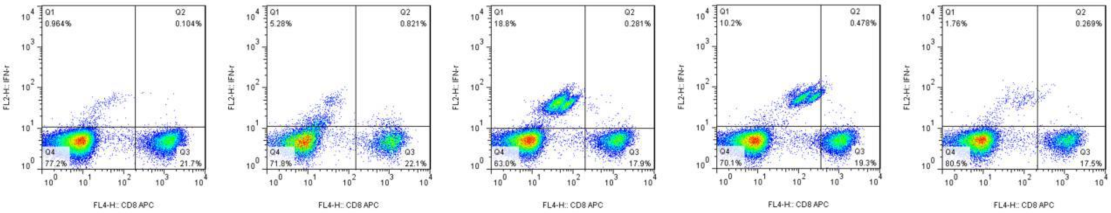

714-2: Negative control 0.477% | 714-2: A5 3.58% | 714-2: B1 1.54% | 714-2: B3 2.42% | 714-2: B4 1.51%

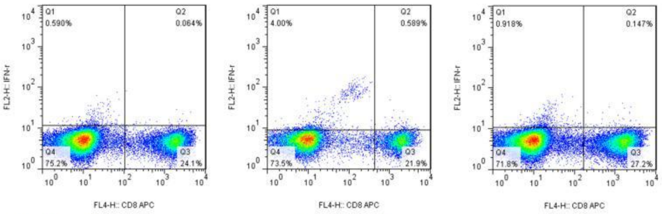

714-3: Negative control 0.265% | 714-3: B10 2.62% | 714-3: R17 0.538%

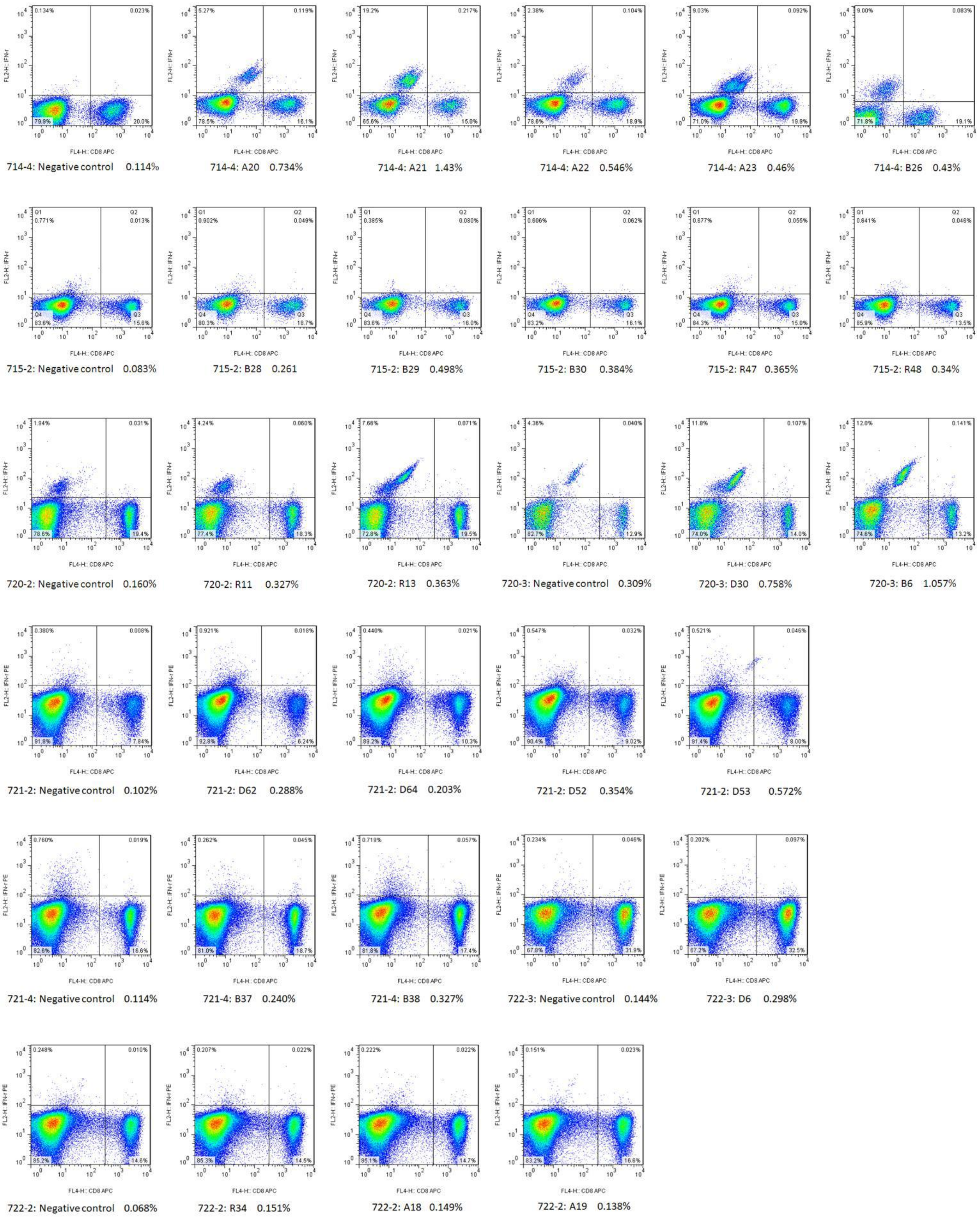

714-4: Negative control 0.114% | 714-4: A20 0.734% | 714-4: A21 1.43% | 714-4: A22 0.546% | 714-4: A23 0.46% | 714-4: B26 0.43%

715-2: Negative control 0.083% | 715-2: B28 0.261 | 715-2: B29 0.498% | 715-2: B30 0.384% | 715-2: R47 0.365% | 715-2: R48 0.34%

720-2: Negative control 0.160% | 720-2: R11 0.327% | 720-2: R13 0.363% | 720-3: Negative control 0.309% | 720-3: D30 0.758% | 720-3: B6 1.057%

721-2: Negative control 0.102% | 721-2: D62 0.288% | 721-2: D64 0.203% | 721-2: D52 0.354% | 721-2: D53 0.572%

721-4: Negative control 0.114% | 721-4: B37 0.240% | 721-4: B38 0.327% | 722-3: Negative control 0.144% | 722-3: D6 0.298%

722-2: Negative control 0.068% | 722-2: R34 0.151% | 722-2: A18 0.149% | 722-2: A19 0.138%

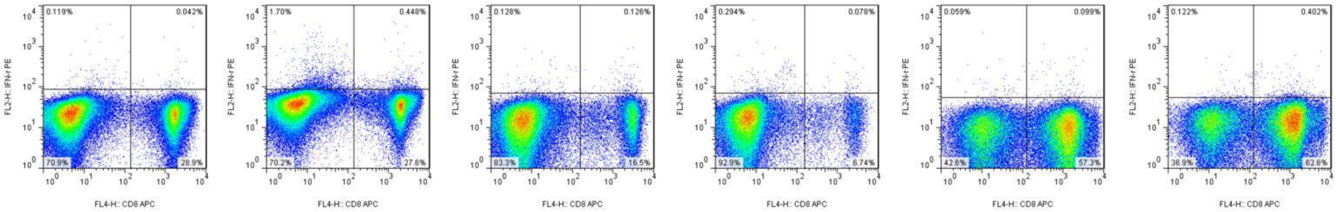

722-4: Negative control 0.145% | 722-4: D13 1.597% | 724-2: Negative control 0.566% | 724-2: B34 1.144% | 724-4: Negative control 0.172% | 724-4: C3 0.638%

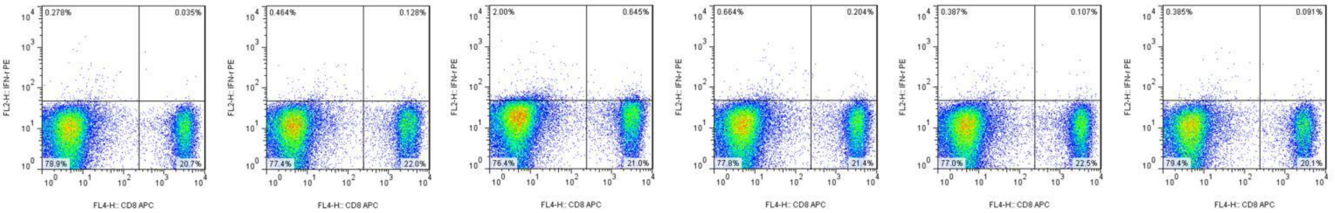

724-3: Negative control 0.169% | 724-3: D50 0.578% | 724-3: C49 2.980% | 724-3: D46 0.944% | 724-3: D47 0.473% | 724-3: D48 0.451%

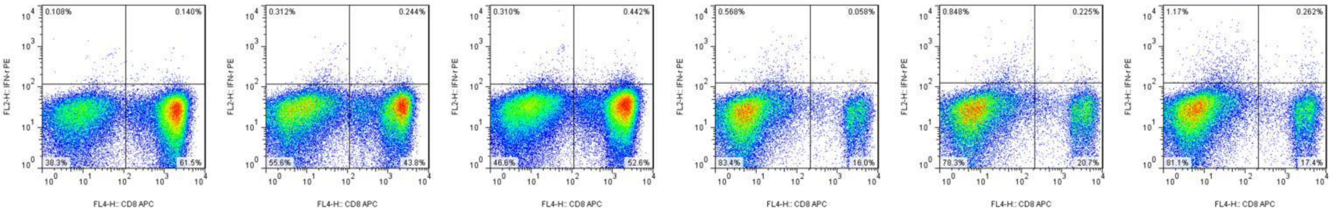

727-1: Negative control 0.227% | 727-1: C35 0.554% | 727-1: D56 0.833% | 727-3: Negative control 0.361% | 727-3: C47 1.075% | 727-3: D76 1.483%

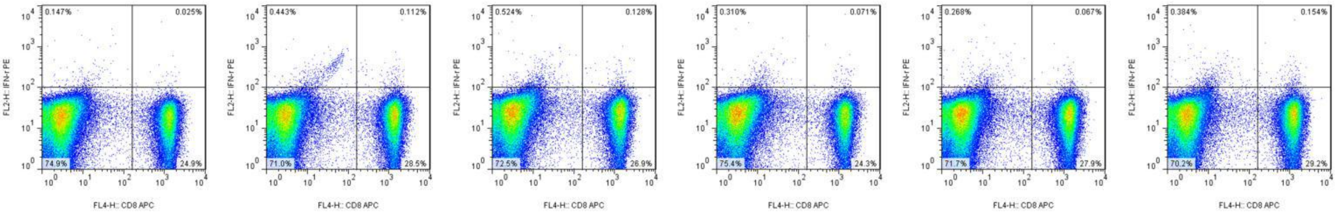

727-2: Negative control 0.100% | 727-2: A26 0.391% | 727-2: B36 0.474% | 727-2: B37 0.291% | 727-2: B40 0.240% | 727-2: B41 0.525%

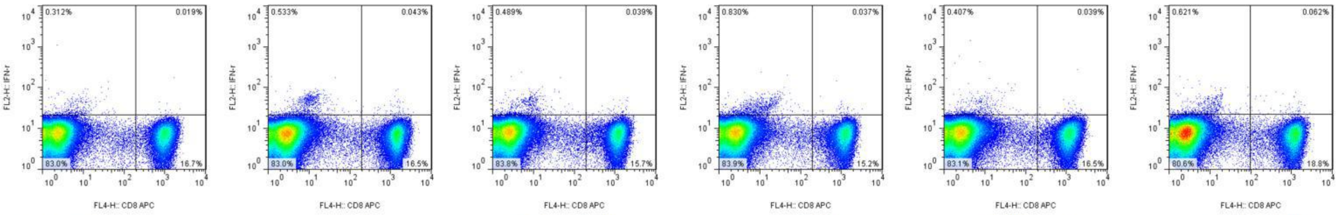

731-4: Negative control 0.114% | 731-4: B2 0.26% | 731-4: R5 0.248% | 731-4: R8 0.243% | 731-4: R9 0.236% | 731-4: R12 0.329%

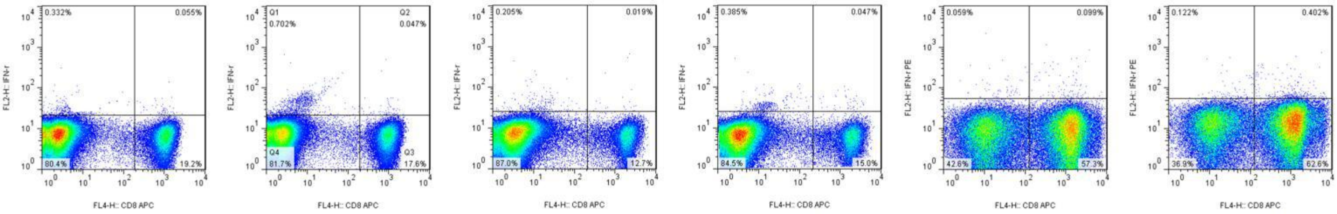

731-4: R13 0.286% | 731-4: R14 0.266% | 731-1: Negative control 0.149% | 731-1: R23 0.312% | 724-4: Negative control 0.172% | 724-4: C3 0.638%

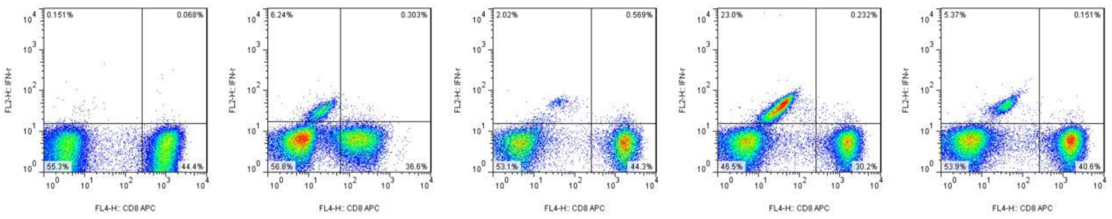

820-3: Negative control 0.153% 820-3: A4 0.821% 820-3: A5 1.268% 820-3: B1 0.762% 820-3: B29 0.371%

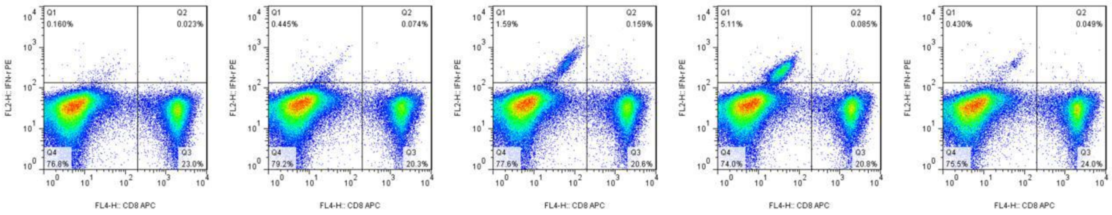

821-1: Negative control 0.100% 821-1: A6 0.363% 821-1: A9 0.766% 821-1: B11 0.407% 821-1: B12 0.204%

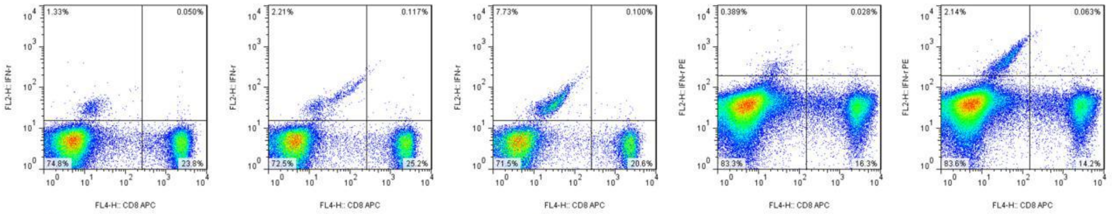

820-2: Negative control 0.21% 820-2: B10 0.462% 820-2: A1 0.483% 821-2: Negative control 0.171% 821-2: A12 0.442%

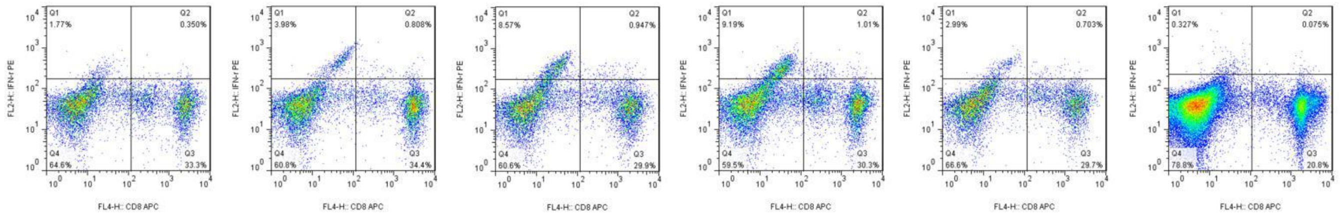

822-1: Negative control 1.040% 822-1: A1 2.295% 822-1: A5 3.070% 822-1: B1 3.226% 822-1: D2 2.312% 822-2: Negative control 0.359%

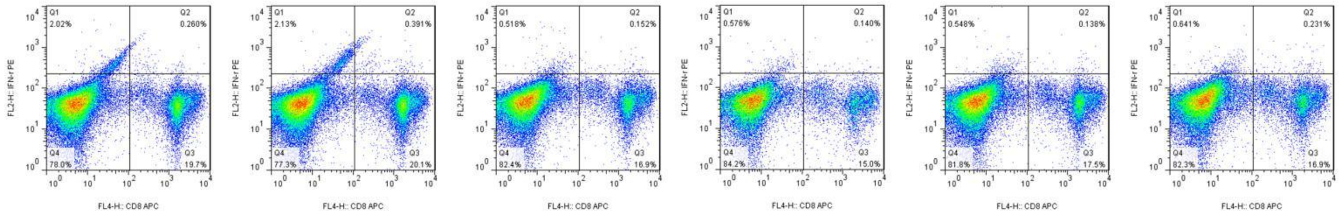

822-2: A12 1.303% 822-2: B23 1.908% 822-2: B35 0.891% 822-2: D72 0.925% 822-2: D48 0.782% 822-2: D53 1.348%

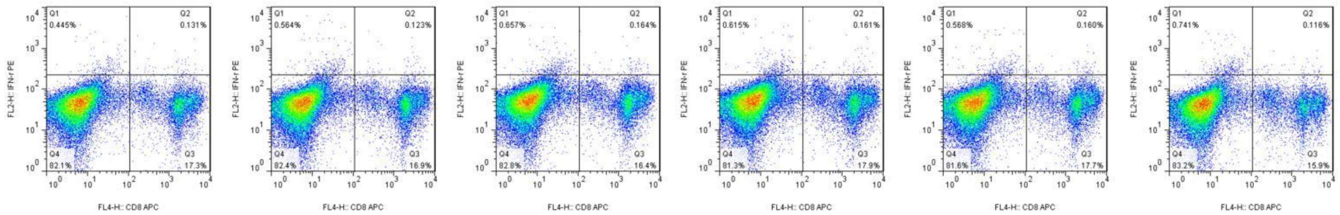

822-2: D77 0.752% 822-2: D78 0.723% 822-2: D79 0.990% 822-2: D80 0.891% 822-2: D81 0.896% 822-2: D82 0.724%

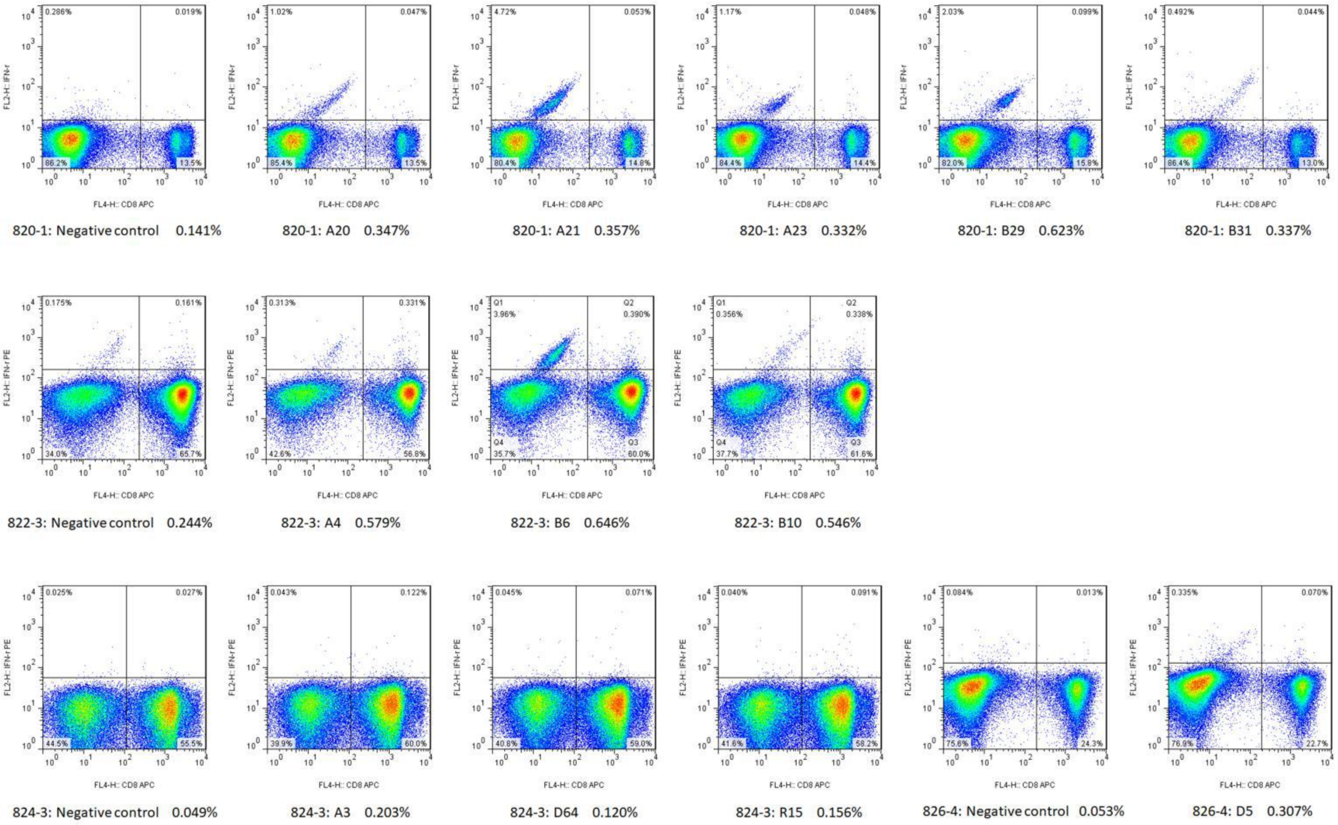

Figure S3: 120 epitopes were validated by DC-peptide-PBL co-culture experiments (IFN-γ ICS flow plots). DCs were induced for 7 days from healthy donor's PBMCs, then coincubated with candidate epitope peptides and autologous PBLs for 14 days. Cells were harvested and stimulated by corresponding candidate peptides for another 16 hours followed by IFN-γ ICS. The presented are flow plots for each positive epitope peptide and each responded donor. The horizontal coordinates label such as "0505-1: D34 0.367%" means that the PBMCs from the donor 0505-1 were used to test epitope peptide D34 in the DC-peptide-PBL co-cultures, and the frequency of IFN-γ^+ cells in $CD3^+/CD8^+$ T cell populations was 0.367%; label such as "0505-1: negative control 0.169%" means that the PBMCs from the donor 0505-1 were used in the DC-peptide-PBL co-cultures with no peptide, and the frequency of IFN-γ^+ cells in $CD3^+/CD8^+$ T cell populations was 0.169%. The rest can be deduced from this manner.

Figure S4:

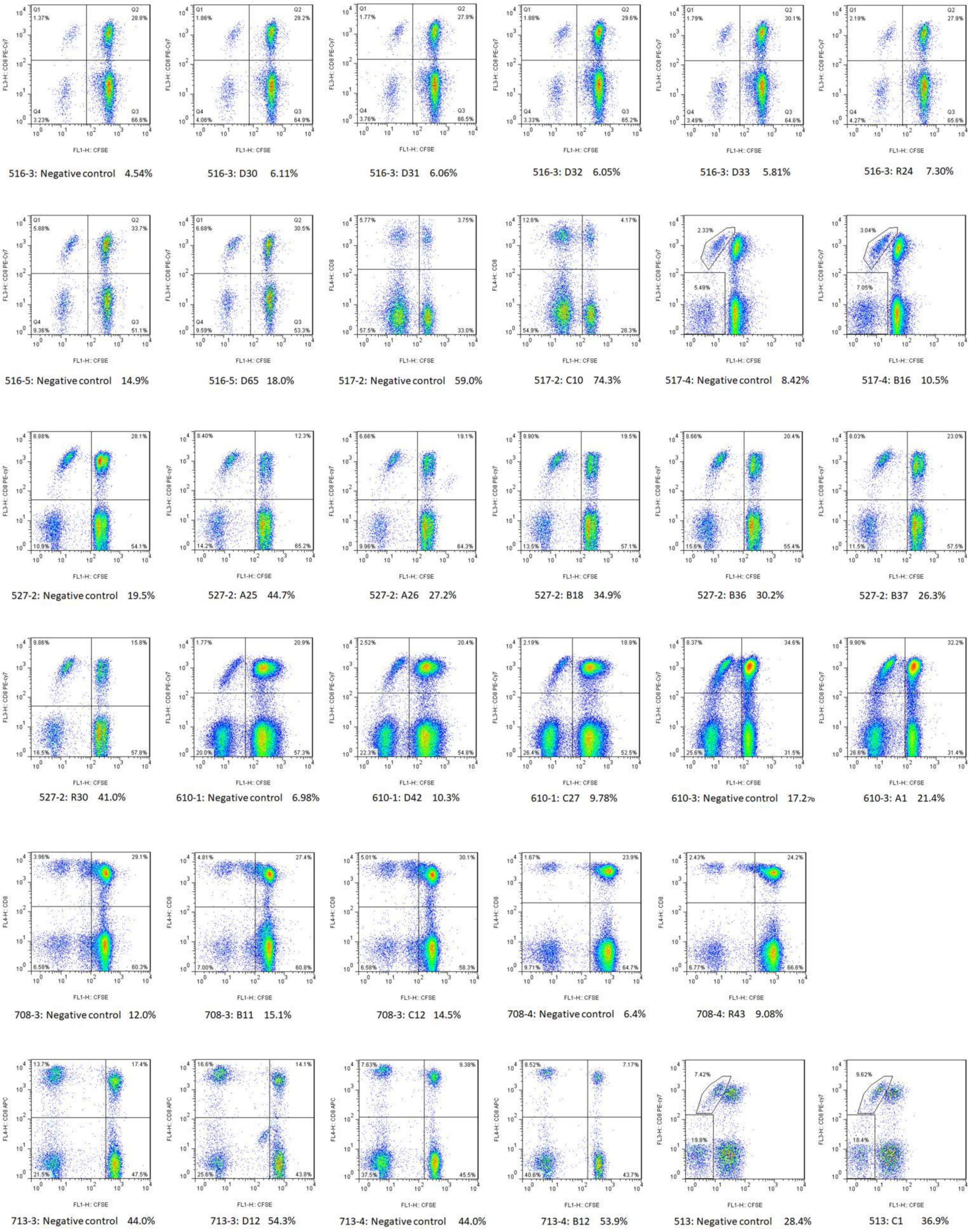
516-3: Negative control 4.54%
516-3: D30 6.11%
516-3: D31 6.06%
516-3: D32 6.05%
516-3: D33 5.81%
516-3: R24 7.30%
516-5: Negative control 14.9%
516-5: D65 18.0%
517-2: Negative control 59.0%
517-2: C10 74.3%
517-4: Negative control 8.42%
517-4: B16 10.5%
527-2: Negative control 19.5%
527-2: A25 44.7%
527-2: A26 27.2%
527-2: B18 34.9%
527-2: B36 30.2%
527-2: B37 26.3%
527-2: R30 41.0%
610-1: Negative control 6.98%
610-1: D42 10.3%
610-1: C27 9.78%
610-3: Negative control 17.2%
610-3: A1 21.4%
708-3: Negative control 12.0%
708-3: B11 15.1%
708-3: C12 14.5%
708-4: Negative control 6.4%
708-4: R43 9.08%
713-3: Negative control 44.0%
713-3: D12 54.3%
713-4: Negative control 44.0%
713-4: B12 53.9%
513: Negative control 28.4%
513: C1 36.9%

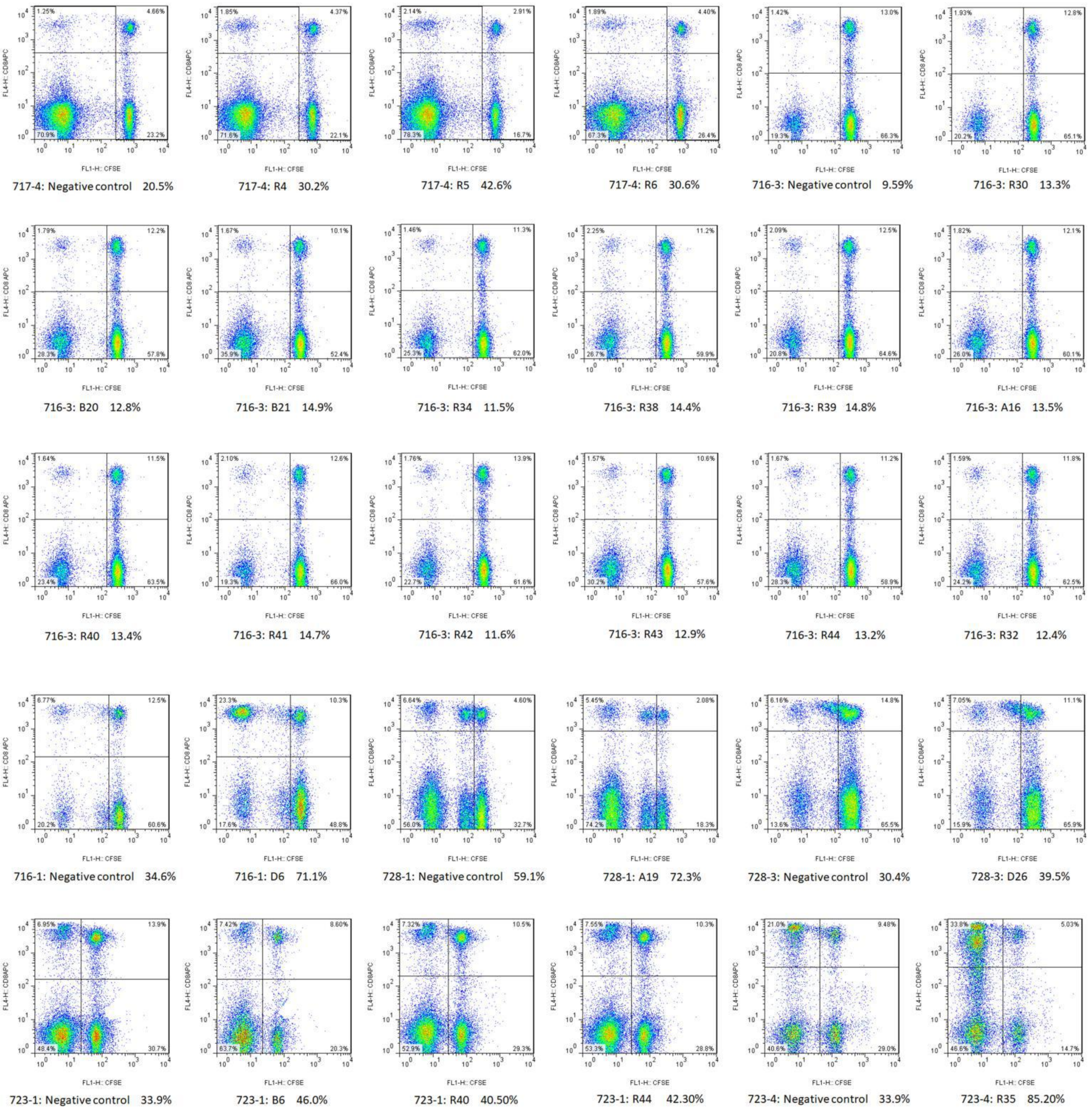

Figure S4: 120 candidate epitopes were validated by DC-peptide-PBL co-culture experiments (CFSE staining flow plots). DCs were coincubated with candidate epitope peptides and CFSE-prelabeled PBLs for 14 days. Cells were then analyzed by flow cytometry. The presented are flow plots for each positive epitope peptide and each responded donor. The horizontal coordinates label such as "516-3: D30 6.11%" means that the PBMCs from the donor 516-3 were used to test epitope peptide D30 in the DC-peptide-PBL co-cultures, and the proliferation percentage of $CD8^+$ cells in $CD3^+$/$CD8^+$ T cell populations was 6.11%; label such as "516-3: negative control 4.54%" means that the PBMCs from the donor 516-3 were used in the DC-peptide-PBL co-cultures with no peptide, and the

proliferation percentage of $CD8^+$ cells in $CD3^+/CD8^+$ T cell populations was 4.54%. The rest can be deduced from this manner.

Figure S5:

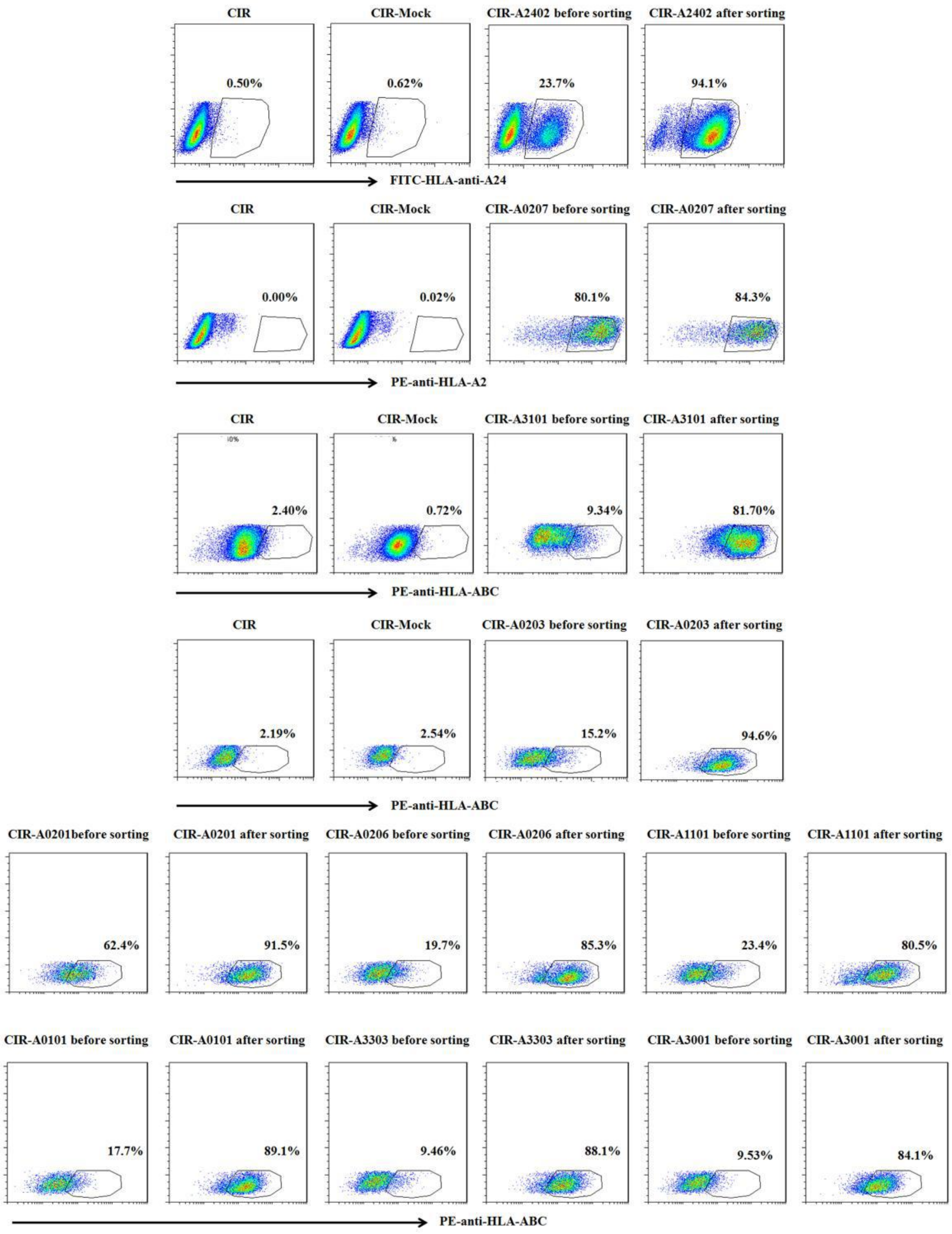

Figure S5: Ten HMy2.CIR cell lines expressing indicated HLA-A allotype. The transfected Hmy2.1 CIR cell lines expressing HLA-A2402, A0207, A0201, A0203, A0206, A0101, A1101, A3101, A3303, or A3001 were generated, respectively, and stained with FITC-anti–HLA-A24, PE-anti-HLA-A2.1 or PE-anti-HLA-ABC (W6/32), then sorted by

flow cytometry. The purity of these transfected HMy2.CIR cell lines was 80% to 94% after sorting.

Figure S6:

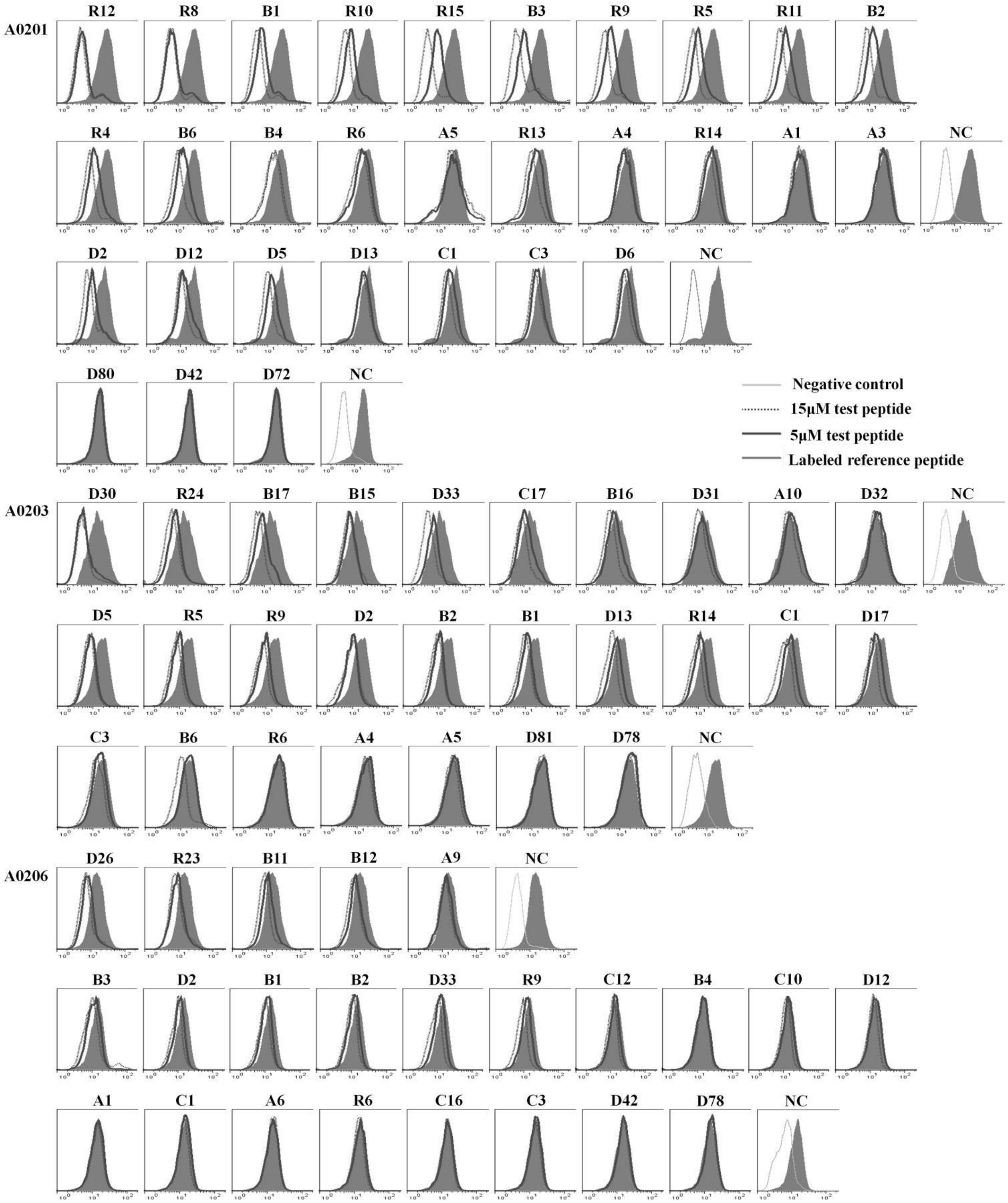

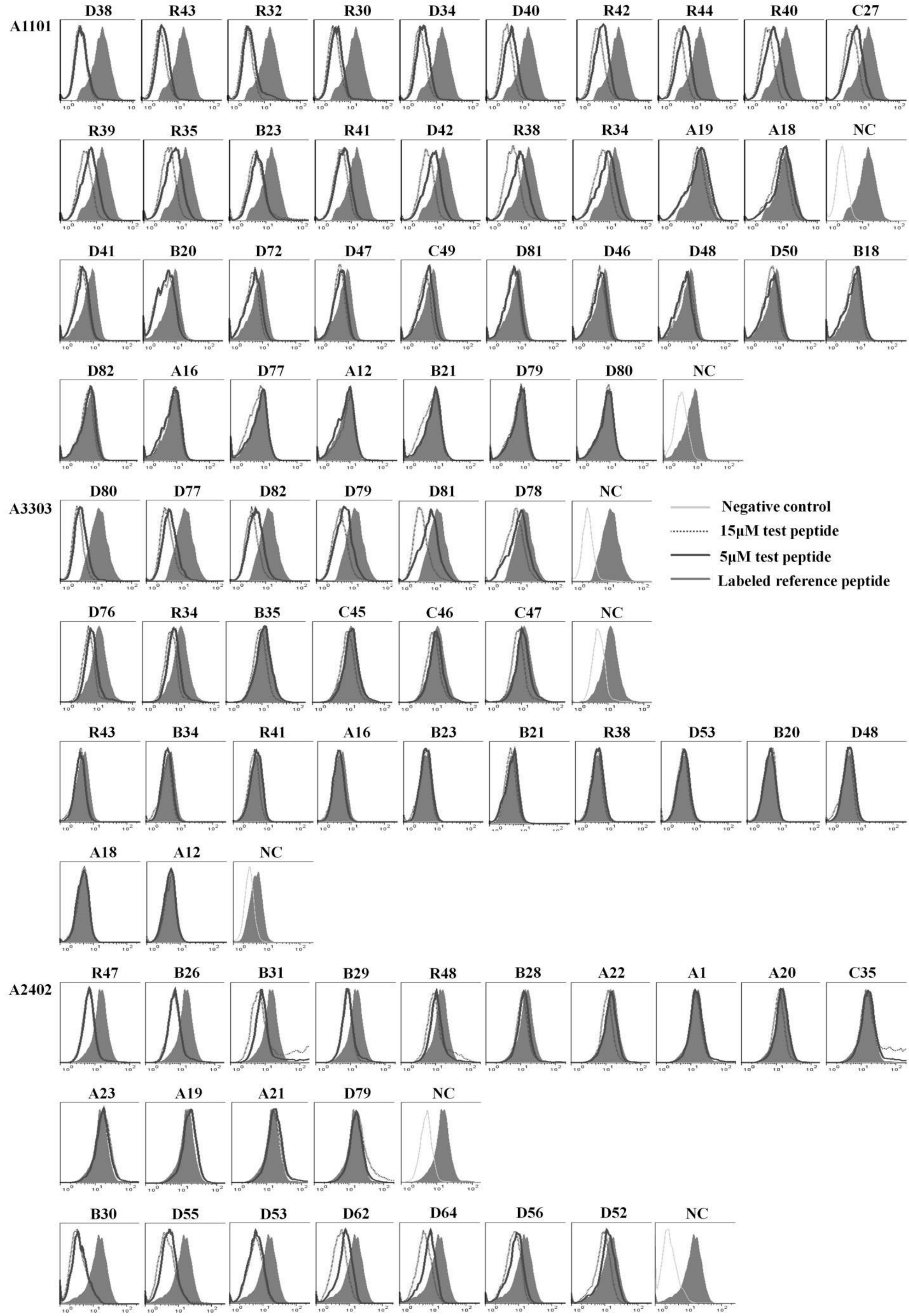
A1101
D38
R43
R32
R30
D34
D40
R42
R44
R40
C27
R39
R35
B23
R41
D42
R38
R34
A19
A18
NC
D41
B20
D72
D47
C49
D81
D46
D48
D50
B18
D82
A16
D77
A12
B21
D79
D80
NC
A3303
D80
D77
D82
D79
D81
D78
NC
Negative control
15μM test peptide
5μM test peptide
Labeled reference peptide
D76
R34
B35
C45
C46
C47
NC
R43
B34
R41
A16
B23
B21
R38
D53
B20
D48
A18
A12
NC
A2402
R47
B26
B31
B29
R48
B28
A22
A1
A20
C35
A23
A19
A21
D79
NC
B30
D55
D53
D62
D64
D56
D52
NC

Figure S6: Binding affinity of 120 validated epitopes with HLA-A allotypes as defined by peptide competitive binding experiments. A series of unlabeled epitope peptides of SRAS-CoV-2 were coincubated, at 5μM and 15μM respectively, with fluorescent-labeled reference peptides and CIR cell lines expressing the corresponding HLA-A molecules for 24 hours. Then the competitively binding inhibition (%) of the epitope peptide at 5μM and 15μM was calculated by measuring the CIR cells fluorescence strength. Shown are the histograms of two concentrations (5μM and 15μM). Black solid line was the histogram of 5μM test peptide; dotted line was the histogram of 15μM, test peptide; black filled line was the maximal fluorescence (FITC-labeled reference peptide without competitive peptides) while the lightest gray line was the negative control (background fluorescence with 1640 alone). NC: the maximal fluorescence (FITC-labeled reference peptide without competitive peptides) and background fluorescence with 1640 alone.

Figure S7:

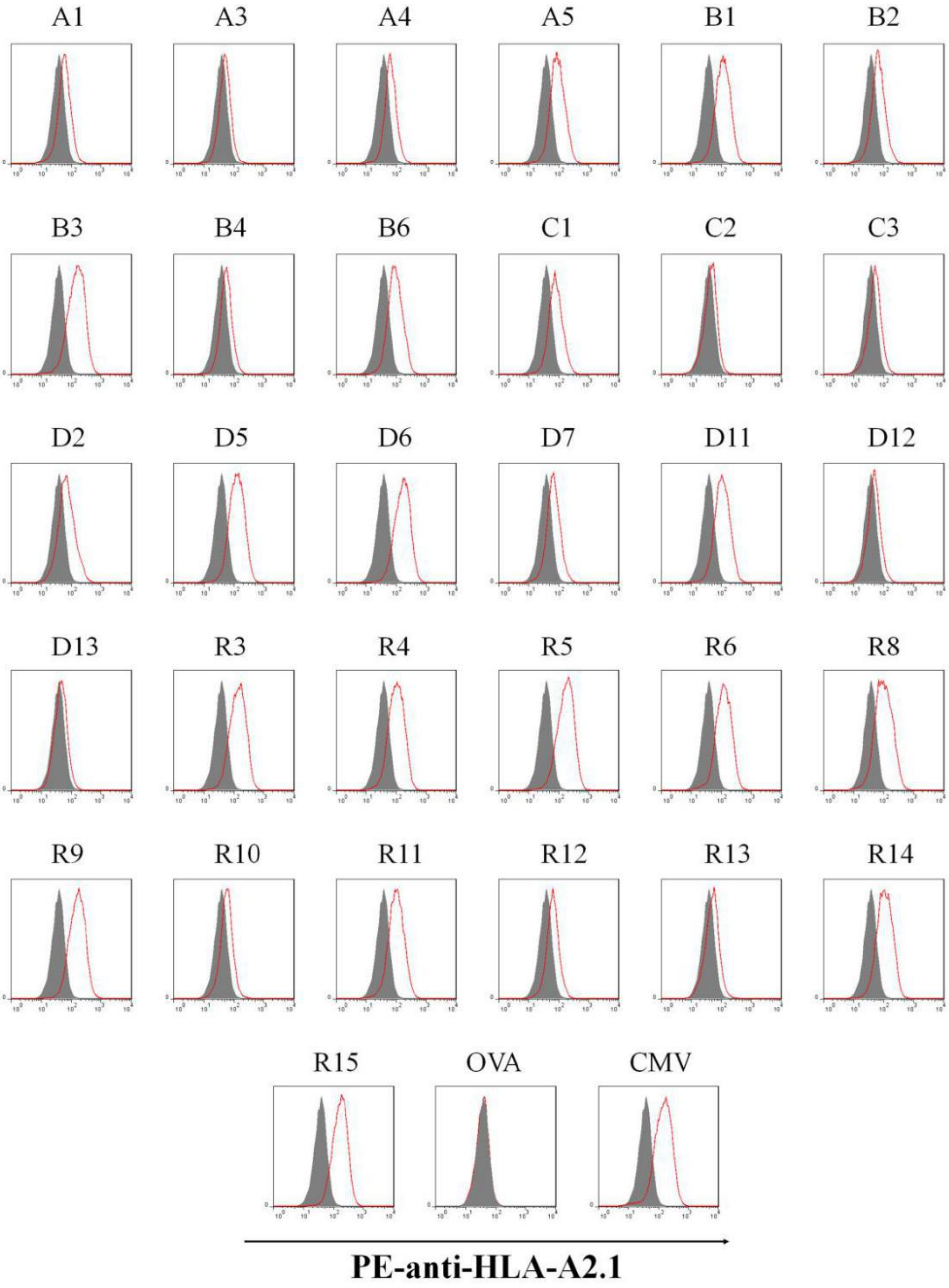

Figure S7: Binding affinity of 31 validated epitopes with HLA-A0201 molecule as defined by T2 cell binding assay. T2 cells were incubated with single peptide of the 31 epitopes, or with CMVpp65$_{495\text{-}503}$ peptide as positive control, OVA$_{257\text{-}264}$ peptide as negative control, or no peptide and β2-m for 16 hours, then followed by PE-labeled anti-HLA-A2.1 antibody staining to test the up-regulation of HLA-A0201 molecules onto T2 cells. The fluorescence index (FI) was calculated with flow cytometry. Red solid line was the histogram of indicated peptides while the black filled line was background fluorescence of T2 cells alone without peptide. OVA: OVA$_{257\text{-}264}$ peptide; CMV: CMVpp65$_{495\text{-}503}$ peptide.

Figure S8:

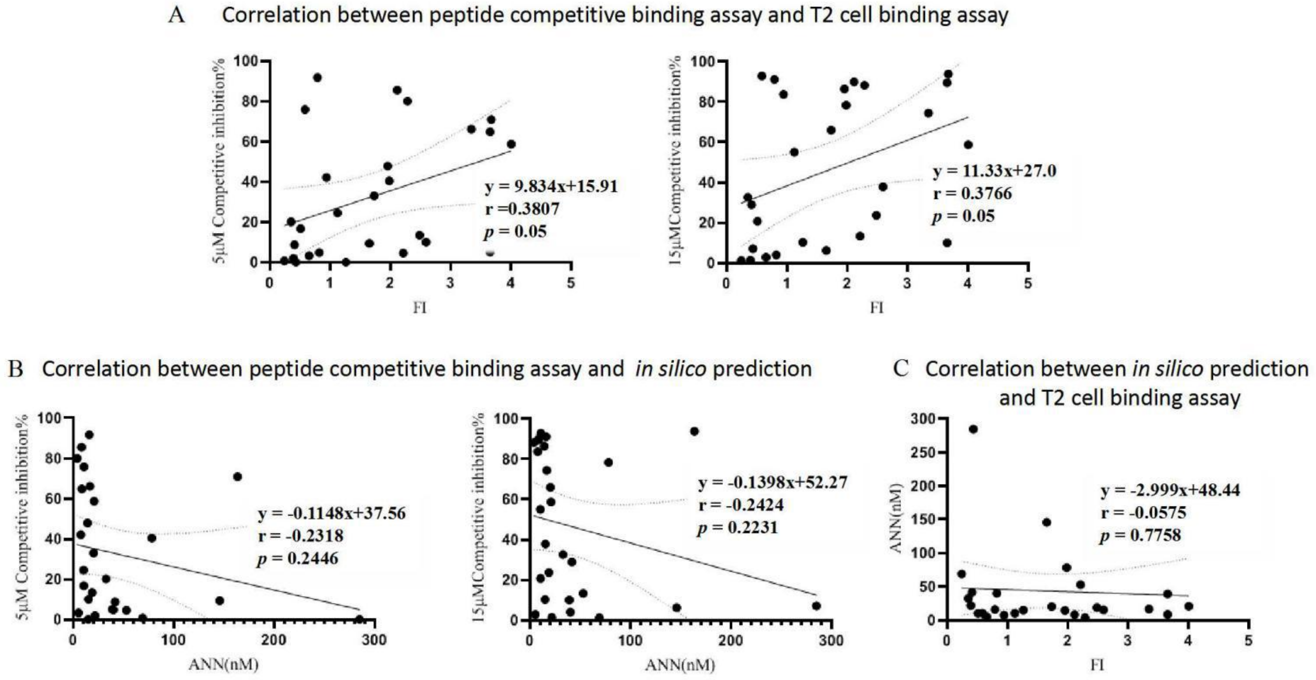

Figure S8: Correlation coefficancy across approaches for the 27 validated epitope peptides restricted by HLA-A0201 molecules. 5μM or 15μM competitive inhibition % means the competitively binding inhibition (%) of validated epitope peptide at 5μM or 15μM; FI means the fluoroscence index in T2 cell binding assay; ANN(nM) means the *in silico* predicted affinity using IDEB ANN alograthm. P<0.05 indicates that the correlation analysis had statistical significance.

Figure S9:

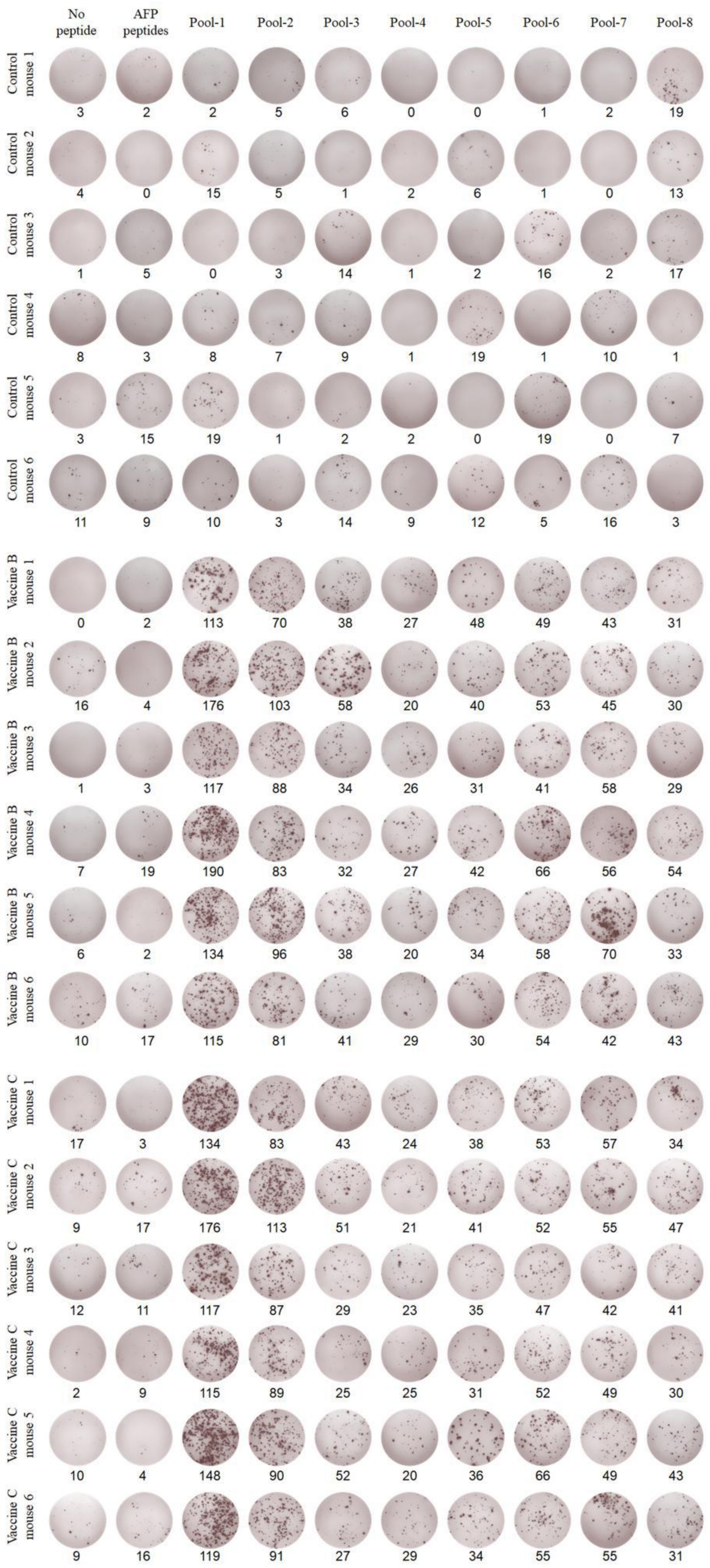

Figure S9: IFN-γ ELISPOT spot plots against the individual peptide pools in hybrid mice. Splenocytes from each primed hybrid mouse were harvested 7 days after the last booster and *ex vivo* stimulated with 8 different peptide pools covering the 31 VEPs or with AFP peptides ($AFP_{158-166}$, $AFP_{424-432}$) as irrelevant control, or without peptide as negative control, and followed by IFN-γ ELISPOT. The hybrid mice: the hybrid of HLA-A0201$^{+/+}$/DR1$^{+/+}$/H-2-β2m$^{-/-}$/I-Aβ$^{-/-}$ C57BL/6 mice and WT C57BL/6 mice; Control group: normal saline; Vaccine B group: R848/peptides vaccines; Vaccine C group: Poly I: C/peptides vaccines.

Figure S10:

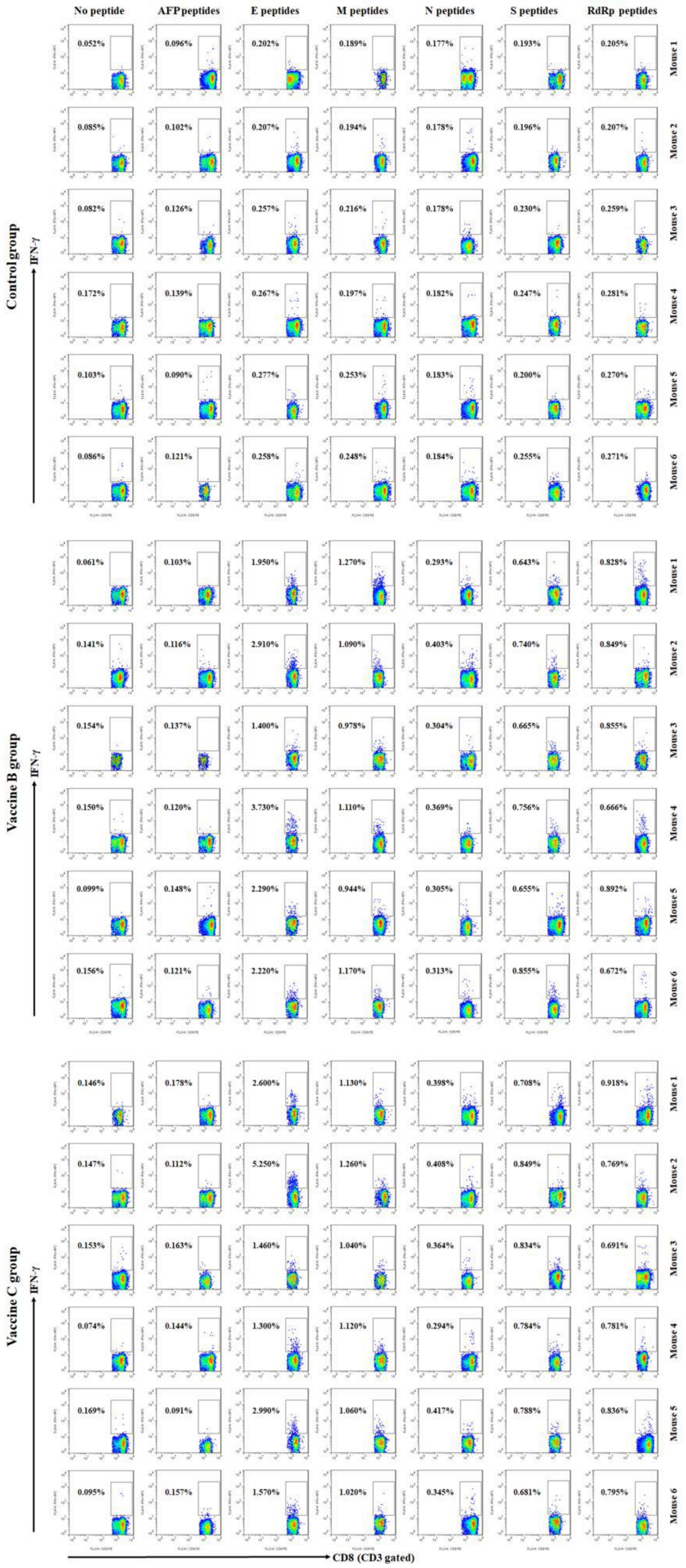

Figure S10: Flow plots of IFN-γ ICS responding to the individual peptide pools in hybrid mice. Splenocytes from each primed hybrid mouse were harvested 7 days after the last booster and *ex vivo* stimulated with 5 different peptide pools (1 pool/protein) covering the 31 VEPs or with AFP peptides ($AFP_{158-166}$, $AFP_{424-432}$) as irrelevant control, or without peptide as negative control, and followed by IFN-γ ICS. The data in left upper quadrant mean the frequencies of IFN-γ^{+} T cells in $CD3^{+}$/$CD8^{+}$ cell populations. The hybrid mice were the hybrid of HLA-A0201$^{+/+}$/DR1$^{+/+}$/H-2-β2 m$^{-/-}$/I-Aβ$^{-/-}$ C57BL/6 mice and WT C57BL/6 mice;
Control group: normal saline;
Vaccine B group:
R848/peptides vaccines;
Vaccine C group:
Poly I: C/peptides vaccines.

Figure S11:

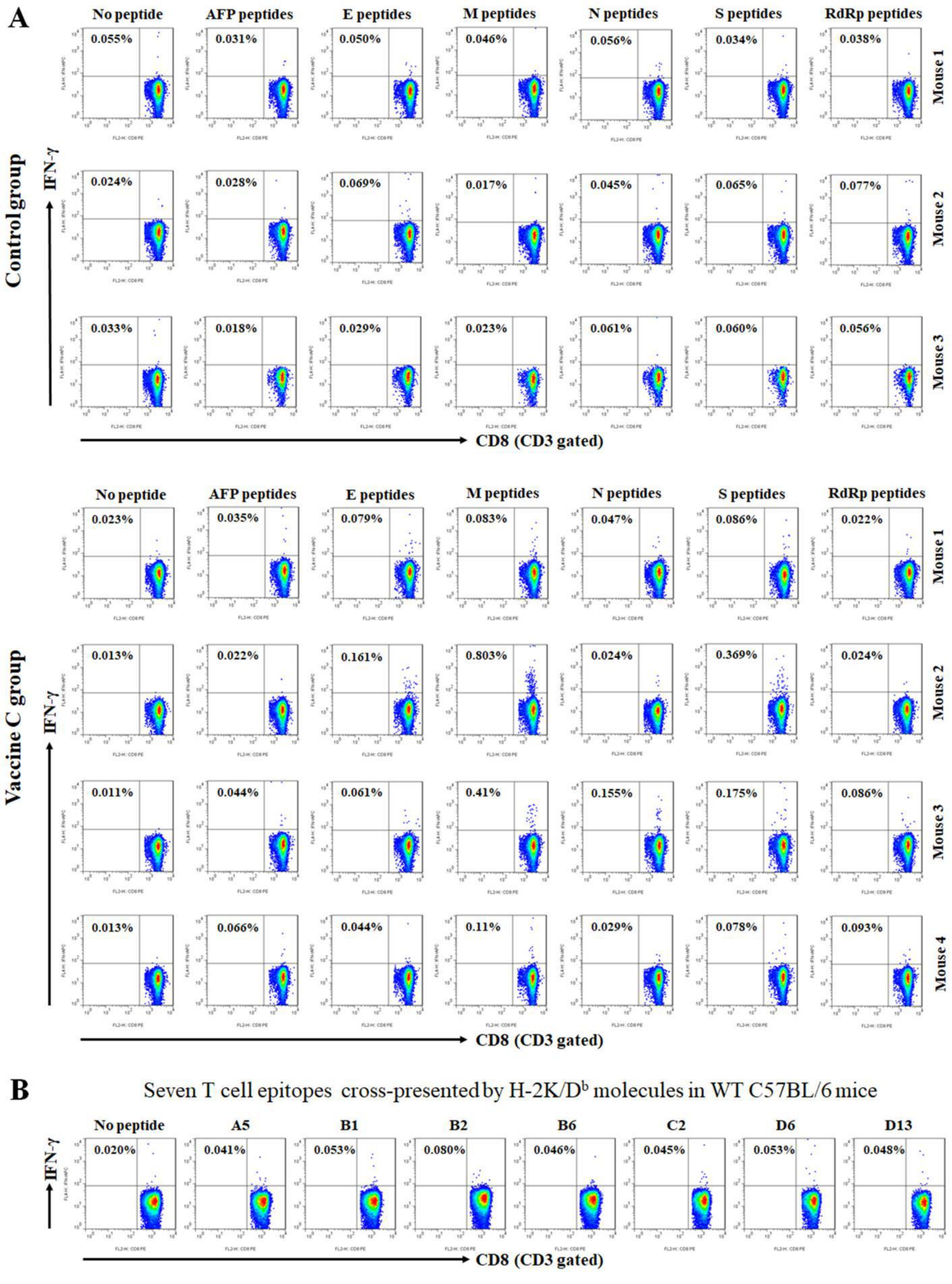
A
No peptide
AFP peptides
E peptides
M peptides
N peptides
S peptides
RdRp peptides
Control group
IFN-γ
Mouse 1
Mouse 2
Mouse 3
0.055%
0.031%
0.050%
0.046%
0.056%
0.034%
0.038%
0.024%
0.028%
0.069%
0.017%
0.045%
0.065%
0.077%
0.033%
0.018%
0.029%
0.023%
0.061%
0.060%
0.056%
CD8 (CD3 gated)
Vaccine C group
Mouse 4
0.023%
0.035%
0.079%
0.083%
0.047%
0.086%
0.022%
0.013%
0.022%
0.161%
0.803%
0.024%
0.369%
0.024%
0.011%
0.044%
0.061%
0.41%
0.155%
0.175%
0.086%
0.013%
0.066%
0.044%
0.11%
0.029%
0.078%
0.093%
B
Seven T cell epitopes cross-presented by H-2K/D^b molecules in WT C57BL/6 mice
No peptide
A5
B1
B2
B6
C2
D6
D13
0.020%
0.041%
0.053%
0.080%
0.046%
0.045%
0.053%
0.048%

Figure S11: Flow plots of IFN-γ ICS responding to the individual peptide pools after WT mice immunizations. (A) Splenocytes from each primed WT mouse were harvested 7 days after the last booster and *ex vivo* stimulated with 5 different peptide pools (1 pool/protein) covering the 31 VEPs or with AFP peptides ($AFP_{158-166}$, $AFP_{424-432}$) as irrelevant control, or without peptide as negative control, and followed by IFN-γ ICS. The data in left upper quadrant mean the frequencies of IFN-γ^+ T cells in $CD3^+/CD8^+$ cell populations. **(B)** 7 of 31 VEPs restricted by HLA-A0201 were identified to be cross-presented by H-2K/D^b molecules. Splenocytes from each primed WT mouse were harvested as described and *ex vivo* stimulated with single peptide, or without peptide as negative control, and followed by IFN-γ ICS. The epitopes were identified as immunogenic peptides when the frequency of IFN-γ^+ T cells in $CD3^+/CD8^+$ cell population reached 2 times over the negative control. Representative flow plots of the 7 positive epitopes were shown. WT mice: wild type C57BL/6 mice; Control group: normal saline; Vaccine C group: Poly I: C/peptides vaccines.

Figure S12:

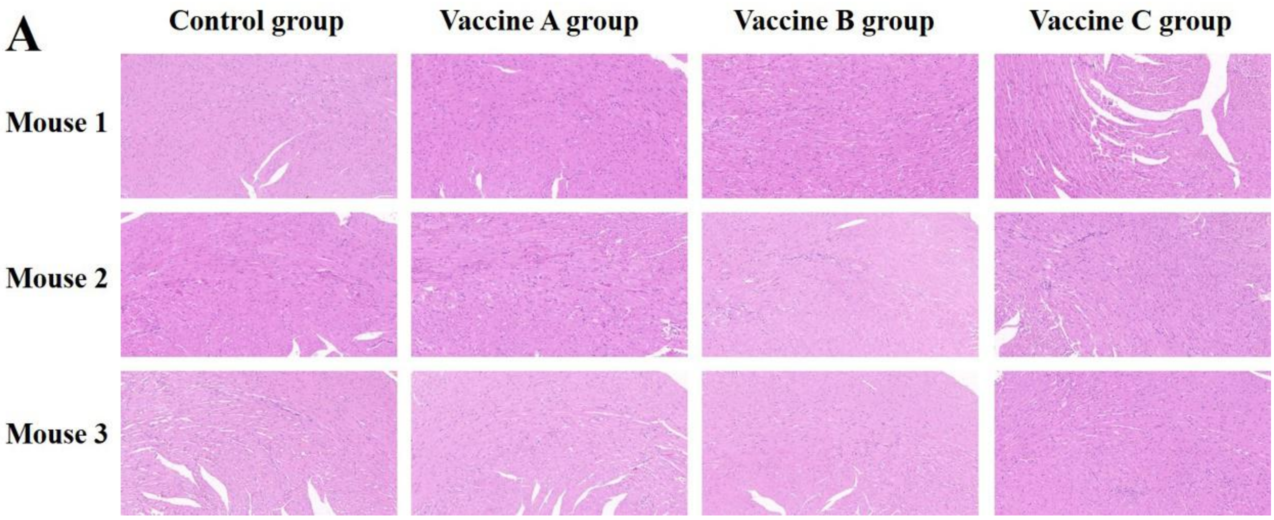
A
Control group
Vaccine A group
Vaccine B group
Vaccine C group
Mouse 1
Mouse 2
Mouse 3

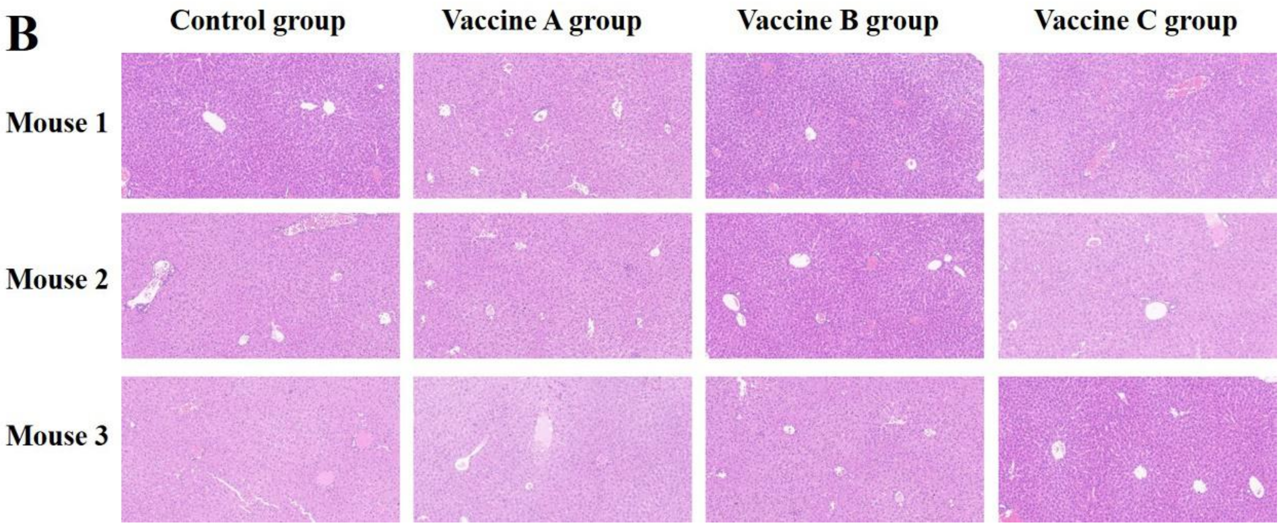
B
Control group
Vaccine A group
Vaccine B group
Vaccine C group
Mouse 1
Mouse 2
Mouse 3

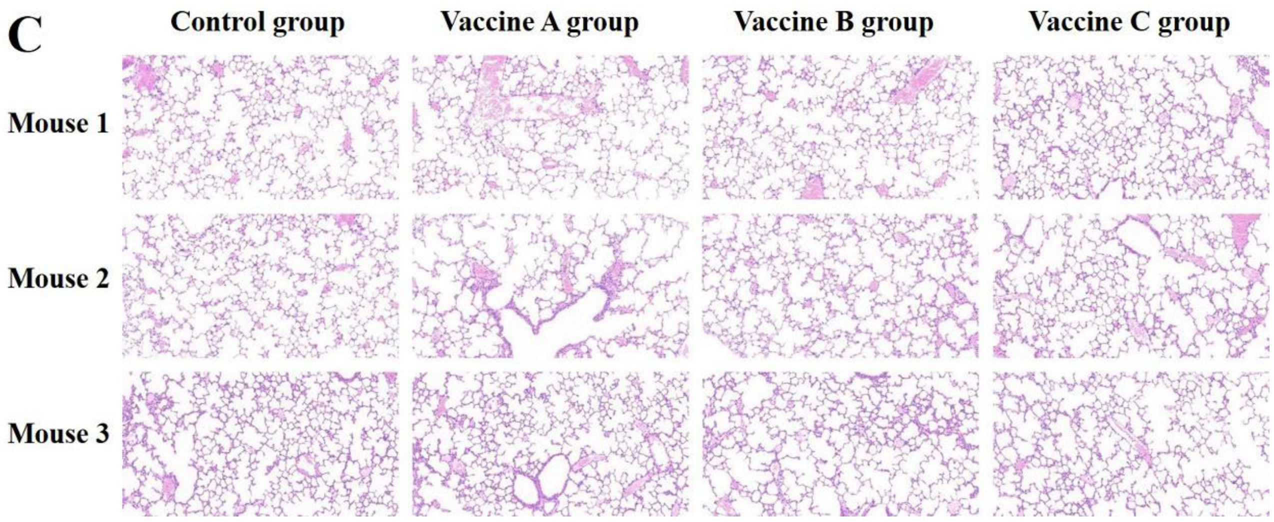

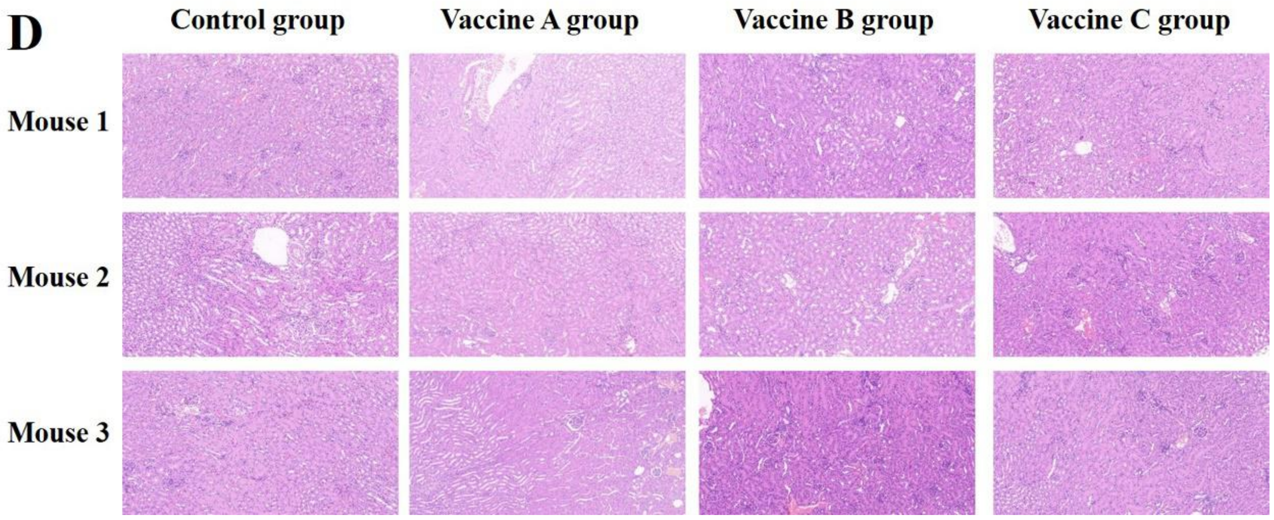

Figure S12: The peptide cocktail vaccines have no visible toxicity on the organs. Seven days after the last booster, all mice were executed. Heart, liver, lung and kidney were taken out, immersed and were finally stained with Hematoxylin-Eosin. No obvious pathological damage was found in all organs in all groups. The representative HE staining of heart, liver, lung and kidney in each mouse from four groups were exhibited in **(A)**, **(B)**, **(C)** and **(D)**, respectively. Control group: normal saline plus PLGA-NPs; Vaccine A group: PLGA-NP/peptides vaccine; Vaccine B group: R848/peptides vaccine; Vaccine C group: Poly I: C/peptides vaccine.

Figure S13:

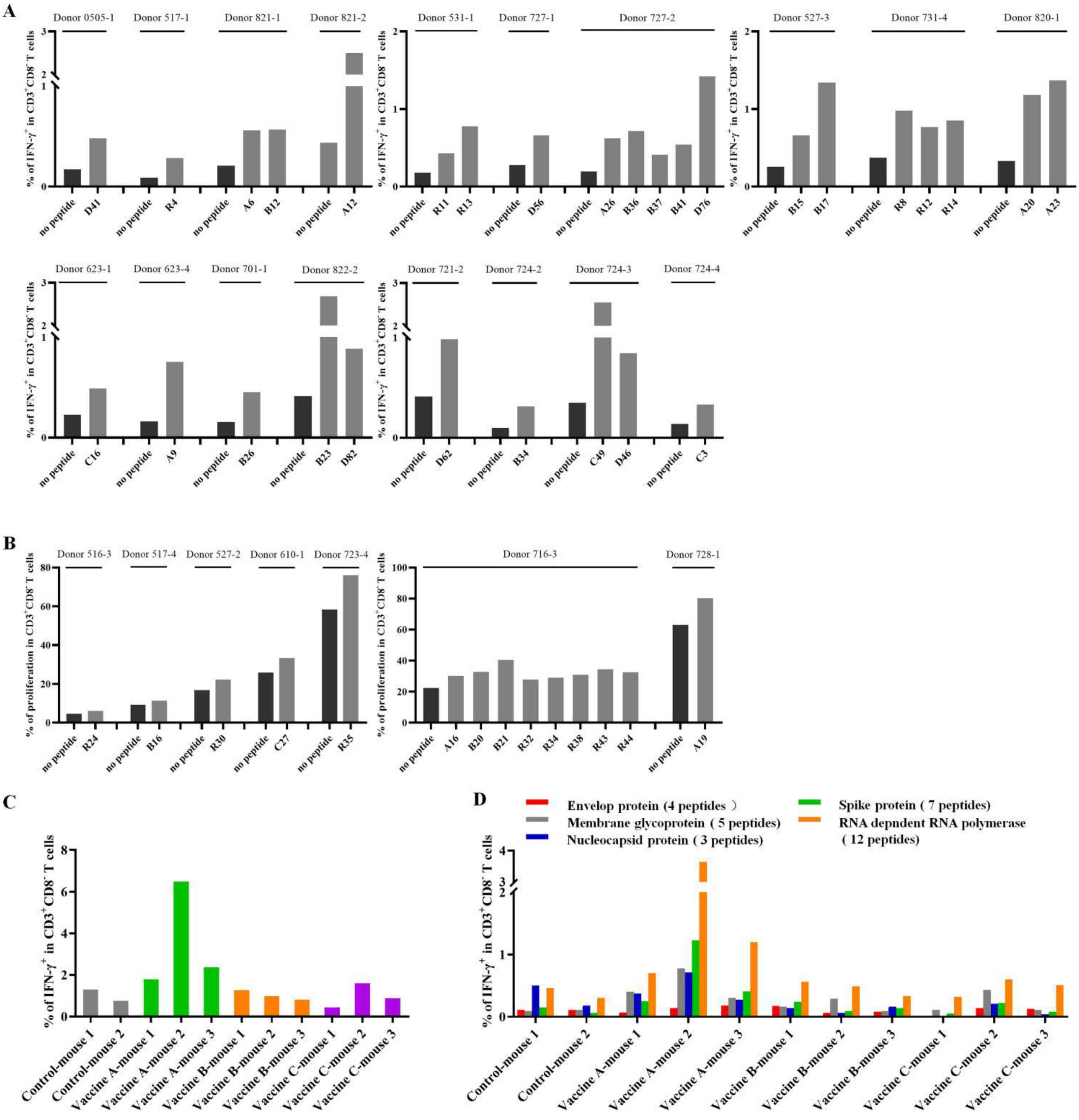

Figure S13: 44 CD8^{+} T cell epitope peptides elicited CD4^{+} T cell responses in DC-peptide-PBL co-cultures, but not in transgenic mice. DCs were induced for 7 days from unexposed healthy donor's PBMCs, then coincubated with candidate epitope peptides and autologous PBLs for 14 days. Cells were harvested and stimulated by corresponding candidate peptides for another 16 hours followed by IFN-γ ICS. In some co-culture wells,

the DC and peptides were co-cultured with CFSE-prelabeled PBLs for 14 days, and cells were then harvested to detect the proliferation percentage of T cell. **(A)** The frequency of IFN-γ^+ T cells in $CD3^+/CD8^-$ T cell population for each positive epitope peptide and each responded donor. **(B)** The proliferation percentage of T cells in $CD3^+/CD8^-$ cell population for each positive epitope peptide and each responded donor. Then, 31 VEPs restricted by HLA-A0201 were used to generate peptide cocktail vaccines in three formulations, and followed by three rounds vaccination of HLA-A0201/DR1 transgenic C57BL/6 mice. Splenocytes were collected 7 days after the last booster and *ex vivo* stimulated with five peptide pools (1 pool/protein) covering the 31 VEPs or with AFP peptides ($AFP_{158-166}$, $AFP_{424-432}$) as irrelevant control, or without peptide as negative control, and followed by IFN-γ ICS. **(C)** Total frequency of IFN-γ^+ T cells reacting to all peptide pools in $CD3^+/CD8^-$ cell population in each mouse. **(D)** Deconvolution of the total frequency in each mouse from C into the single SARS-CoV-2 protein. Control group: normal saline and PLGA-NPs; Vaccine A group: PLGA-NPs/peptides vaccine; Vaccine B group: R848/peptides vaccine; Vaccine C group: poly I: C/peptides vaccines.
